# Synthesis and Spectral Properties of *meso*-Arylbacteriochlorins, Including Insights into Essential Motifs of their Hydrodipyrrin Precursors

**DOI:** 10.3390/molecules22040634

**Published:** 2017-04-14

**Authors:** Muthyala Nagarjuna Reddy, Shaofei Zhang, Han-Je Kim, Olga Mass, Masahiko Taniguchi, Jonathan S. Lindsey

**Affiliations:** 1 Department of Chemistry, North Carolina State University, Raleigh, NC 27695-8204, USA; nmuthya@ncsu.edu (M.N.R.); szhang16@ncsu.edu (S.Z.); mass.olga@gmail.com (O.M.); mtanigu@ncsu.edu (M.T.); 2 Department of Science Education, Gongju National University of Education, Gongju 314-701, Korea

**Keywords:** self-condensation, bacteriochlorophyll, near-infrared, tetrahydrodipyrrin, dihydrodipyrrin, absorption, fluorescence, oxocarbenium ion, resonance, oxidation

## Abstract

Synthetic bacteriochlorins—analogues of bacteriochlorophylls, Nature’s near-infrared absorbers—are attractive for diverse photochemical studies. *meso*-Arylbacteriochlorins have been prepared by the self-condensation of a dihydrodipyrrin–carbinol or dihydrodipyrrin–acetal following an Eastern-Western (E-W) or Northern-Southern (N-S) joining process. The bacteriochlorins bear a gem-dimethyl group in each pyrroline ring to ensure stability toward oxidation. The two routes differ in the location of the gem-dimethyl group at the respective 3- or 2-position in the dihydrodipyrrin, and the method of synthesis of the dihydrodipyrrin. Treatment of a known 3,3-dimethyldihydrodipyrrin-1-carboxaldehyde with an aryl Grignard reagent afforded the dihydrodipyrrin-1-(aryl)carbinol, and upon subsequent acetylation, the corresponding dihydrodipyrrin-1-methyl acetate (dihydrodipyrrin–acetate). Self-condensation of the dihydrodipyrrin–acetate gave a *meso*-diarylbacteriochlorin (E-W route). A 2,2-dimethyl-5-aryldihydrodipyrrin-1-(aryl)carbinol underwent self-condensation to give a *trans*-A_2_B_2_-type *meso*-tetraarylbacteriochlorin (N-S route). In each case, the aromatization process entails a 2e^−^/2H^+^ (aerobic) dehydrogenative oxidation following the dihydrodipyrrin self-condensation. Comparison of a tetrahydrodipyrrin–acetal (0%) versus a dihydrodipyrrin–acetal (41%) in bacteriochlorin formation and results with various 1-substituted dihydrodipyrrins revealed the importance of resonance stabilization of the reactive hydrodipyrrin intermediate. Altogether 10 new dihydrodipyrrins and five new bacteriochlorins have been prepared. The bacteriochlorins exhibit characteristic bacteriochlorophyll-like absorption spectra, including a Q_y_ band in the region 726–743 nm.

## 1. Introduction

**Figure 1:**
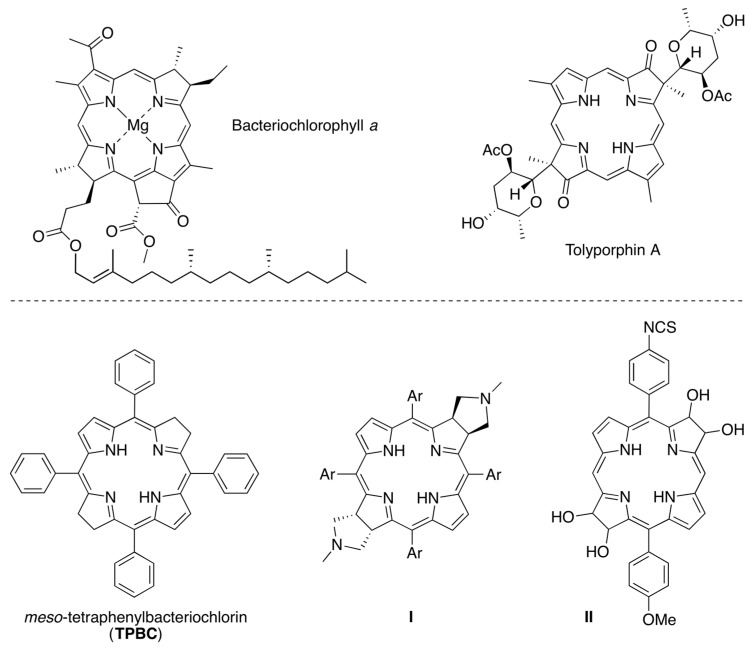
Bacteriochlorophyll *a*, tolyporphin A, and synthetic bacteriochlorins.

Bacteriochlorophyll *a* is the core pigment in the natural light-harvesting processes and electron-transfer reactions of anoxygenic phototrophic bacteria (Figure 1) [1]. Bacteriochlorophyll *a* features a strong long-wavelength absorption band in the near-infrared (NIR) region [1]. The spectroscopic properties of bacteriochlorophyll *a* stem from the tetrahydroporphyrin macrocycle, termed a bacteriochlorin [2]. Synthetic bacteriochlorins are attractive due to their strong NIR absorption, resembling that of bacteriochlorophyll *a*, yet also require amenability to tailoring to meet the molecular design objectives for various photochemical applications ranging from artificial photosynthesis to photomedicine.

A longstanding approach to prepare synthetic bacteriochlorins entails hydrogenation of a porphyrin [3,4]; one prominent example is provided by *meso*-tetraphenylbacteriochlorin [5,6] (Figure 1). While porphyrin hydrogenation is easily implemented, limitations of this approach include: (1) susceptibility of the tetrahydroporphyrin to adventitious dehydrogenation leading to the corresponding chlorin and porphyrin, (2) incompatibility with placement of auxochromes at the β-pyrrole positions for wavelength tuning, and (3) positional isomers if distinct patterns of *meso*-aryl groups are employed. Other methods for preparing bacteriochlorins include derivatization of porphyrins [7,8] yielding compounds such as **I** and **II**, or semisynthesis typically beginning with chlorophyll *a* or bacteriochlorophyll *a* [9,10]. Bacteriochlorophylls have not yet succumbed to total synthesis [11,12], but tolyporphin A diacetate, a derivative of one member of a family of dioxobacteriochlorins (Figure 1) isolated from a cyanobacterial culture [13,14], has been prepared in an elegant yet very lengthy synthesis [15,16,17].

**Scheme 1:**
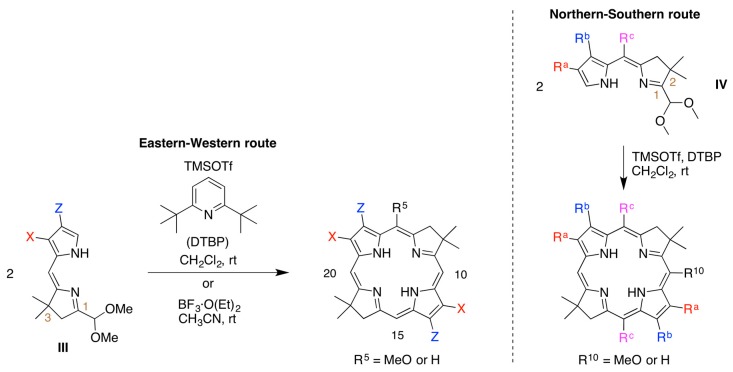
Two distinct approaches to synthetic bacteriochlorins.

As a compromise between the simplicity of porphyrin hydrogenation and the complexity of natural bacteriochlorin total synthesis, we have been developing *de novo* synthetic routes to stable, tailorable bacteriochlorins [18,19,20]. Such bacteriochlorins contain a gem-dimethyl group in each pyrroline ring to protect the macrocycle from adventitious oxidation. Two routes rely on the self-condensation of a dihydrodipyrrin–acetal, either in an “Eastern-Western” (E-W) [18] or “Northern-Southern” (N-S) fashion, defined by the respective 3- versus 2-position of the gem-dimethyl group in the pyrroline ring of the dihydrodipyrrin–acetal (compare **III** and **IV**, Scheme 1) [19]. The routes along with creative extensions by others [21,22,23,24,25,26,27,28] have enabled preparation of >100 bacteriochlorins with specific substituent patterns for diverse applications [29,30,31,32,33,34,35,36].

**Scheme 2:**
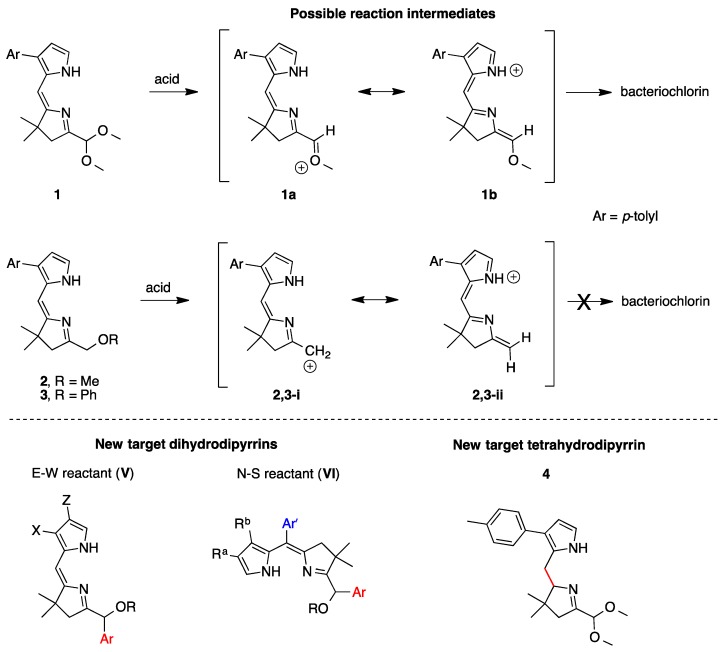
Possible reaction intermediates for conversion of dihydrodipyrrin–acetal **1** or dihydrodipyrrins **2** or **3** to the bacteriochlorin (**top**) and new target hydrodipyrrins (**bottom**).

The E-W and N-S routes both afford stable, gem-dimethyl-substituted bacteriochlorins yet differ in the method of preparation of the respective dihydrodipyrrin–acetals: the N-S route enables facile incorporation of alkyl or aryl substituents at the *meso*-position of the dihydrodipyrrin whereas such groups are not easily incorporated in the E-W route. In both routes, the 1-methyl position appears available for incorporation of an aryl group, but chemistry to introduce such groups has heretofore not been accomplished [33]. Regardless of synthetic issues, a key consideration is the nature of the reactive intermediates in the self-condensation leading to the bacteriochlorin. The reaction of **1** under the standard mild conditions (TMSOTf and 2,6-di-*tert*-butylpyridine (DTBP) in CH_2_Cl_2_ at room temperature) afforded bacteriochlorin in 41% yield [37] (consistent with 30%–46% under other conditions [18,31]). The oxocarbenium ion **1a** is a likely species in the head-to-tail dimerization process (Scheme 2) [33,38], although the extent to which the pyrrole participates in resonance-stabilization of the 1-oxocarbenium ion **1b** has remained unclear. Attempts to employ a dihydrodipyrrin bearing a single alkoxy substituent at the 1-methyl position (**2**, R = Me; **3**, R = Ph) under identical catalysis conditions (TMSOTf, DTBP in CH_2_Cl_2_) met with failure to form the bacteriochlorin (0% yield), which we attributed to the inability to form (**2**,**3-i**) or adequately stabilize (**2**,**3-ii**) the 1° carbenium ion [33]. We felt that incorporation of an electron-rich aryl group at the 1-methyl position would afford stabilization and enable synthesis of the corresponding *meso*-diarylbacteriochlorin.

In this paper, we describe the synthesis of several new dihydrodipyrrins wherein each is equipped with an aryl group and a single oxygen-containing substituent (hydroxy or acetoxy) at the 1-methyl group rather than the traditional acetal unit (Scheme 2). The dihydrodipyrrins incorporate the gem-dimethyl group at the 3-position for E-W condensations (**V**) or the 2-position for N-S condensations (**VI**), respectively. We then report studies concerning the reactivity of the dihydrodipyrrins (under conditions encompassing acids, solvents, and reaction time) for bacteriochlorin formation. One tetrahydrodipyrrin–acetal (compound **4**) also has been prepared for comparison with the analogous dihydrodipyrrin–acetal **1** to understand the structural requirements for bacteriochlorin formation. To delineate the spectral effects of *meso*-aryl substitution, the absorption and fluorescence spectroscopic properties of three new bacteriochlorins (with two or four *meso*-aryl groups) are reported and compared with those of eight known bacteriochlorins. The fluorescence properties (fluorescence spectrum, fluorescence quantum yield) of four of the latter bacteriochlorins have not been previously examined. Here, the fluorescence properties are reported for the three new and four known synthetic bacteriochlorins.

## 2. Results

### 2.1. Reconnaissance

**Scheme 3:**
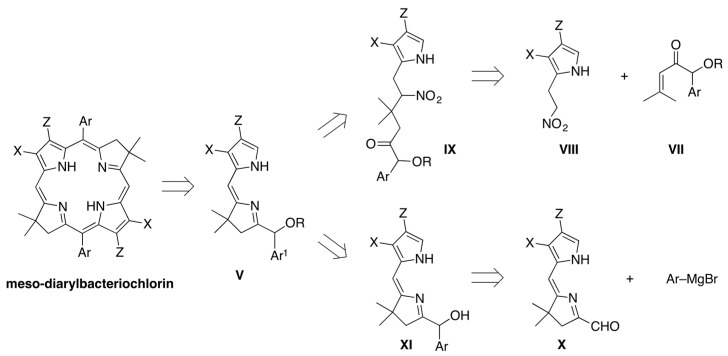
Retrosynthetic analysis for incorporating *meso*-aryl groups via the E-W synthesis.

To gain access to *meso*-diarylbacteriochlorins in an E-W condensation, or *meso*-tetraarylbacteriochlorins in a N-S condensation, requires introduction of an aryl group at the dihydrodipyrrin 1-methyl site, and hence modification of the acetal (1,1-dimethoxymethyl) unit otherwise present at the 1-position (Scheme 3).

Prior synthetic efforts to install an aryl group for E-W synthesis (**V**), wherein the aryl group was incorporated at an early stage of the reaction (**VII** + **VIII**), were unsuccessful. The failure of the synthesis occurred upon attempted McMurry-type reductive cyclization (conversion of **IX** → **V**; Scheme 3); hence the conversion of the target dihydrodipyrrin **V** to the bacteriochlorin remained untested. Given this backdrop, we turned to examine an approach wherein a dihydrodipyrrin–carboxaldehyde **X** is treated with an aryl Grignard reagent to introduce the aryl group at the 1-methyl position. The resulting dihydrodipyrrin-1-carbinol (**XI**) bearing an α-aryl group can then be subjected to self-condensation or further derivatized.

### 2.2. Synthesis of Dihydrodipyrrin–Carbinols and Dihydrodipyrrin–Acetates

**Scheme 4:**
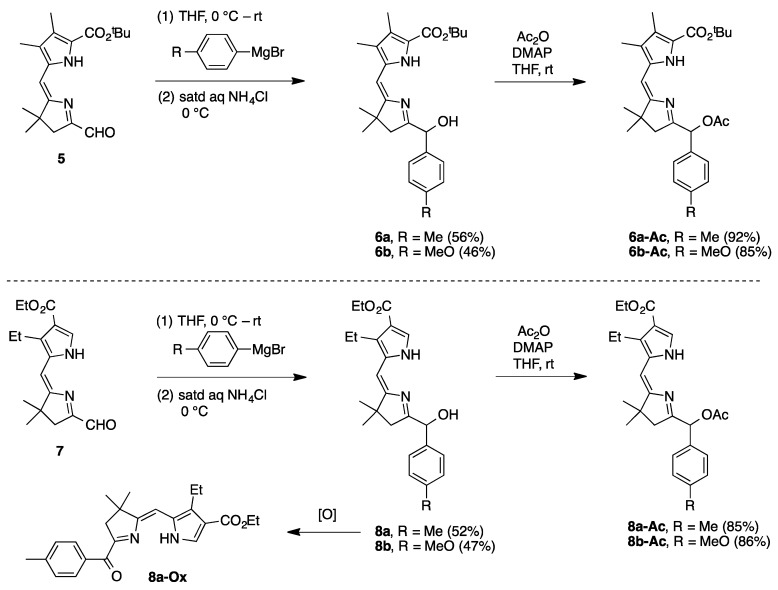
Synthesis of dihydrodipyrrin–carbinols and dihydrodipyrrin–acetates.

A wide variety of dihydrodipyrrin-carboxaldehydes are now known and could be examined for reaction with an aryl Grignard reagent. Dihydrodipyrrin–carboxaldehyde **5** bears two β-methyl groups and a stabilizing α-ester unit [36]. Treatment of **5** with commercially available *p*-tolylmagnesium bromide gave the target dihydrodipyrrin–carbinol **6a** in 56% yield (Scheme 4). The reaction required 3.5 equiv of Grignard reagent for completion, in part due to the pyrrole NH unit, which is expected to consume one equiv of Grignard reagent. The carbinol **6a** was subjected to acylation with acetic anhydride in the presence of DMAP, affording dihydrodipyrrin–acetate **6a-Ac** in 92% yield (compound **6a-Ac** (and analogues; *vide infra*) is formally a dihydrodipyrrin-1-methyl acetate but is referred to hereafter in shorthand as a dihydrodipyrrin–acetate). The dihydrodipyrrin–acetate **6a-Ac** was stable as a solid as well as for several weeks in CDCl_3_ solution. Similarly, the reaction of **5** with *p*-anisylmagnesium bromide gave the dihydrodipyrrin–carbinol **6b** in 46% yield, followed by acylation with Ac_2_O/DMAP to give dihydrodipyrrin–acetate **6b-Ac** in 85% yield. In all cases, workup of the Grignard reaction affording the dihydrodipyrrin–carbinol was achieved by treatment of the cooled reaction mixture with saturated aqueous NH_4_Cl.

Dihydrodipyrrin-carboxaldehydes bearing β-substituents other than dialkyl can be handled without the presence of an α-ester substituent. Thus, treatment of dihydrodipyrrin–carboxaldehyde **7** [34] with *p*-tolylmagnesium bromide gave dihydrodipyrrin–carbinol **8a** in 52% yield, followed by acetylation with Ac_2_O/DMAP to form dihydrodipyrrin–acetate **8a-Ac** in 85% yield (Scheme 4). Similar reaction of **7** with *p*-anisylmagnesium bromide followed by acetylation gave dihydrodipyrrin–acetate **8b-Ac**. In each case, the modest yield of the dihydrodipyrrin–carbinol may stem in part due to the presence of the corresponding ketone, formed upon oxidation on standing. The corresponding dihydrodipyrrin–acetates were stable in solid form and in solution. The ketone **8a-Ox** (a 1-*p*-toluoyl–dihydrodipyrrin) derived from dihydrodipyrrin–carbinol **8a** was isolated and characterized by ^1^H-NMR spectroscopy, ESI-MS, and absorption spectroscopy; the latter exhibited longer wavelength absorption (451 nm) due to an increase in conjugation compared to **8a** (340 nm). In summary, this route to aryl-substituted dihydrodipyrrin–carbinols is admittedly wasteful of the aryl Grignard reagent, but the latter is typically far less valuable than the dihydrodipyrrin–carboxaldehyde. Indeed, the aryl Grignard reagents employed herein were readily obtained in ample quantities from commercially suppliers.

### 2.3. Synthesis of Meso-Diarylbacteriochlorins (via E-W Route)

**Scheme 5:**
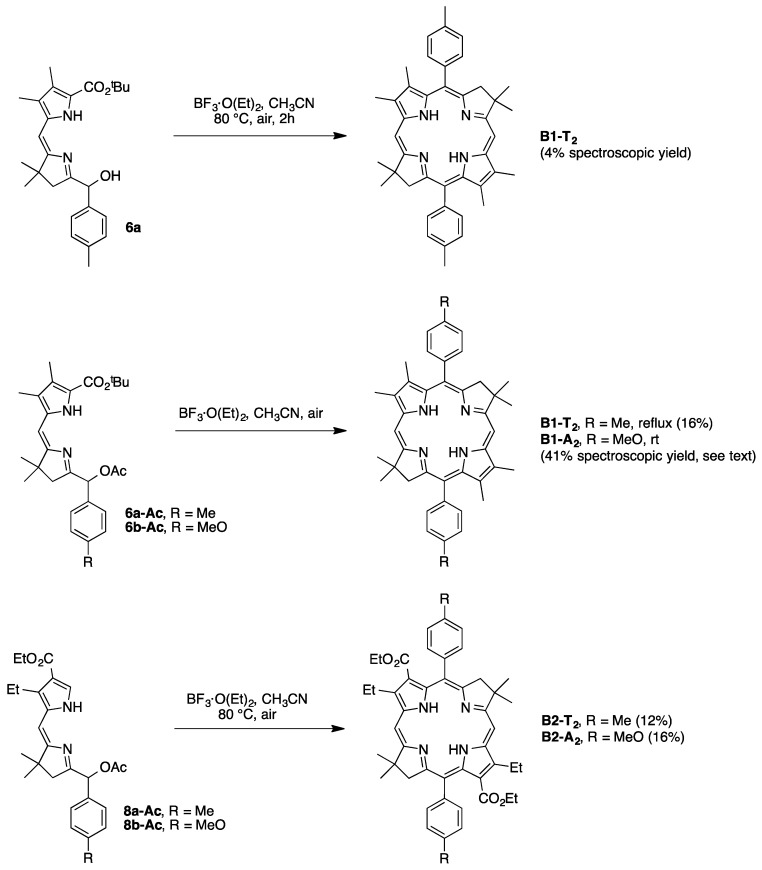
Self-condensation of dihydrodipyrrin–acetates.

The self-condensation of **6a** was first surveyed in CH_3_CN containing BF_3_·O(Et)_2_, after which **6a-Ac** (18 mM) was examined under a variety of acid catalysis conditions that have proved viable with dihydrodipyrrin–acetals (including BF_3_·O(Et)_2_ in CH_3_CN, TMSOTf and DTBP in CH_2_Cl_2_, and neat TFA) typically at room temperature open to air (see Appendix A for the results from the survey herein, as well as literature references to the origin and development of these catalysis conditions). The reaction was carried out with exposure to air given the necessity for a 2e^−^/2H^+^ oxidation in bacteriochlorin formation. The reactions were monitored by absorption spectroscopy and laser-desorption mass spectrometry (LD-MS). The yield of diarylbacteriochlorin in crude samples was assessed by absorption spectroscopy of the Q_y_ band (~726 nm, assumed ε_Qy_ = 120,000 M^−1^·cm^−1^ [18]) without isolation (“spectroscopic yield”). The best conditions identified for dihydrodipyrrin–carbinol **6a** (140 mM BF_3_·O(Et)_2_ in CH_3_CN at reflux for 2 h exposed to air) afforded *meso*-di-*p*-tolylbacteriochlorin **B1-T_2_** in 4.3% spectroscopic yield (Scheme 5). On the other hand, dihydrodipyrrin–acetate **6a-Ac** gave **B1-T_2_** in 22% spectroscopic yield under the same conditions.

The spectroscopic yield is determined without purification and isolation of the product, a longstanding method in tetrapyrrole chemistry enabled by the intense and characteristic absorption bands of the tetrapyrrole macrocycle. Such bands appear in the near-ultraviolet, visible, and near-infrared region for bacteriochlorins, are typically well-separated from—and more intense than—absorption bands due to impurities; accordingly, the presence and yield of the bacteriochlorin can be assessed even in low yields in the presence of a large quantity of impurities including reaction intermediates and unreacted starting material.

To isolate the bacteriochlorin, the self-condensation of dihydrodipyrrin–acetate **6a-Ac** was carried out at ~30-fold increased scale (0.069 mmol, 32 mg), whereupon **B1-T_2_** was obtained in 16% yield (3.3 mg) (Scheme 5). In this and subsequent examples, the isolated yield is invariably lower than the spectroscopic yield, a phenomenon that is attributed to losses upon purification and handling of small quantities of product. In this regard, the spectroscopic yield provides an unvarnished view of the potential of the chemistry. Unless noted otherwise, the reported yield in each case is the isolated yield. The bacteriochlorin was characterized by ^1^H-NMR spectroscopy, ESI-MS, LD-MS, and absorption spectroscopy. Bacteriochlorin **B1-T_2_**, which bears four methyl and two *p*-tolyl groups, is stable as a solid but slowly decomposed upon standing in CDCl_3_, despite treatment with K_2_CO_3_. The similar self-condensation of dihydrodipyrrin–acetate **6b-Ac** afforded *meso*-di-*p*-anisylbacteriochlorin **B1-A_2_** in 25% spectroscopic yield. An increase (37% spectroscopic yield) was observed upon prolonging the reaction time to 4 h at reflux, or performing the reaction at room temperature for 24 h (41% spectroscopic yield). Limited solubility thwarted chromatographic purification, and attempts to purify by washing with organic solvents or crystallization also failed to give a pure product. The bacteriochlorin **B1-A_2_** was characterized by absorption spectroscopy and MALDI-MS (matrix POPOP [39]).

Dihydrodipyrrin–acetates **8a-Ac** and **8b-Ac** differ from **6a-Ac** and **6b-Ac** in the presence of a pyrrole β-carbethoxy group and the absence of a pyrrole α-ester substituent. The self-condensation of **8a-Ac** in CH_3_CN containing BF_3_·O(Et)_2_ at room temperature for 24 h gave bacteriochlorin in 3% yield by absorption spectroscopy. Under reflux for 4 h, bacteriochlorin was observed in 20% yield (absorption spectrum) and the isolated yield of *meso*-di-*p*-tolylbacteriochlorin **B2-T_2_** was 12% (Scheme 5). The reactivity of **8a-Ac** is lower than that of dihydrodipyrrin–acetate **6a-Ac**, as longer reaction time was required under reflux, and the reaction at room temperature was sluggish. The self-condensation of **8b-Ac** under BF_3_·O(Et)_2_ at room temperature for 24 h gave bacteriochlorin **B2-A_2_** (16%), and **8a-Ac** gave **B2-T_2_** (3%) under similar conditions. The self-condensation under reflux for 2 h also gave **B2-A_2_** (28% spectroscopic yield; 16% isolated yield).

The greater yield in the self-condensation of dihydrodipyrrin–acetate **6a-Ac** (16%) versus dihydrodipyrrin–carbinol **6a** (4.3%) prompted examination of other, perhaps better leaving groups. However, attempts at *O*-derivatization of **8a** with tosyl chloride, mesyl chloride, trichloroacetic anhydride, trifluoroacetic anhydride, and triflic anhydride in the presence of various bases (DMAP, Et_3_N, pyridine, DBU, NaOH, NaH) did not afford a clean reaction. By contrast, acylation of **8a** with acetic anhydride in the presence of DMAP gave a fairly clean reaction and the product was obtained in 85% yield. Two noteworthy points even for reaction with Ac_2_O/DMAP are that the reaction time should be short and should be carried out in non-chlorinated solvents, otherwise the oxidized product (e.g., **8a-Ox**) predominates.

### 2.4. Synthesis of a Meso-Tetraarylbacteriochlorin (via the N-S Route)

**Scheme 6:**
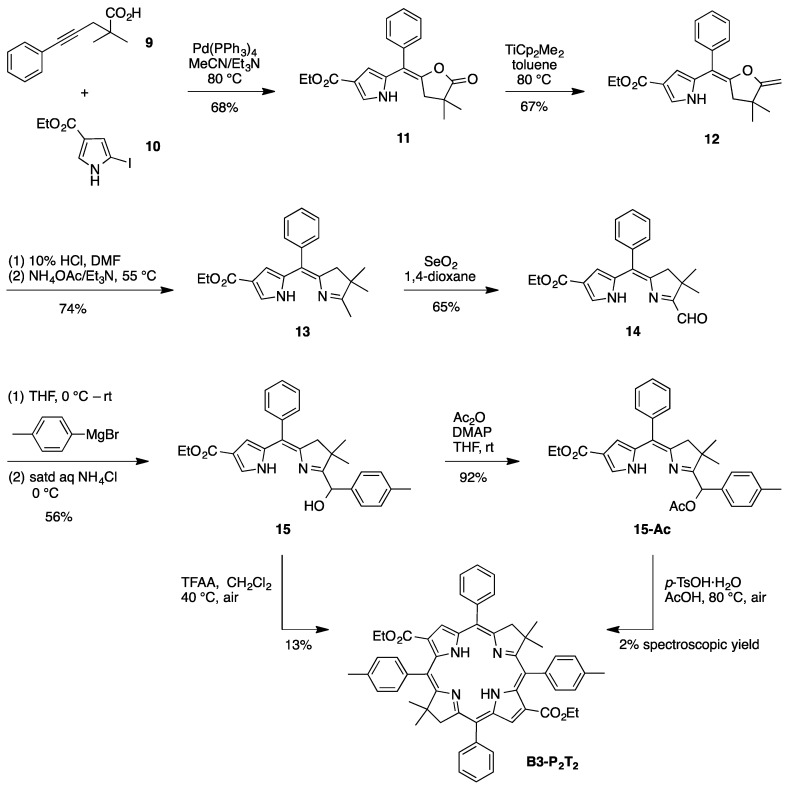
Synthesis of a *meso*-tetraarylbacteriochlorin (**B3-P_2_T_2_**).

We turned our attention to the synthesis of a *meso*-tetraarylbacteriochlorin (**B3-P_2_T_2_**). Access to **B3-P_2_T_2_** can be achieved in principle by pre-installation of one aryl group at the dihydrodipyrrin meso position, and the other at the α-methyl position. Such an approach requires use of the N-S route. Thus, the Pd-mediated coupling of acid precursor **9** [40] and iodopyrrole **10** [19] gave the lactone–pyrrole **11** in 68% yield. Treatment of the latter with the Petasis reagent afforded **12** in 67% yield. Hydrolysis of **12** and a subsequent Paal-Knorr type ring closure gave dihydrodipyrrin **13** as the major compound, accompanied by a very minor amount of the corresponding *E*-isomer. Oxidation of **13** with SeO_2_ furnished dihydrodipyrrin–carboxaldehyde **14** in 65% yield. Treatment of **14** with *p*-tolylmagnesium bromide afforded the dihydrodipyrrin–carbinol **15** in 56% yield. Subsequent acetylation with acetic anhydride/DMAP gave the desired dihydrodipyrrin–acetate **15-Ac** in 92% yield (Scheme 6).

The self-condensation of **15-Ac** following the conditions identified above (r.t. or 80 °C) did not furnish any bacteriochlorin, which prompted examination of a variety of acid catalysts (see Appendix A). The best conditions identified (*p*-TsOH·H_2_O, AcOH, 80 °C, in air) gave tetraarylbacteriochlorin **B3-P_2_T_2_** in up to 2% spectroscopic yield. As an alternative route, dihydrodipyrrin–carbinol **15** was examined for self-condensation but no bacteriochlorin was observed upon use of neat TFA. Upon use of trifluoroacetic anhydride (TFAA), dihydrodipyrrin–carbinol **15** in CH_2_Cl_2_ gave the tetraarylbacteriochlorin **B3-P_2_T_2_** in up to 17% spectroscopic yield (see Appendix A) and 13% isolated yield (Scheme 6). The reaction likely proceeds via the dihydrodipyrrin-1-methyl trifluoroacetate intermediate (not shown) formed in situ. Tetraarylbacteriochlorin **B3-P_2_T_2_** was characterized by ^1^H-NMR spectroscopy, ESI-MS, MALDI-MS and absorption spectroscopy. The absorption and fluorescence properties of the bacteriochlorins are described in the final section.

### 2.5. A Tetrahydrodipyrrin for Bacteriochlorin Formation

**Scheme 7:**
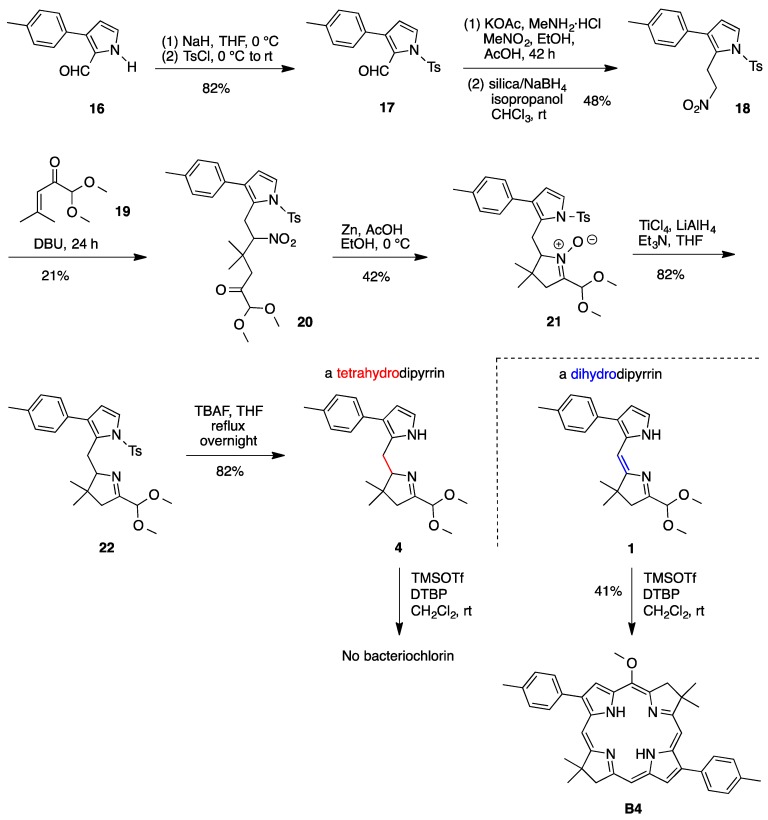
Synthesis of a tetrahydrodipyrrin–acetal.

In chlorin synthetic chemistry, tetrahydrodipyrrins—lacking a double bond bridging the pyrrole and pyrroline motifs—have proved superior to dihydrodipyrrins [41]. The origin of the superiority has been regarded to stem from the greater stability upon handling of tetrahydrodipyrrins versus dihydrodipyrrins. Hence, we sought to examine the use of a tetrahydrodipyrrin–acetal for comparison with the dihydrodipyrrin–acetal in conversion to the bacteriochlorin. The synthesis of a tetrahydrodipyrrin–acetal follows the procedure established previously for a tetrahydrodipyrrin–acetal lacking a *p*-tolyl group [42]. Deprotonation of pyrrole-2-carboxaldehyde **16** [31] with sodium hydride followed by treatment with *p*-tosyl chloride afforded **17** in 82% yield (Scheme 7). Subsequent nitro–aldol condensation followed by reduction with NaBH_4_ [43] afforded nitroethylpyrrole **18** in 48% yield. The Michael addition of **18** and α,β-unsaturated ketone **19** [33] in the presence of DBU for 24 h at room temperature afforded **20** in 21% yield. Reductive cyclization of **20** in the presence of zinc dust in ethanolic acetic acid afforded tetrahydrodipyrrin-*N*-oxide **21** in 42% yield. The use of ammonium formate [43] rather than ethanolic acetic acid was found to lead to the loss of the acetal moiety [42]. Deoxygenation of **21** with TiCl_4_ and LiAlH_4_ gave **22** in 82% yield. The *p*-tosyl group was removed when **22** was treated overnight with TBAF at room temperature, thereby affording **4** in 82% yield. The use of *p*-tosyl protection may be a significant beneficial factor in the synthesis of **4**, particularly in the deoxygenation of the pyrroline-*N*-oxide (**21** → **22**); similar deoxygenation of an analogue lacking both the *p*-tolyl group and *p*-tosyl protection proceeded in only 8% yield [42] versus 82% here.

With tetrahydrodipyrrin–acetal **4** in hand, comparison with the reaction of dihydrodipyrrin–acetal **1** was examined. The self-condensation conditions (5 equiv of TMSOTf and 20 equiv of DTBP in CH_2_Cl_2_) employed [31] for diverse dihydrodipyrrin–acetals were modified to use a lesser amount of reagents (4 equiv of TMSOTf and 8 equiv of DTBP), which emerged from a factorial design study [37]. Application of the latter conditions with dihydrodipyrrin–acetal **1** [18,31] gave the known [18] 5-methoxy-8,8,18,18-tetramethyl-2,12-di-*p*-tolylbacteriochlorin (**B4**) in 41% yield [37], whereas tetrahydrodipyrrin–acetal **4** decomposed and gave no **B4** or any other bacteriochlorin. The sole difference between **1** and **4** is the presence of a double bond rather than a single bond linking the pyrrole and pyrroline units of the hydrodipyrrin.

### 2.6. Spectroscopic Properties

**Figure 2:**
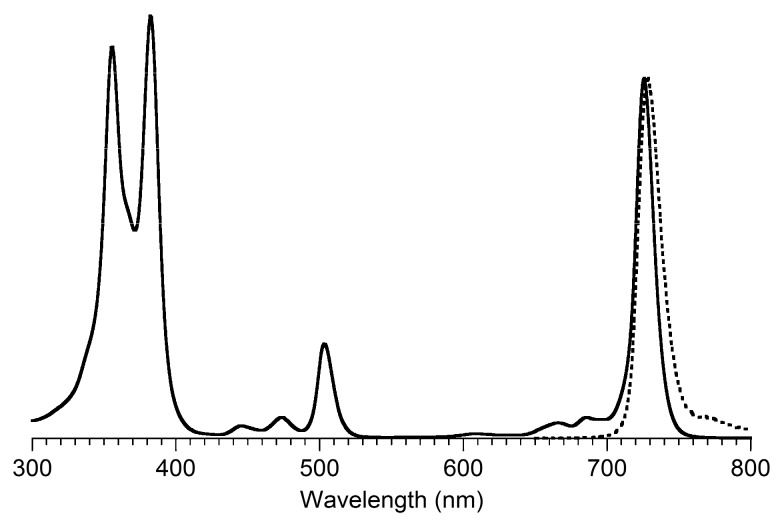
Absorption spectrum (solid line) and fluorescence spectrum (dotted line; λ_exc_ 503 nm) of *meso*-di-*p*-tolylbacteriochlorin **B1-T_2_** in toluene at room temperature.

**Figure 3:**
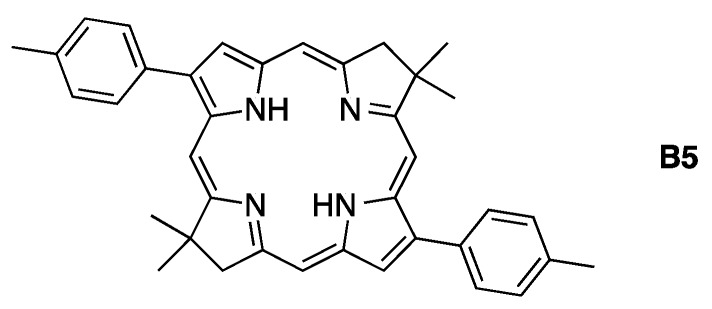
Bacteriochlorin used as a fluorescence standard.

The absorption and fluorescence features of the *meso*-arylbacteriochlorins are summarized in Table 1. The absorption and fluorescence spectra of *meso*-di-*p*-tolylbacteriochlorin **B1-T_2_** displayed in Figure 2 are representative. The fluorescence spectrum of each bacteriochlorin was obtained upon illumination into the Q_x_ band of samples in toluene at room temperature. The Ф_f_ value was determined by comparison with 8,8,18,18-tetramethyl-2,12-di-*p*-tolylbacteriochlorin (**B5**, Figure 3), which has Ф_f_ = 0.14 [18,44].

The characteristic features of diverse synthetic bacteriochlorins include an absorption feature in the near-ultraviolet region (B bands); an absorption band in the green region (Q_x_ band); an absorption band >700 nm (Q_y_ band), which is often quite sharp with full-width-at-half maximum (fwhm) of ~20 nm; a commensurably sharp fluorescence band with small Stokes shift relative to the Q_y_ absorption band; and modest fluorescence quantum yield (Ф_f_) in the range 0.05–0.20. The *meso*-arylbacteriochlorins prepared herein largely display these features.

## 3. Discussion

New routes to tailored bacteriochlorins are required to fully access the NIR spectral region. *meso*-Arylbacteriochlorins are of interest in this regard. In this section, we discuss the following features of the chemistry: (1) summation of knowledge concerning the essential motifs in the hydrodipyrrin unit for successful conversion to the bacteriochlorin; (2) comparison with other routes in tetrapyrrole chemistry; (3) necessity for dehydrogenation in the reaction; (4) advantages and limitations of the synthesis; and (5) spectral properties of the *meso*-arylbacteriochlorins.

### 3.1. Essential Motifs

**Scheme 8:**
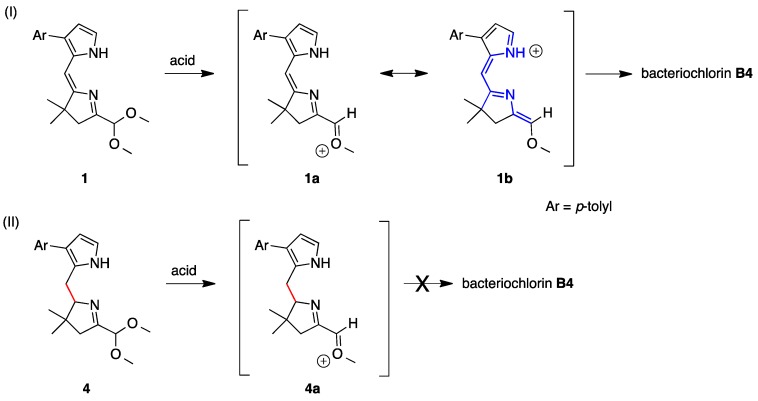
Resonance stabilization in a dihydrodipyrrin but not tetrahydrodipyrrin.

The failure of the tetrahydrodipyrrin–acetal (**4**) to form a bacteriochlorin, whereas the analogous dihydrodipyrrin–acetal (**1**) reacts smoothly, suggests the importance of a resonance-stabilized oxocarbenium ion intermediate (Scheme 8). The conjugation of the pyrrole and pyrroline units in the dihydrodipyrrin permits resonance delocalization (**1b**) whereas such conjugation is absent in the tetrahydrodipyrrin (**4a**). An alternative interpretation is that the coplanar architecture of the dihydrodipyrrin–acetal permits reaction whereas the tetrahedral carbons in the tetrahydrodipyrrin–acetal do not afford an architecture suitable for macrocyclization. While a possibility, we note that tetrahydrodipyrrins are used as the Western half in successful chlorin–forming reactions [41].

**Scheme 9:**
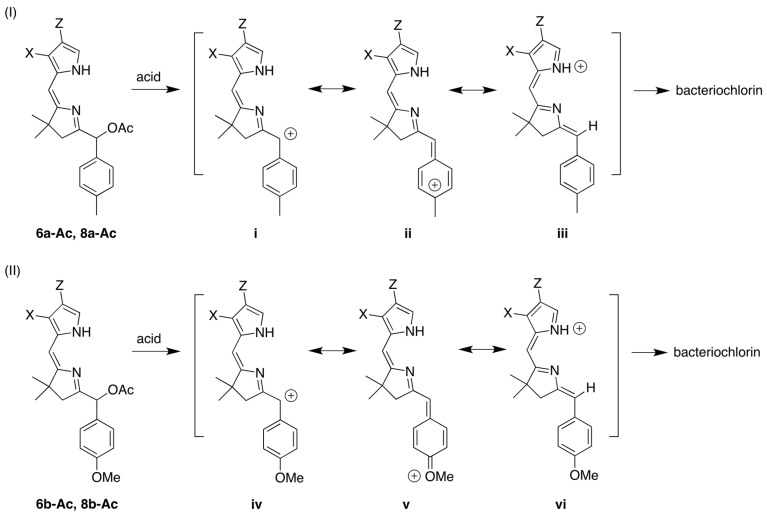
Putative intermediates *en route* to bacteriochlorins.

The acetal unit has been the dominant group installed at the dihydrodipyrrin 1-position, and also has been the object of the most extensive refinement, yet other groups also have been explored [33,36]. Here, substitution of the 1-methyl group with both an aryl group and a hydroxy or acetoxy substituent gave rise to the corresponding bacteriochlorin, which is attributed to the stabilization imparted by the aryl group, with *p*-anisyl superior to *p*-tolyl for obvious electronic reasons (Scheme 9). A comparison with the dihydrodipyrrin lacking an aryl group (**2** or **3**, Scheme 2) is at best tentative—the prior studies of **2** or **3** employed TMSOTf/DTBP and gave 0% yield, whereas the same conditions here with **6a** gave ~1% yield, yet self-condensation mediated by BF_3_·O(Et)_2_ in CH_3_CN at reflux with **6a-Ac** gave 22% spectroscopic yield (see Appendix A). A more extensive matrix of studies concerning substrates and conditions is required to draw firm conclusions, yet the body of data assembled to date suggests that more extensive resonance stabilization of the intermediate dihydrodipyrrin (versus the tetrahydrodipyrrin) facilitates formation of the corresponding bacteriochlorin.

### 3.2. Comparison of Routes

**Scheme 10:**
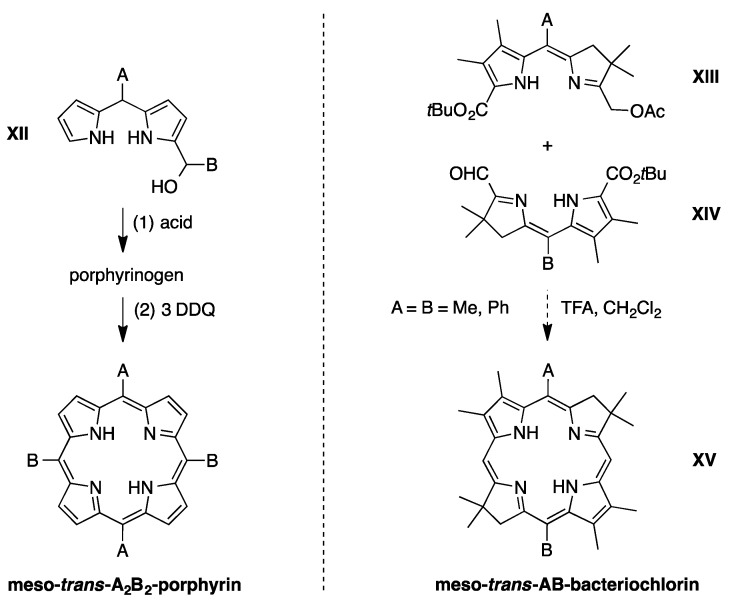
A dipyrromethane-carbinol affords a *meso*-*trans*-A_2_B_2_-porphyrin (**left**); two dihydrodipyrrins conceptually afford a *meso*-*trans*-AB-bacteriochlorin (**right**).

The dihydrodipyrrin–carbinols and dihydrodipyrrin–acetates employed herein are ostensibly similar to other compounds used in tetrapyrrole syntheses (Scheme 10). A dipyrromethane–carbinol (**XII**) undergoes self-condensation in the presence of acid to give the porphyrinogen, which upon 6e^−^/6H^+^ oxidation (e.g., by 3 DDQ) gives the corresponding *meso*-*trans*-A_2_B_2_-porphyrin [45,46]. Analogous routes have provided access to *meso*-aryltetrabenzoporphyrins (not shown) [47]. The Ph.D. thesis of O’Neal (under the guidance of Jacobi) describes the preparation of dihydrodipyrrins (**XIII**, **XIV**) for examination in routes to *meso*-*trans*-AB-bacteriochlorins (e.g., **XIII** + **XIV** → **XV**). One of the dihydrodipyrrins (**XIII**) bears an α-acetoxymethyl group, and both contain gem-dimethyl groups at the 2-position, engendering a N-S joining process. While pioneering in terms of dihydrodipyrrin synthetic methodology, to our knowledge a successful route to bacteriochlorins was not achieved [48].

### 3.3. Dehydrogenation

**Scheme 11:**
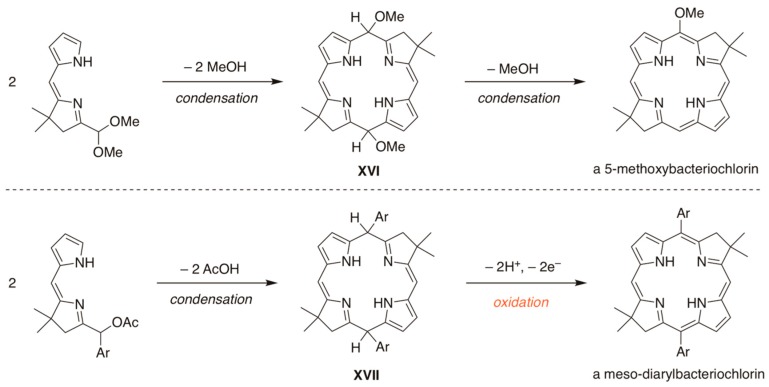
Oxidation is required in the reaction of a dihydrodipyrrin–acetate (**lower** panel) but not a dihydrodipyrrin–acetal (**upper** panel). (Substituents have been omitted for clarity.)

A dihydrodipyrrin–acetal is at the same oxidation level as the corresponding mono-methoxybacteriochlorin. A critical difference between the self-condensation of a dihydrodipyrrin–acetal and a dihydrodipyrrin–acetate is the requirement for dehydrogenation in the latter process: the dihydrodipyrrin–acetal self-condensation proceeds via the *trans*-dimethoxy-dihydrobacteriochlorin (**XVI**), which upon elimination of one molecule of methanol affords the monomethoxy-bacteriochlorin directly. In contrast, the dihydrodipyrrin–acetate affords the *trans*-diaryl-dihydrobacteriochlorin (**XVII**), which upon subsequent dehydrogenation (2e^−^/2H^+^ oxidation) gives the bacteriochlorin lacking any *meso*-methoxy groups (Scheme 11).

In chlorin syntheses, reduced precursors (e.g., a tetrahydrodipyrrin or a dihydrodipyrrin; and a dipyrromethane) are employed, whereupon in situ oxidation affords the chlorin macrocycle [41]. In porphyrin syntheses, diverse reaction pathways afford porphyrinogens (i.e., hexahydroporphyrins), which undergo smooth dehydrogenation (6e^−^/6H^+^ oxidation) to give the porphyrin macrocycle [46]. The dihydrobacteriochlorin (**XVII**) can equally be regarded as a bacteriochlorinogen precursor to the bacteriochlorin, in the same manner as a porphyrinogen is a precursor to the porphyrin. Thus, the use of reduced precursors in and of itself is not expected to cause adverse effects in the reactions of the various dihydrodipyrrin–carbinols and dihydrodipyrrin–acetates examined herein, although we note that in the synthesis of corrins, Eschenmoser emphasized the use of precursors at the same oxidation level as the target corrin macrocycles [49]. To gain a deep understanding of the role of oxidation state in forming bacteriochlorins may require study of a larger set of substrates, isolation of the corresponding hydrobacteriochlorin (i.e., bacteriochlorinogen) intermediates, and examination of the susceptibility of the latter toward dehydrogenation yielding bacteriochlorins.

### 3.4. Meso-Arylbacteriochlorins

The route described herein provides access to *meso*-*trans*-A_2_-bacteriochlorins and *meso*-*trans*-A_2_B_2_-bacteriochlorins, albeit with several present limitations. The limitations include (1) low yields of macrocycle formation; (2) requirement to use a Grignard reagent to install the aryl unit; and (3) likely restriction to use of electron-rich aryl units.

An alternative route to *trans*-A_2_-bacteriochlorins entails use of the N-S route. Advantages of the route described herein include the following: (1) use of the E-W route to construct the dihydrodipyrrin–carboxaldehyde on the path to *trans*-A_2_-bacteriochlorins; and (2) use of the N-S route to prepare *trans*-A_2_B_2_-bacteriochlorins, which have previously been inaccessible (although see Sutton et al. [50] for *meso*-*trans*-AB-bacteriochlorins bearing *vic*-diols at the pyrrole β-positions; e.g., **II** in Figure 1). Mono-arylbacteriochlorins have previously been prepared by selective bromination of a 5-methoxybacteriochlorin (from the E-W route) at the 15-position. Subsequent Pd-mediated coupling enabled installation of diverse aryl or other groups [29,32,35], whereas control of bromination was elusive with bacteriochlorins lacking a 5-alkoxy group [29]. Thus, routes to stable, gem-dimethylbacteriochlorins bearing 1, 2 or 4 *meso*-aryl groups have now been sketched out. The availability of such bacteriochlorins enables spectroscopic comparisons, as described in the next section.

### 3.5. Spectroscopic Properties

The synthetic *meso*-arylbacteriochlorins exhibit spectral features characteristic of members of the bacteriochlorophyll family. The *meso*-arylbacteriochlorins exhibit relatively sharp absorption and fluorescence bands along with Ф_f_ values in the range 0.1–0.2, all of which are typical of bacteriochlorins that lack aryl substituents. Tetrapyrrole macrocycles that are highly distorted typically exhibit bathochromically shifted absorption bands accompanied by low Ф_f_ values [51,52]. The *meso*-arylbacteriochlorins prepared herein do not fall into the family of highly distorted macrocycles, at least to the extent that the absorption and fluorescence properties are relevant proxies.

**Figure 4:**
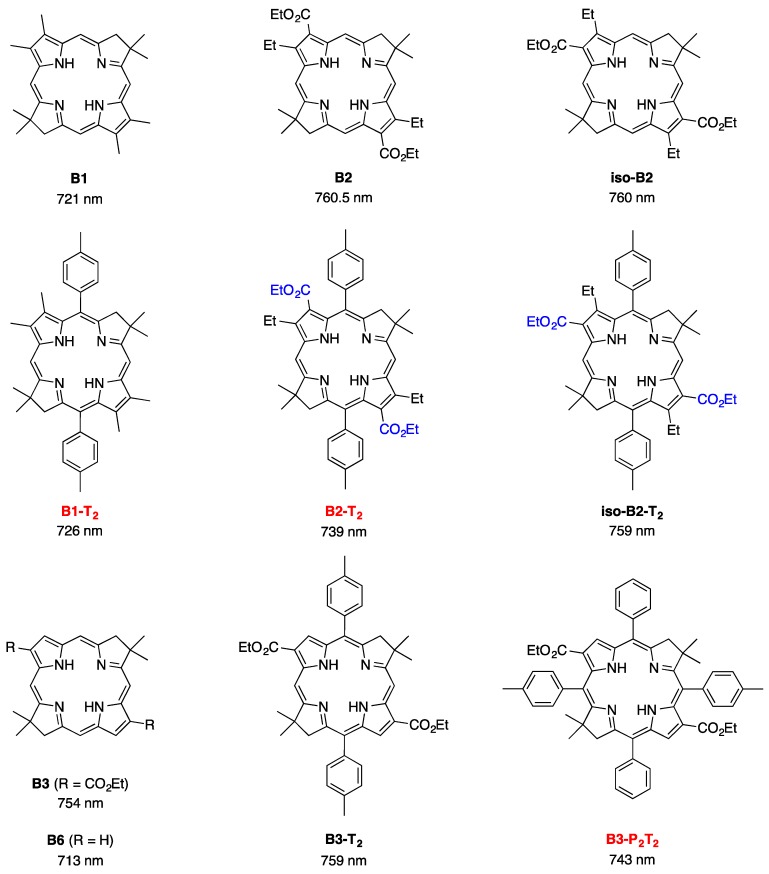
Ten bacteriochlorins for spectroscopic comparisons; the position of the long-wavelength (Q_y_) absorption band is included for ease of comparison. New compounds are labeled in red.

The presence of *meso*-aryl groups does give rise to noticeable shifts in band location and in one case slight broadening (but in no case are gross distortions of the spectra observed). Such effects are delineated here upon comparison against a set of synthetic bacteriochlorins that lack *meso*-aryl substituents. Here, the spectral properties of three new synthetic bacteriochlorins are compared with those of seven benchmark bacteriochlorins (Figure 4) as well as that of *meso*-tetraphenylbacteriochlorin (**TPBC**, Figure 1). The bacteriochlorins in Figure 4 enable incisive comparisons as follows.

(1) Bacteriochlorins **B1** [36], **B2** [31,44], **iso-B2** [19], **B3** [19], and **B6** [36] contain no *meso*-aryl groups and serve as benchmarks to gauge the effects of *meso*-aryl incorporation.
(2) Bacteriochlorins **B2** and **iso-B2** are positional isomers (due to swapping the positions of the β-ethyl and β-carbethoxy groups).
(3) Bacteriochlorins in the second row (**B1-T_2_**, **B2-T_2_**, and **iso-B2-T_2_** [19]) differ from the first row (**B1**, **B2**, **iso-B2**) in the presence of two *meso*-aryl groups.
(4) Bacteriochlorins in the third row (**B3**, **B3-T_2_** [19], and **B3-P_2_T_2_**) each contain two β-carbethoxy groups but differ in the number (0, 2, 4) of *meso*-aryl groups.

The absorption spectra of the 11 bacteriochlorins are summarized in Table 2 in pairwise fashion with the benchmark bacteriochlorin (above) and *meso*-arylbacteriochlorins (below). As a prelude to in-depth consideration of the effects of *meso*-aryl substituents on photophysical properties of bacteriochlorins, the following features warrant comment.

(1) Bacteriochlorin positional isomers **B2** and **iso-B2** exhibit absorption and fluorescence spectral properties that are nearly identical with each other.
(2) β-Carbethoxy groups are established auxochromes in bacteriochlorins [53]. The presence of two β-carbethoxy groups in **B3** (754 nm) causes a bathochromic shift of the Q_y_ absorption band by 41 nm compared to that of the unsubstituted benchmark bacteriochlorin **B6** (713 nm).
(3) Aryl groups also serve as auxochromes in bacteriochlorins upon incorporation at the *meso*- or β-positions. Two β-aryl groups (2,12- or 3,13-diarylbacteriochlorins) cause a bathochromic shift of the Q_y_ absorption band by ~23 nm relative to that of **B6** [30], whereas a single *meso*-aryl group typically causes a bathochromic shift of the Q_y_ absorption band by 3 to 6 nm [29].

**Figure 5:**
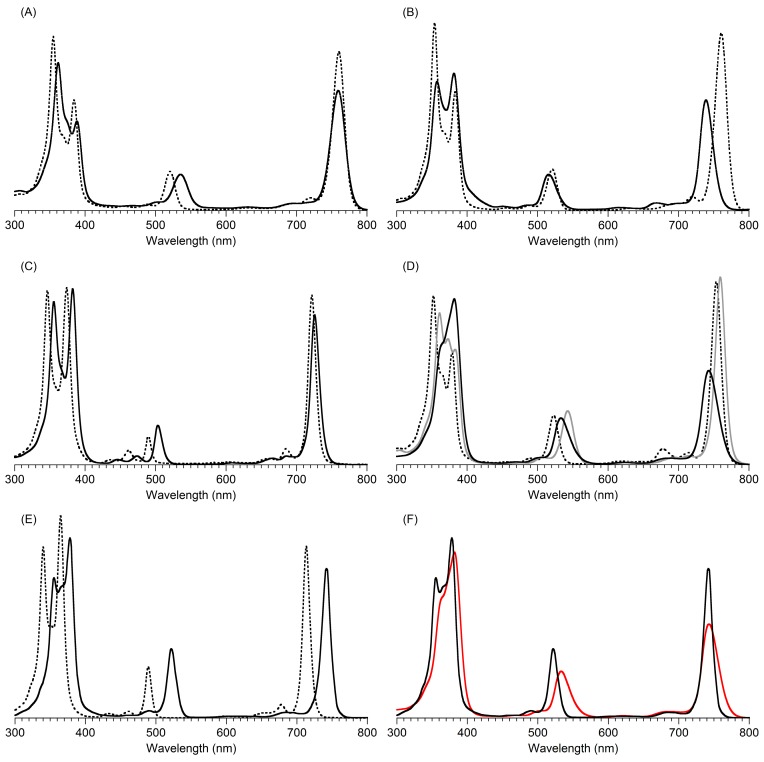
Absorption spectra of bacteriochlorins in toluene at room temperature. (**A**) **iso-B2** (dotted) and **iso-B2-T_2_** (solid); (**B**) **B2** (dotted) and **B2-T_2_** (solid); (**C**) **B1** (dotted) and **B1-T_2_** (solid); (**D**) **B3** (dotted), **B3-T_2_** (grey), and **B3-P_2_T_2_** (solid); (**E**) **B6** (dotted) and **TPBC** (solid); (**F**) **TPBC** (black, reprised from panel E) and **B3-P_2_T_2_** (red, reprised from panel D).

The absorption spectra of the bacteriochlorins are shown in Figure 5, where (1) spectra of the *meso*-arylbacteriochlorins are plotted in solid lines; and (2) benchmark bacteriochlorins (lacking *mes*o-aryl substituents) are plotted in dotted lines. The *y*-axis scales (integrated energy, see Materials and Methods) are set equal across the panels A to F to facilitate comparison of integrated absorption intensities. Comparison of the absorption spectra of **iso-B2** and **iso-B2-T_2_** highlights the effect of *meso*-aryl substituents (Figure 5, Panel A):

(1) The position of the Q_y_ absorption band is nearly unchanged, while a hypochromic change is observed together with an increase in the fwhm by 5 nm for **iso-B2-T_2_**.
(2) The B_y_, B_x_, and Q_x_ absorption bands exhibit bathochromic shifts (+7, +4, and +14 nm, respectively) for **iso-B2-T_2_**. At odds with this observation, the absorption spectra of **B2** and **B2-T_2_** are distinct from each other (Figure 5, Panel B). Compared to the benchmark bacteriochlorin **B2**, **B2-T_2_** shows a large (21 nm) hypsochromic shift of the Q_y_ absorption band, together with a large hypochromic change (~40% decline), while the fwhm is slightly increased. The positions of the B_y_, B_x_, and Q_x_ absorption bands of **B2** and **B2-T_2_** are relatively unchanged.
(3) The contrasting results for the meso-di-*p*-tolylbacteriochlorins **B2-T_2_** and **iso-B2-T_2_** are quite surprising, since the two are positional isomers, where the only structural difference is the swapped position of the β-ethyl and β-carbethoxy groups. Reasonable expectations are that the absorption spectra of **B2-T_2_** and **iso-B2-T_2_** should be nearly identical, as shown for their respective non-aryl analogues **B2** and **iso-B2**. The origin of the hypsochromic shift of the Q_y_ absorption band due to pairwise juxtaposition of a β-carbethoxy group and a *meso*-aryl substituent remains unclear.

The effects of *meso*-aryl substituents on the absorption spectra are further highlighted with additional examples. The absorption spectrum of **B1** (which lacks β-carbethoxy groups) is compared with that of the corresponding *meso*-di-*p*-tolylbacteriochlorin **B1-T_2_** (Figure 5, Panel C). The B_y_, B_x_, Q_x_, and Q_y_ absorption bands of **B1-T_2_** exhibit bathochromic shifts (+10, +8, +13, and +5 nm, respectively) compared to those of benchmark bacteriochlorin **B1**. Similar effects are observed upon comparison of **B3** and **B3-T_2_**, where the *p*-tolyl substituents are introduced at distal (non-adjacent) *meso*-positions relative to the β-carbethoxy groups (Figure 5, Panel D, see dashed and grey solid lines). The Q_x_ and Q_y_ absorption bands of **B3-T_2_** exhibit bathochromic shifts (+20 and +5 nm, respectively) compared to those of benchmark bacteriochlorin **B3** (but comparison of the B_y_, B_x_ bands is thwarted by the further apparent split into three peaks).

Moving from bacteriochlorin **B3-T_2_** to **B3-P_2_T_2_**, where two additional *meso*-*p*-tolyl substituents are present, juxtaposes the β-carbethoxy groups and the *meso*-*p*-tolyl substituents. As described above for **B2** and **B2-T_2_**, such an arrangement causes a significant hypsochromic shift of the Q_y_ absorption band. Indeed, the absorption spectra of **B3-T_2_** and **B3-P_2_T_2_** exhibit similar trends. Compared to **B3-T_2_**, bacteriochlorin **B3-P_2_T_2_** shows a large (16 nm) hypsochromic shift of the Q_y_ absorption band, together with a large hypochromic change (~50% decline) and an increase in fwhm (by 11 nm).

In summary, two examples now illustrate that the expected auxochromic effect of the β-carbethoxy groups is canceled by the *meso*-aryl substituents adjacent thereto (Figure 5, Panel B and Panel D). Such effects may accrue from structural and/or electronic factors, the origin of which will require structural studies and density functional theory calculations, as well as further examples.

A final comparison concerns the spectral properties of *meso*-tetraphenylbacteriochlorin (**TPBC**). The study of synthetic bacteriochlorins largely originated (ca. 1950s) with the first report of the preparation of *meso*-tetraphenylbacteriochlorin (**TPBC**) [54] and was significantly enabled by the subsequent synthetic method of Whitlock and coworkers [6]. The B_y_, B_x_, Q_x_, and Q_y_ absorption bands of **TPBC** exhibit bathochromic shifts (+15, +13, +33, and +29 nm, respectively) compared to those of benchmark bacteriochlorin **B6** (Figure 5, Panel E).

**Figure 6:**
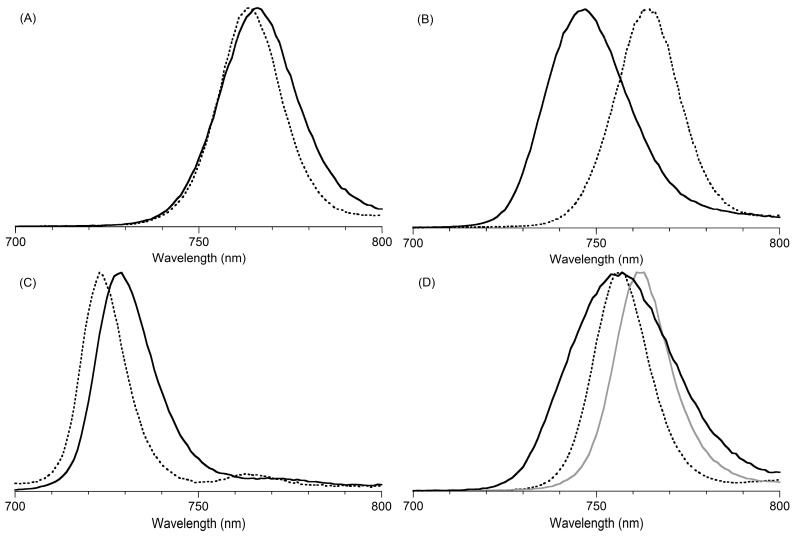
Fluorescence spectra of bacteriochlorins in toluene at room temperature. (**A**) **iso-B2** (dotted) and **iso-B2-T_2_** (solid); (**B**) **B2** (dotted) and **B2-T_2_** (solid); (**C**) **B1** (dotted) and **B1-T_2_** (solid); (**D**) **B3** (dotted), **B3-T_2_** (grey), and **B3-P_2_T_2_** (solid).

The fluorescence properties of the new *meso*-arylbacteriochlorins (**B1-T_2_**, **B2-T_2_**, **B3-P_2_T_2_**) and relevant benchmark bacteriochlorins are summarized in Table 3. The fluorescence properties of known bacteriochlorins **iso-B2**, **iso-B2-T_2_**, **B3** and **B3-T_2_** have not been previously reported whereas those of **B1** [36] and **B2** [44,53] are known. The seven newly reported Φ_f_ values here were determined with **B5** (Ф_f_ = 0.14 [18]) as a common reference standard, which also was used as the standard for **B2** [44,53]. The same value for **B2** was found upon use of an integrating sphere [36], an approach also employed for **B1** [36]. Hence, all of the values reported in Table 3 share a common reference and/or are self-consistent where distinct methods of determination were employed. The fluorescence spectra and yields were typically acquired upon excitation into the Q_x_ band near 500 nm. The fluorescence spectra of nine bacteriochlorins are shown in Figure 6. The major findings are as follows:

(1) The difference in position of the Q_y_ fluorescence band versus that of the benchmarks (Δ) corresponds very well to the difference of the Q_y_ absorption band compared to that of benchmarks (Δ), except for that of **B3-P_2_T_2_**. The position of the Q_y_ fluorescence band of **B3-P_2_T_2_** was unchanged compared to that of benchmark **B3**, which results in a large Stokes shift (*vide infra*).
(2) The Stokes shift of each benchmark bacteriochlorin lacking *meso*-aryl substituents is small (<70 cm^−1^ (<4 nm)). On the other hand, *meso*-arylbacteriochlorins exhibit larger Stokes shifts (>120 cm^−1^ (>7 nm)), and **B3-P_2_T_2_** shows the largest Stokes shift (250 cm^−1^ (12 nm)).
(3) The presence of *meso*-aryl substituents causes broadening of the fluorescence spectra. Such an effect is most pronounced for **B3-P_2_T_2_**, where the fwhm has almost doubled (34 nm, +16 nm) compared to that of benchmark bacteriochlorin **B3** (18 nm).
(4) The fluorescence quantum yield (Φ_f_) of *meso*-arylbacteriochlorins is increased (1.2–1.6 times) compared to that of the benchmarks lacking *meso*-aryl substituents.

In summary, the *meso*-arylbacteriochlorins exhibit absorption spectra, fluorescence spectra, and fluorescence quantum yields that are characteristic of exemplary members of the family of natural and synthetic bacteriochlorins, along with spectral shifts and broadening in specific instances.

## 4. Materials and Methods

### 4.1. General Methods

^1^H-NMR (300 MHz) and ^13^C-NMR (75 MHz) spectra were collected at room temperature in CDCl_3_ unless noted otherwise. Absorption spectra were obtained in toluene at room temperature unless noted otherwise. Electrospray ionization mass spectrometry (ESI-MS) data are reported for the molecular ion or protonated molecular ion. THF used in all reactions was freshly distilled from Na/benzophenone ketyl. The known, non-commercial compounds **5** [36], **7** [34], **9** [40], **10** [19], **16**, [31], and **19** [33] were prepared as described in the literature and confirmed for purity by ^1^H-NMR spectroscopy.

### 4.2. Self-Condensation Study

Following the procedure employed previously to gauge suitability of dihydrodipyrrin substrates [33,37], a solution of tetrahydrodipyrrin–acetal **4** (0.014–0.047 mmol, 18 mM) and DTBP (8 mol equiv) in anhydrous CH_2_Cl_2_ was treated with TMSOTf (4 molar equiv) at room temperature. The reaction mixture was stirred overnight, then diluted with CH_2_Cl_2_ and quenched by the addition of saturated aqueous NaHCO_3_. After extraction, the organic phase was washed (water, brine), dried (Na_2_SO_4_) and concentrated. The crude sample was analyzed for the presence of bacteriochlorin by TLC, LD-MS and absorption spectroscopy.

### 4.3. Synthesis and Characterization

*2,3,4,5-Tetrahydro-1-(dimethoxymethyl)-3,3-dimethyl-7-(4-methylphenyl)dipyrrin* (**4**). Following a reported procedure [42] with some modification, a sample of **22** (200 mg, 0.426 mmol) was treated with TBAF (1.3 mL, 1.3 mmol, 1.0 M in THF) under argon. The reaction mixture was stirred overnight at reflux. Saturated aqueous NaHCO_3_ was added, and the mixture was extracted with ethyl acetate. The organic extract was washed (brine), dried (Na_2_SO_4_), concentrated and chromatographed (silica, CH_2_Cl_2_/ethyl acetate (4:1)) to afford a yellow oil (118 mg, 82%): ^1^H-NMR δ 0.95 (s, 3H), 1.12 (s, 3H), 2.37 (s, 3H), 2.43, 2.51 (AB, *J* = 2.2 Hz, *J* = 17.4 Hz, 2H), 2.64 (ABX, *J* = 11.7 Hz, *J* = 15.2 Hz, 1H), 3.05 (ABX, *J* = 2.9 Hz, *J* = 15.2 Hz, 1H), 3.45 (s, 6H), 3.76–3.79 (m, 1H), 4.87 (s, 1H), 6.28–6.30 (m, 1H), 6.75–6.77 (m, 1H), 7.18 (d, *J* = 8.1 Hz, 2H), 7.31 (d, *J* = 8.1 Hz, 2H), 9.95 (br, 1H); ^13^C-NMR (100 MHz) δ 21.3, 22.9, 26.5, 27.3, 41.5, 48.7, 54.9, 80.4, 103.1, 108.3, 116.3, 121.2, 127.5, 128.2, 129.3, 134.7, 174.3; ESI-MS obsd 341.2225, calcd. 341.2224 [(M + H)^+^, M = C_21_H_28_N_2_O_2_].

*9-tert-Butoxycarbonyl-2,3-dihydro-1-[(hydroxy)(4-methylphenyl)methyl]-3,3,7,8-tetramethyldipyrrin* (**6a**). Following a literature procedure ([55]; see Supporting Information therein) with slight modification, a solution of **5** (97 mg, 0.29 mmol) in THF (1.9 mL) at 0 °C was treated with *p*-tolylmagnesium bromide (1.0 mL, 1.0 mmol, 3.5 equiv, 1.0 M in THF) over the course of 2 min. After 5 min further, the ice bath was removed, and the reaction mixture was stirred for 2 h. The reaction mixture was then cooled to 0 °C, whereupon saturated aqueous NH_4_Cl (4.0 mL) was added. The mixture was extracted with ethyl acetate. The organic extract was washed with water, dried (Na_2_SO_4_), and chromatographed (silica, ethyl acetate/hexanes (1:3)) to give a light brown solid (69 mg, 56%): m.p. 130–132 °C; ^1^H-NMR δ 1.11 (s, 3H), 1.20 (s, 3H), 1.59 (s, 9H), 2.05 (s, 3H), 2.29 (s, 3H), 2.36 (s, 3H), 2.30, 2.45 (AB, ^2^*J* =18.6 Hz, 2H), 3.86 (d, *J* = 4.2 Hz, 1H), 5.43 (d, *J* = 4.2 Hz, 1H), 5.81 (s, 1H), 7.18 (d, *J* = 8.0 Hz, 2H), 7.27 (d, *J* = 8.0 Hz, 2H), 10.65–10.72 (br, 1H); ^13^C-NMR δ 8.8, 10.2, 21.2, 28.5, 28.85, 28.94, 41.9, 48.8, 74.5, 80.0, 103.3, 119.2, 119.9, 126.7, 126.8, 129.4, 129.5, 136.5, 138.2, 160.7, 160.9, 181.2; ESI-MS obsd 423.2637, calcd. 423.2642 [(M + H)^+^, M = C_26_H_34_N_2_O_3_]; λ_abs_ (toluene) 360 nm.

*1-[(Acetoxy)(4-methylphenyl)methyl]-9-tert-butoxycarbonyl-2,3-dihydro-3,3,7,8-tetramethyldipyrrin* (**6a-Ac**). Following a procedure [48] with slight modification, a solution of **6a** (22 mg, 52 µmol) in THF (2.1 mL) at room temperature was treated with acetic anhydride (11 mg, 0.11 mmol) and DMAP (13 mg, 0.11 mmol). The reaction mixture was stirred for 2 h. Saturated aqueous NaHCO_3_ (2 mL) was added, and the mixture was extracted with ethyl acetate. The organic extract was washed with water, dried (Na_2_SO_4_), and chromatographed (silica, ethyl acetate/hexanes (1:4)) to give a light brown solid (22 mg, 92%): m.p. 159–161 °C; ^1^H-NMR δ 1.14 (s, 3H), 1.16 (s, 3H), 1.58 (s, 9H), 2.02 (s, 3H), 2.250 (s, 3H), 2.252 (s, 3H), 2.35 (s, 3H), 2.41, 2.55 (AB, ^2^*J* =18.3 Hz, 2H), 5.77 (s, 1H), 6.41 (s, 1H), 7.18 (d, *J* = 8.1 Hz, 2H), 7.33 (d, *J* = 8.1 Hz, 2H), 10.90–10.98 (br, 1H); ^13^C-NMR δ 8.8, 10.4, 20.0, 21.2, 28.6, 28.8, 41.1, 49.3, 75.7, 79.7, 103.5, 118.9, 120.3, 125.7, 127.4, 129.5, 130.0, 132.6, 138.8, 161.1, 161.9, 170.3, 176.8; ESI-MS obsd 487.2567, calcd. 487.2567 [(M + Na)^+^, M = C_28_H_36_N_2_O_4_]; λ_abs_ (toluene) 369 nm.

*9-tert-Butoxycarbonyl-2,3-dihydro-1-[(hydroxy)(4-methoxyphenyl)methyl]-3,3,7,8-tetramethyldipyrrin* (**6b**). Following a literature procedure ([55]; see Supporting Information therein) with slight modification, a solution of **5** (80 mg, 0.24 mmol) in THF (1.6 mL) at 0 °C was treated with *p*-anisylmagnesium bromide (0.85 mL, 0.85 mmol, 3.5 equiv, 1.0 M in THF) over the course of 5 min. After an additional 5 min, the ice bath was removed, and the reaction mixture was stirred for 2 h. The reaction mixture was then cooled to 0 °C, whereupon saturated aqueous NH_4_Cl (4.0 mL) was added. The mixture was extracted with ethyl acetate. The organic extract was washed with water, dried (Na_2_SO_4_), and chromatographed (silica, ethyl acetate/hexanes (1:3)) to give a light brown solid (48 mg, 46%): m.p. 52–54 °C; ^1^H-NMR δ 1.11 (s, 3H), 1.20 (s, 3H), 1.58 (s, 9H), 2.04 (s, 3H), 2.29 (s, 3H), 2.30, 2.44 (AB, ^2^*J* = 18.6 Hz, 2H), 3.81 (s, 3H), 3.91 (br, 1H), 5.42 (s, 1H), 5.80 (s, 1H), 6.90 (d, *J* = 8.0 Hz, 2H), 7.29 (d, *J* = 8.0 Hz, 2H), 10.69 (br, 1H); ^13^C-NMR δ 8.89, 10.3, 28.6, 28.98, 29.0, 41.9, 48.9, 55.4, 74.3, 80.1, 103.3, 114.3, 119.3, 119.9, 126.8, 128.2, 129.6, 131.7, 159.8, 160.8, 161.1, 181.5; ESI-MS obsd 439.2599, calcd. 439.2591 [(M + H)^+^, M = C_26_H_34_N_2_O_4_].

*1-[(Acetoxy)(4-methoxyphenyl)methyl]-9-tert-butoxycarbonyl-2,3-dihydro-3,3,7,8-tetramethyldipyrrin* (**6b-Ac**). Following a procedure [48] with slight modification, a solution of **6b** (20 mg, 46 µmol) in THF (1.8 mL) at room temperature was treated with acetic anhydride (9.3 mg, 92 µmol) and DMAP (11 mg, 92 µmol). The reaction mixture was stirred for 2 h. Saturated aqueous NaHCO_3_ (2 mL) was added, and the mixture was extracted with ethyl acetate. The organic extract was washed with water, dried (Na_2_SO_4_), and chromatographed (silica, ethyl acetate/hexanes (1:4)) to give a light brown oil (19 mg, 85%): ^1^H-NMR δ 1.14 (s, 3H), 1.17 (s, 3H), 1.57 (s, 9H), 2.02 (s, 3H), 2.25 (s, 6H), 2.41, 2.55 (AB, ^2^*J* = 18.0 Hz, 2H), 3.81 (s, 3H), 5.77 (s, 1H), 6.38 (s, 1H), 6.90 (dd, *J* = 7.2, 2.1 Hz, 2H), 7.37 (dd, *J* = 7.2, 2.1 Hz, 2H), 10.95 (br, 1H); ^13^C-NMR δ 8.9, 10.5, 21.1, 28.7, 28.88, 28.92, 41.2, 49.5, 55.4, 75.3, 79.8, 103.5, 114.3, 119.0, 120.4, 125.7, 127.7, 129.1, 130.1, 160.1, 161.2, 162.0, 170.4, 176.9; ESI-MS obsd 503.2517, calcd. 503.2516 [(M + Na)^+^, M = C_28_H_36_N_2_O_5_].

*8-Carbethoxy-7-ethyl-2,3-dihydro-1-[(hydroxy)(4-methylphenyl)methyl]-3,3-dimethyldipyrrin* (**8a**). Following a literature procedure ([55]; see Supporting Information therein) with slight modification, a solution of **7** (128 mg, 0.424 mmol) in THF (4.24 mL) was cooled to 0 °C and treated with *p*-tolylmagnesium bromide (1.48 mL, 1.48 mmol, 3.5 equiv, 1.0 M in THF) over the course of 5 min. After 10 min further, the ice bath was removed, and the reaction mixture was stirred for 2 h. The reaction mixture was cooled to 0 °C, whereupon saturated aqueous NH_4_Cl (15 mL) was added. The reaction mixture was extracted with ethyl acetate. The organic extract was washed with water, dried (Na_2_SO_4_), and chromatographed (silica, hexanes/ethyl acetate (3:1)) to give a pale yellow oil (75 mg, 45%): ^1^H-NMR δ 1.13 (s, 3H), 1.17 (t, *J* = 7.2 Hz, 3H), 1.19 (s, 3H), 1.34 (t, *J* = 7.2 Hz, 3H), 2.35 (s, 3H), 2.34, 2.45 (AB, *J* = 18.6 Hz, 2H), 2.82 (q, *J* = 7.2 Hz, 2H), 3.69–3.71 (br, 1H), 4.27 (q, *J* = 7.2 Hz, 2H), 5.44 (s, 1H), 5.82 (s, 1H), 7.19 (d, *J* = 8.4 Hz, 2H), 7.26 (d, *J* = 8.4 Hz, 2H), 7.42 (d, *J* = 3.0 Hz, 1H), 10. 72 (brs, 1H); ^13^C-NMR δ 14.5, 16.4, 18.0, 21.3, 29.0, 29.1, 41.6, 48.8, 59.3, 74.6, 103.4, 114.2, 125.1, 126.2, 126.8, 127.9, 129.6, 136.6, 138.4, 159.2, 165.5, 180.1; ESI-MS obsd 395.2328, calcd. 395.2329 [(M + H)^+^, M = C_24_H_30_N_2_O_3_]; λ_abs_ (CH_2_Cl_2_) 340 nm.

*8-Carbethoxy-7-ethyl-2,3-dihydro-1-(4-methylbenzoyl)-3,3-dimethyldipyrrin* (**8a-Ox**). A solution of **8a** in CDCl_3_ in an NMR tube changed from yellow to orange on standing for 7 days; chromatography (silica, hexanes/ethyl acetate (4:1)) gave a yellow oil: ^1^H-NMR δ 1.20 (t, *J* = 6.9 Hz, 3H), 1.31 (s, 6H), 1.35 (t, *J* = 7.2 Hz, 3H), 2.47 (s, 3H), 2.86 (q, *J* = 6.9 Hz, 2H), 2.98 (s, 2H), 4.27 (q, *J* = 7.2 Hz, 2H), 6.14 (s, 1H), 7.31 (d, *J* = 8.1 Hz, 2H), 7.38 (d, *J* = 3.6 Hz, 1H), 8.03 (d, *J* = 8.1 Hz, 2H), 10.65 (br, 1H); ESI-MS obsd 393.2168, calcd. 393.2173 [(M + H)^+^, M = C_24_H_28_N_2_O_3_]; λ_abs_ (CH_2_Cl_2_) 451 nm.

*1-[(Acetoxy)(4-methylphenyl)methyl]-8-carbethoxy-7-ethyl-2,3-dihydro-3,3-dimethyldipyrrin* (**8a-Ac**). Following a procedure [48] with slight modification, a solution of **8a** (70 mg, 0.18 mmol) in THF (7.2 mL) at room temperature was treated with acetic anhydride (38 mg, 0.36 mmol) and DMAP (44 mg, 0.361 mmol). The reaction mixture was stirred for 2 h. The reaction mixture was treated with saturated aqueous NaHCO_3_ (15 mL) and extracted with ethyl acetate. The organic extract was washed with water, dried (Na_2_SO_4_), and chromatographed [silica, hexanes/ethyl acetate (4:1)] to give a light brown oil (67 mg, 85%): ^1^H-NMR δ 1.16 (t, *J* = 7.2 Hz, 3H), 1.18 (s, 6H), 1.34 (t, *J* = 7.2 Hz, 3H), 2.19 (s, 3H), 2.37 (s, 3H), 2.45, 2.57 (AB, ^2^*J* =18.6 Hz, 2H), 2.80 (q, *J* = 7.2 Hz, 2H), 4.27 (q, *J* = 7.2 Hz, 2H), 5.82 (s, 1H), 6.49 (s, 1H), 7.20 (d, *J* = 8.4 Hz, 2H), 7.29 (d, *J* = 8.4 Hz, 2H), 7.37 (d, *J* = 3.0 Hz, 1H), 10.95 (brs, 1H); ^13^C-NMR δ 14.5, 16.4, 18.0, 21.0, 21.3, 28.9, 29.0, 41.0, 49.3, 59.2, 75.5, 103.8, 114.1, 124.9, 126.1, 127.3, 128.3, 129.6, 133.0, 138.9, 159.8, 165.4, 169.8, 175.5; ESI-MS obsd 437.2423, calcd. 437.2435 [(M + H)^+^, M = C_26_H_32_N_2_O_4_]; λ_abs_ (CH_2_Cl_2_) 343 nm.

*8-Carbethoxy-7-ethyl-2,3-dihydro-1-[(hydroxy)(4-methoxyphenyl)methyl]-3,3-dimethyldipyrrin* (**8b**). Following a literature procedure ([55]; see Supporting Information therein) with slight modification, a solution of **7** (52 mg, 0.17 mmol) in THF (1.1 mL) at 0 °C was treated with *p*-anisylmagnesium bromide (0.60 mL, 0.60 mmol, 3.5 equiv, 1.0 M in THF) over the course of 5 min. After 10 min further, the ice bath was removed, and the reaction mixture was stirred for 2 h. The reaction mixture was then cooled to 0 °C, whereupon saturated aqueous NH_4_Cl (4.0 mL) was added. The reaction mixture was extracted with ethyl acetate. The organic extract was washed with water, dried (Na_2_SO_4_), and chromatographed (silica, hexanes/ethyl acetate (3:2)) to give a red oil (33 mg, 47%): ^1^H-NMR δ 1.13 (s, 3H), 1.17 (t, *J* = 7.2 Hz, 3H), 1.20 (s, 3H), 1.35 (t, *J* = 7.2 Hz, 3H), 2.34, 2.46 (AB, *J* = 18.6 Hz, 2H), 2.82 (q, *J* = 7.2 Hz, 2H), 3.71 (br, 1H), 3.81 (s, 3H), 4.28 (q, *J* = 7.2 Hz, 2H), 5.43 (s, 1H), 5.82 (s, 1H), 6.90 (d, *J* = 8.7 Hz, 2H), 7.29 (d, *J* = 8.7 Hz, 2H), 7.43 (d, *J* = 3.0 Hz, 1H), 10.71 (br, 1H); ESI-MS obsd 411.2271, calcd. 411.2278 [(M + H)^+^, M = C_24_H_30_N_2_O_4_].

*1-[(Acetoxy)(4-methoxyphenyl)methyl]-8-carbethoxy-7-ethyl-2,3-dihydro-3,3-dimethyldipyrrin* (**8b-Ac**). Following a procedure [48] with slight modification, a solution of **8b** (20 mg, 49 µmol) in THF (1.9 mL) at room temperature was treated with acetic anhydride (9.5 µL, 98 µmol) and DMAP (12 mg, 98 µmol). The reaction mixture was stirred for 2 h. The reaction mixture was treated with saturated aqueous NaHCO_3_ (10 mL) and extracted with ethyl acetate. The organic extract was washed with water, dried (Na_2_SO_4_), and chromatographed (silica, hexanes/ethyl acetate (4:1)) to give a light brown oil (19 mg, 86%): ^1^H-NMR δ 1.13–1.18 (m, 9H), 1.34 (t, *J* = 6.9 Hz, 3H), 2.19 (s, 3H), 2.45, 2.57 (AB, ^2^*J* = 18.6 Hz, 2H), 2.80 (q, *J* = 7.2 Hz, 2H), 3.82 (s, 3H), 4.27 (q, *J* = 6.9 Hz, 2H), 5.82 (s, 1H), 6.47 (s, 1H), 6.92 (d, *J* = 8.2 Hz, 2H), 7.33 (d, *J* = 8.2 Hz, 2H), 7.38 (d, *J* = 3.0 Hz, 1H), 10.95 (br, 1H); ^13^C-NMR δ 14.5, 16.4, 18.0, 21.1, 28.9, 29.0, 41.1, 49.4, 55.4, 59.3, 75.2, 103.8, 114.1, 114.3, 124.9, 126.1, 127.9, 128.3, 128.9, 159.8, 160.1, 165.5, 170.0, 175.6; ESI-MS obsd 475.2203, calcd. 475.2203 [(M + Na)^+^, M = C_26_H_32_N_2_O_5_]; λ_abs_ (CH_2_Cl_2_) 343 nm.

*(E)-Ethyl 5-[(4,4-dimethyl-5-oxodihydrofuran-2(3H)-ylidene)(phenyl)methyl]-1H-pyrrole-3-carboxylate* (**11**). Following a literature procedure [19], in a Schlenk flask, a solution of **9** (2.5 g, 10 mmol) in dry acetonitrile (16.5 mL) was treated with **10** (2.1 g, 8.2 mmol), BnNEt_3_Cl (2.3 g, 8.2 mmol), and Et_3_N (10 mL). The mixture was deaerated by three freeze–pump–thaw cycles. A sample of Pd(PPh_3_)_4_ (0.47 g, 0.41 mmol) was then added, and the resulting mixture was further deaerated. The reaction mixture was heated at 80 °C for 16 h, and upon allowing to cool to room temperature, CH_2_Cl_2_ and water were added. The organic layer was dried (Na_2_SO_4_), concentrated, and chromatographed (silica, hexanes/ethyl acetate (7:3)) to afford a light yellow solid (1.9 g, 68%): m.p. 115–116 °C; ^1^H-NMR δ 1.35 (t, *J* = 6.9 Hz, 3H), 1.37 (s, 6H), 3.06 (s, 2H), 4.29 (q, *J* = 6.9 Hz, 2H), 6.56–6.57 (m, 1H), 7.27–7.37 (m, 6H), 8.17 (brs, 1H); ^13^C-NMR δ 14.5, 25.1, 39.9, 41.8, 59.9, 109.1, 111.0, 117.3, 123.3, 127.9, 128.6, 129.5, 129.7, 135.1, 145.1, 164.9, 179.6; ESI-MS obsd 340.1548, calcd. 340.1543 [(M + H)^+^, M = C_20_H_21_NO_4_].

*(E)-Ethyl 5-[(4,4-dimethyl-5-methylenedihydrofuran-2(3H)-ylidene)(phenyl)methyl]-1H-pyrrole-3-carboxy-late* (**12**). Following a general procedure [19], a solution of TiCp_2_Cl_2_ (6.2 g, 25 mmol) in toluene (100 mL) was treated dropwise with a solution of MeLi (1.6 M, 32 mL in Et_2_O, 50 mmol) at 0 °C under argon. The resulting mixture was stirred at 0 °C for 1 h, whereupon saturated aqueous NH_4_Cl solution was added. The organic layer was washed (water and brine), dried (Na_2_SO_4_) and filtered. The filtrate was treated with lactone **11** (1.7 g, 5.0 mmol) and TiCp_2_Cl_2_ (50 mg) in a Schlenk flask under argon. The resulting solution was heated at 80 °C for 16 h. The resulting mixture was allowed to cool to room temperature whereupon MeOH (1.8 mL), NaHCO_3_ (50 mg) and water (1.0 mL) were added. The resulting mixture was kept at 40 °C for 2 h with stirring and then filtered through Celite. The filtrate was concentrated and chromatographed (silica, hexanes/ethyl acetate (4:1)) to afford a gummy oil (1.1 g, 67%): ^1^H-NMR δ 1.27 (s, 6H), 1.34 (t, *J* = 7.2 Hz, 3H), 2.76 (s, 2H), 4.02 (d, *J* = 2.4 Hz, 1H), 4.24 (q, *J* = 7.2 Hz, 2H), 4.37 (d, *J* = 2.4 Hz, 1H), 6.46–6.48 (m, 1H), 7.21–7.36 (m, 6H), 8.53 (brs, 1H); ^13^C-NMR δ 14.5, 27.5, 39.8, 43.9, 59.8, 81.3, 105.7, 108.7, 116.5, 123.0, 126.8, 128.3, 129.6, 131.5, 136.8, 152.0, 165.3, 169.8; ESI-MS obsd 338.1756, calcd. 338.1751 [(M + H)^+^, M = C_21_H_23_NO_3_].

*8-Carbethoxy-2,3-dihydro-1,2,2-trimethyl-5-phenyldipyrrin* (**13**). Following a general procedure [19], a solution of **12** (544 mg, 1.60 mmol) in DMF (26.0 mL) was treated with 10% aqueous HCl (2.0 mL). After 30 min, NH_4_OAc (2.46 g, 32 mmol) and Et_3_N (4.5 mL, 32 mmol) were added, and the resulting mixture was stirred at 55 °C for 16 h. Then, the reaction mixture was treated with saturated aqueous KH_2_PO_4_ solution. Ethyl acetate (100 mL) was added, and the organic layer was washed (water), dried (Na_2_SO_4_), concentrated and chromatographed (silica, hexanes/ethyl acetate (4:1)) to afford a yellow solid (401 mg, 74%): m.p. 165–167 °C; ^1^H-NMR δ 1.13 (s, 6H), 1.28 (t, *J* = 6.9 Hz, 3H), 2.17 (s, 3H), 2.36 (s, 2H), 4.22 (q, *J* = 6.9 Hz, 2H), 6.01 (d, *J* = 2.4 Hz, 1H), 7.28–7.49 (m, 6H), 11.83 (brs, 1H); ^13^C-NMR δ 14.5, 15.7, 25.7, 43.9, 48.0, 59.5, 109.4, 116.0, 120.3, 124.2, 127.2, 128.4, 129.7, 134.7, 139.0, 147.9, 165.4, 186.9; ESI-MS obsd 337.1912, calcd. 337.1911 [(M + H)^+^, M = C_21_H_24_N_2_O_2_]; λ_abs_ (CH_2_Cl_2_) 330 nm.

*8-Carbethoxy-1-formyl-2,3-dihydro-2,2-dimethyl-5-phenyldipyrrin* (**14**). Following a general procedure [19], a solution of **13** (0.35 g, 1.0 mmol) in 1,4-dioxane (20 mL) was treated with SeO_2_ (0.33 g, 3.0 mmol) and stirred at room temperature for 60 min. Ethyl acetate and saturated NaHCO_3_ were then added, and the organic layer was washed (brine), dried, concentrated and chromatographed (silica, hexanes/ethyl acetate (4:1)) to afford a yellow solid (234 mg, 65%): m.p. 157–159 °C; ^1^H-NMR δ 1.29 (t, *J* = 7.2 Hz, 3H), 1.31 (s, 6H), 2.49 (s, 2H), 4.23 (q, *J* = 7.2 Hz, 2H), 6.23 (s, 1H), 7.29–7.60 (m, 6H), 10.0 (s, 1H), 11.42 (brs, 1H); ^13^C-NMR δ 14.5, 25.7, 45.8, 46.1, 59.9, 113.9, 117.0, 126.7, 128.1, 128.7, 129.1, 130.3, 133.8, 137.9, 148.4, 164.8, 177.0, 190.0; ESI-MS obsd 351.1701 calcd. 351.1703 [(M + H)^+^, M = C_21_H_22_N_2_O_3_]; λ_abs_ (CH_2_Cl_2_) 438 nm.

*8-Carbethoxy-1-[(hydroxy)(4-methylphenyl)methyl]-2,3-dihydro-2,2-dimethyl-5-phenyldipyrrin* (**15**). Following a literature procedure ([55]; see Supporting Information therein) with slight modification, a solution of **14** (97 mg, 0.29 mmol) in THF (1.9 mL) at 0 °C was treated with *p*-tolylmagnesium bromide (1.0 mL, 1.0 mmol, 3.5 equiv, 1.0 M in THF) over the course of 5 min. After 5 min further, the ice bath was removed, and the mixture was stirred for 2 h. The reaction mixture was then cooled to 0 °C whereupon saturated aqueous NH_4_Cl (10.0 mL) was added. The mixture was then extracted with ethyl acetate. The organic extract was washed with water, dried (Na_2_SO_4_), and chromatographed (silica, hexanes/ethyl acetate (3:1)) to give a light yellow solid (72 mg, 56%): m.p. 146–148 °C; ^1^H-NMR δ 0.75 (s, 3H), 1.24 (s, 3H), 1.29 (t, *J* = 6.9 Hz, 3H), 2.37 (s, 3H), 2.34, 2.46 (AB, ^2^*J* = 17.1 Hz, 2H), 3.49 (d, *J* = 4.8 Hz, 1H), 4.22 (q, *J* = 6.9 Hz, 2H), 5.52 (d, *J* = 4.8 Hz, 1H), 6.06 (s, 1H), 7.19–7.46 (m, 10H), 11.25 (brs, 1H); ^13^C-NMR δ 14.6, 21.3, 26.2, 26.5, 46.1, 47.6, 59.7, 72.0, 110.6, 116.2, 122.5, 124.8, 127.5, 127.6, 128.5, 129.6, 134.1, 137.2, 138.6, 138.7, 146.1, 165.3, 188.5; ESI-MS obsd 443.2341, calcd. 443.2329 [(M + H)^+^, M = C_28_H_30_N_2_O_3_]; λ_abs_ (CH_2_Cl_2_) 343 nm.

*1-[(Acetoxy)(4-methylphenyl)methyl]-8-carbethoxy-2,3-dihydro-2,2-dimethyl-5-phenyldipyrrin* (**15-Ac**). Following a procedure [48] with slight modification, a solution of **15** (22 mg, 52 µmol) in THF (2.1 mL) at room temperature was treated with acetic anhydride (11 mg, 0.11 mmol) and DMAP (13 mg, 0.11 mmol). The reaction mixture was stirred for 2 h, and then treated with saturated aqueous NaHCO_3_ (5 mL). The reaction mixture was extracted with ethyl acetate. The organic extract was washed with water, dried (Na_2_SO_4_), and chromatographed (silica, hexanes/ethyl acetate (4:1)) to give a light yellow solid (22 mg, 92%): m.p. 65–67 °C; ^1^H-NMR δ 0.91 (s, 3H), 1.28 (s, 3H), 1.29 (t, *J* = 6.9 Hz, 3H), 2.18 (s, 3H), 2.39 (s, 3H), 2.35, 2.45 (AB, ^2^*J* = 16.8 Hz, 2H), 4.22 (q, *J* = 6.9 Hz, 2H), 6.06 (d, *J* = 1.8 Hz, 1H), 6.53 (s, 1H), 7.23–7.43 (m, 10H), 11.58 (brs, 1H); ^13^C-NMR δ 14.5, 21.1, 21.3, 26.2, 26.3, 45.3, 48.3, 59.6, 72.6, 110.4, 116.1, 123.0, 124.6, 127.5, 128.3, 128.5, 129.5, 133.4, 134.4, 138.4, 139.2, 146.8, 165.2, 169.9, 183.8; ESI-MS obsd 485.2445, calcd. 485.2435 [(M + H)^+^, M = C_30_H_32_N_2_O_4_]; λ_abs_ (CH_2_Cl_2_) 343 nm.

*2-Formyl-3-(4-methylphenyl)-N-p-tosylpyrrole* (**17**). Following a reported procedure [42], a mixture of NaH (1.17 g, 60 wt % in oil, 29 mmol) in THF (90 mL) was treated portionwise with pyrrole **16** (4.20 g, 22.7 mmol) at 0 °C under argon. The reaction mixture was stirred for 20 min at 0 °C, whereupon *p*-tosyl chloride (6.50 g, 34.1 mmol) was added. The resulting heterogeneous mixture was stirred for 3 h at room temperature. Water and CH_2_Cl_2_ were added. The organic phase was washed (brine), dried (Na_2_SO_4_) and concentrated to a pale brown solid. Column chromatography (silica, hexanes/CH_2_Cl_2_ (1:3)) afforded a pale grey solid (6.34 g, 82%): m.p. 178–180 °C; ^1^H-NMR (400 MHz) δ 2.38 (s, 3H), 2.43 (s, 3H), 6.47 (d, *J* = 3.3 Hz, 1H), 7.21 (d, *J* = 7.9 Hz, 2H), 7.30 (d, *J* = 7.9 Hz, 2H), 7.33 (d, *J* = 8.2 Hz, 2H), 7.82–7.83 (m, 1H), 7.94 (d, *J* = 8.2 Hz, 2H), 9.62 (s, 1H); ^13^C-NMR (100 MHz) δ 21.5, 21.9, 113.1, 128.1, 128.6, 129.5, 129.6, 129.7, 129.8, 129.9, 135.4, 139.0, 144.0, 145.6, 178.5; ESI-MS obsd 340.1007, calcd. 340.1002 [(M + H)^+^, M = C_19_H_17_NO_3_S].

*3-(4-Methylphenyl)-2-(2-nitroethyl)-N-p-tosylpyrrole* (**18**). Following a reported procedure [43], a mixture of **17** (6.30 g, 18.6 mmol), potassium acetate (2.00 g, 20.4 mmol), methylamine hydrochloride (1.51 g, 22.3 mmol), nitromethane (2.50 mL, 46.6 mmol) and acetic acid (106 µL, 1.86 mmol) in ethanol (6.5 mL) was sonicated (benchtop sonication bath) for 10 min, and then stirred at room temperature for 42 h. Water was added. The resulting yellow precipitate was washed (water, cold ethanol), and then dried under high vacuum for 4 h. The crude product was dissolved in CHCl_3_/2-propanol (133 mL, 3:1), treated with silica (22.3 g), and vigorously stirred upon addition of NaBH_4_ (1.41 g, 37.1 mmol). The mixture was vigorously stirred for 2 h at room temperature. The progress of the reaction was monitored by ^1^H-NMR spectroscopy. The reaction mixture was filtered. The filtrate was washed (water, brine), dried (Na_2_SO_4_), and concentrated to a brown oil. The oil was chromatographed (silica, hexanes/ethyl acetate (3:1)). The product was recrystallized from hot EtOH and cooled overnight at 1 °C to afford a white solid (3.45 g, 48%): m.p. 115–117 °C; ^1^H-NMR δ 2.37 (s, 3H), 2.44 (s, 3H), 3.46 (t, *J* = 8.0 Hz, 2H), 4.55 (t, *J* = 8.0 Hz, 2H), 6.35–6.37 (m, 1H), 7.11 (d, *J* = 7.9 Hz, 2H), 7.18 (d, *J* = 7.9 Hz, 2H), 7.33 (d, *J* = 8.1 Hz, 2H), 7.37–7.39 (m, 1H), 7.69 (d, *J* = 8.1 Hz, 2H); ^13^C-NMR (100 MHz) δ 21.4, 21.9, 24.0, 74.3, 113.9, 123.4, 126.9, 128.4, 129.7, 130.5, 130.9, 131.2, 136.1, 137.6, 145.7; Anal. Calcd. for C_20_H_20_N_2_O_4_S: C, 62.48; H, 5.24; N, 7.29; S, 8.34. Found: C, 62.40; H, 5.10; N, 7.21; S, 8.24.

*1,1-Dimethoxy-4,4-dimethyl-6-[3-(4-methylphenyl)-N-p-tosylpyrrol-2-yl]-5-nitro-2-hexanone* (**20**). A mixture of **18** (3.42 g, 8.90 mmol) and **19** (3.19 g, 20.2 mmol) was treated with DBU (5.20 mL, 26.7 mmol) at room temperature. The reaction mixture was stirred for 24 h at room temperature. Saturated aqueous NH_4_Cl was added, and the mixture was extracted with ethyl acetate. The organic phase was washed (water, brine), dried (Na_2_SO_4_), and concentrated to a brown oil. A first chromatography (silica, hexanes/ethyl acetate (1:3)) and a second chromatography (silica, CH_2_Cl_2_) afforded a slightly yellow oil (1.02 g, 21%): ^1^H-NMR δ 1.05 (s, 3H), 1.12 (s, 3H), 2.37 (s, 3H), 2.43 (s, 3H), 2.49, 2.59 (AB, *J* = 18.4 Hz, 2H), 3.28 (ABX, *J* = 2.9 Hz, *J* = 15.3 Hz, 1H), 3.34 (s, 3H), 3.38 (s, 3H), 3.62 (ABX, *J* = 10.9 Hz, *J* = 15.3 Hz, 1H), 4.31 (s, 1H), 5.10 (ABX, *J* = 2.9 Hz, *J* = 10.9 Hz, 1H), 6.26 (d, *J* = 3.3 Hz, 1H), 7.07 (d, *J* = 7.8 Hz, 2H), 7.16 (d, *J* = 7.8 Hz, 2H), 7.30–7.34 (m, 3H), 7.65 (d, *J* = 8.5 Hz, 2H); ^13^C-NMR (100 MHz) δ 21.4, 21.9, 23.6, 23.9, 25.3, 29.9, 36.6, 44.3, 55.0, 94.3, 104.5, 114.9, 124.2, 124.6, 126.6, 128.9, 129.3, 130.3, 131.5, 132.2, 136.3, 137.2, 145.4, 203.2; ESI-MS obsd 565.1994, calcd. 565.1979 [(M + Na)^+^, M = C_28_H_34_N_2_O_7_S].

*2,3,4,5-Tetrahydro-1-(dimethoxymethyl)-3,3-dimethyl-7-(4-methylphenyl)-N^11^-p-tosyldipyrrin N^10^-oxide* (**21**). Following a general procedure [42], a solution of **20** (1.02 g, 1.88 mmol) in AcOH/EtOH (19 mL, 1:1) was treated with zinc dust (3.09 g, 47.6 mmol) at 0 °C. The reaction mixture was vigorously stirred for 1 h at 0 °C, then diluted with ethyl acetate and filtered through Celite. The filtrate was concentrated. The resulting oil was purified by column chromatography (silica, hexanes/ethyl acetate (2:3)) to afford a white solid (400 mg, 42%): m.p. 160–163 °C; ^1^H-NMR (400 MHz) δ 0.57 (s, 3H), 0.78 (s, 3H), 2.32 (s, 2H), 2.34 (s, 3H), 2.41 (s, 3H), 3.23 (ABX, *J* = 11.0 Hz, *J* = 15.3 Hz, 1H), 3.40 (s, 3H), 3.43 (s, 3H), 3.70 (ABX, *J* = 4.0 Hz, *J* = 15.3 Hz, 1H), 4.33–4.37 (m, 1H), 5.43 (s, 1H), 6.33 (d, *J* = 3.3 Hz, 1H), 7.11–7.15 (m, 4H), 7.27 (d, *J* = 8.4 Hz, 2H), 7.38 (d, *J* = 3.3 Hz, 1H), 7.75 (d, *J* = 8.4 Hz, 2H); ^13^C-NMR (100 MHz) δ 21.4, 21.9, 22.6, 23.0, 26.7, 38.1, 41.9, 55.3, 55.8, 79.9, 97.9, 114.6, 124.1, 125.9, 127.1, 128.9, 129.6, 130.3, 131.0, 132.2, 137.2, 145.3. Anal. Calcd. for C_28_H_34_N_2_O_5_S: C, 65.86; H, 6.71; N, 5.49. Found: C, 65.94; H, 6.70; N, 5.38.

*2,3,4,5-Tetrahydro-1-(dimethoxymethyl)-3,3-dimethyl-7-(4-methylphenyl)-N^11^-p-tosyldipyrrin* (**22**). Following a general procedure [42], TiCl_4_ (5.35 mL, 5.35 mmol, 1.0 M in CH_2_Cl_2_) was slowly added with stirring to THF (15.3 mL) under argon at 0 °C. The resulting yellow solution was slowly treated with LiAlH_4_ (3.82 mL, 3.82 mmol, 1.0 M in THF). The resulting black mixture was stirred at room temperature for 15 min. TEA (4.78 mL, 34.4 mmol) was added. The resulting black mixture was stirred for 2 min at room temperature. The black mixture was slowly poured into a solution of **21** (378 mg, 0.740 mmol) in THF (12.7 mL) at 0 °C. The mixture was stirred for 1 h at room temperature. Water and ethyl acetate were added. The organic extract was washed (brine), dried (Na_2_SO_4_), concentrated and chromatographed (silica, CH_2_Cl_2_/ethyl acetate (5:1)) to afford a white solid (300 mg, 82%): m.p. 105–107 °C; ^1^H-NMR (400 MHz) δ 0.79 (s, 3H), 1.05 (s, 3H), 2.32 (s, 3H), 2.35 (s, 2H), 2.40 (s, 3H), 2.94–2.98 (m, 2H), 3.30 (s, 3H), 3.35 (s, 3H), 4.07–4.12 (m, 1H), 4.65 (s, 1H), 6.36 (d, *J* = 3.3 Hz, 1H), 7.09 (d, *J* = 8.1 Hz, 2H), 7.27 (d, *J* = 8.1 Hz, 2H), 7.32–7.36 (m, 3H), 7.64 (d, *J* = 8.1 Hz, 2H); ^13^C-NMR (100 MHz) δ 21.6, 22.1, 23.3, 27.1, 27.4, 42.0, 49.1, 54.9, 55.2, 79.7, 94.8, 103.7, 115.0, 123.6, 127.0, 129.3, 129.4, 130.5, 133.1, 136.7, 137.2, 145.2, 173.6; ESI-MS obsd 495.2307, calcd. 495.2313 [(M + H)^+^, M = C_28_H_34_N_2_O_4_S].

*2,3,8,8,12,13,18,18-Octamethyl-5,15-bis(4-methylphenyl)bacteriochlorin* (**B1-T_2_**). A solution of **6a-Ac** (32.0 mg, 0.0689 mmol) in CH_3_CN (3.83 mL) was treated with BF_3_·O(Et)_2_ (67.9 µL, 0.536 mmol). The reaction mixture was heated to 80 °C for 2 h in a round-bottomed flask (air atmosphere) fitted with a rubber stopper, which itself was pierced with a syringe needle. The reaction mixture was allowed to cool to room temperature and then treated with TEA (89.6 µL, 0.643 mmol). The resulting mixture was diluted with ethyl acetate (~15 mL), washed with water, dried (Na_2_SO_4_), and concentrated. Column chromatography (silica, hexanes/CH_2_Cl_2_ (1:1)) afforded a greenish solid (3.8 mg). Trituration with CH_2_Cl_2_/hexanes gave a green solid (3.3 mg, 16%): ^1^H-NMR δ −1.64–1.58 (brs, 2H), 1.85 (s, 12H), 2.35 (s, 6H), 2.63 (s, 6H), 3.24 (s, 6H), 3.93 (s, 4H), 7.46 (d, *J* = 8.0 Hz, 4H), 7.65 (d, *J* = 8.0 Hz, 4H), 8.59 (s, 2H); MALDI-MS obsd 607.7; ESI-MS obsd 606.3714, calcd. 606.3717 [(M)^+^, M = C_42_H_46_N_4_]; λ_abs_(toluene) 356, 382, 504, 726 nm.

*5,15-Bis(4-methoxyphenyl)-2,3,8,8,12,13,18,18-octamethylbacteriochlorin* (**B1-A_2_**). A solution of **6b-Ac** (2.2 mg, 4.6 µmol) in CH_3_CN (0.25 mL) was treated with BF_3_·O(Et)_2_ (4.4 µL, 36 µmol) in a glass vial (air atmosphere) fitted with a rubber stopper, which itself was pierced with a syringe needle. The reaction mixture was stirred at room temperature for 24 h. The yield of bacteriochlorin was 41% upon absorption spectroscopic examination. (The weight of the vial was checked to confirm the absence of any significant solvent loss, the occurrence of which would inflate the spectroscopic yield determination; no correction was required.) The reaction mixture was treated with TEA (11 µL, 72 µmol). The resulting mixture was diluted with ethyl acetate (5 mL) and washed with water, dried (Na_2_SO_4_), and concentrated. Silica chromatography afforded a greenish brown solid, which exhibited limited solubility in diverse organic solvents (hexanes, ethyl acetate, dichloromethane, methanol): MALDI-MS obsd 639.4, calcd. 638.36 (C_42_H_46_N_4_O_2_); λ_abs_ (CH_2_Cl_2_) 355, 382, 503, 726 nm.

*3,13-Dicarbethoxy-2,12-diethyl-8,8,18,18-tetramethyl-5,15-bis(4-methylphenyl)bacteriochlorin* (**B2-T_2_**). A solution of **8a-Ac** (31 mg, 71 µmol) in CH_3_CN (3.9 mL) was treated with BF_3_·O(Et)_2_ (68 µL, 0.55 mmol) for 4 h following the procedure for **A_2_-BC1**. The reaction mixture was treated with TEA (77 µL, 0.55 mmol) and diluted with ethyl acetate (20 mL), and then washed with water, dried (Na_2_SO_4_), and concentrated. Column chromatography (silica, hexanes/ethyl acetate (4:1)) afforded a green solid (3.1 mg, 12%): ^1^H-NMR δ −1.28 (br, 2H), 1.29 (t, *J* = 7.2 Hz, 6H), 1.67 (t, *J* = 7.2 Hz, 6H), 1.83 (s, 12H), 2.59 (s, 6H), 3.78 (q, *J* = 7.2 Hz, 4H), 3.87 (q, *J* = 7.2 Hz, 4H), 3.95 (s, 4H), 7.44 (d, *J* = 7.5 Hz, 4H), 7.71 (d, *J* = 7.5 Hz, 4H), 8.62 (s, 2H); ^13^C-NMR δ 14.2, 17.7, 20.1, 21.6, 31.2, 45.5, 51.6, 61.2, 94.5, 113.5, 125.6, 128.3, 131.9, 132.0, 133.0, 137.1, 137.5, 138.7, 159.9, 168.1, 169.0; MALDI-MS obsd 751.3; ESI-MS obsd 751.41805, calcd. 751.42178 [(M + H)^+^, M = C_48_H_54_N_4_O_4_]; λ_abs_ (toluene) 358, 382, 516, 739 nm.

*3,13-Dicarbethoxy-2,12-diethyl-5,15-bis(4-methoxyphenyl)-8,8,18,18-tetramethylbacteriochlorin* (**B2-A_2_**). A solution of **8b-Ac** (14 mg, 31 µmol) in CH_3_CN (1.7 mL) was treated with BF_3_·O(Et)_2_ (30 µL, 0.24 mmol) for 2 h following the procedure for **A_2_-BC1**. The reaction mixture was treated with TEA (33 µL, 0.24 mmol). The resulting mixture was diluted with ethyl acetate (20 mL) and then washed with water, dried (Na_2_SO_4_), and concentrated. Column chromatography (silica, CH_2_Cl_2_) afforded a green solid (1.9 mg, 16%): ^1^H-NMR δ −1.31 (br, 2H), 1.30 (t, *J* = 7.5 Hz, 6H), 1.67 (t, *J* = 7.5 Hz, 6H), 1.84 (s, 12H), 3.77 (br, 4H), 3.95 (q, *J* = 7.5 Hz, 8H), 4.01 (s, 6H), 7.16 (d, *J* = 8.7 Hz, 4H), 7.73 (d, *J* = 8.7 Hz, 4H), 8.62 (s, 2H); MALDI–MS obsd 783.8; ESI-MS obsd 783.41449, calcd. 783.41161 [(M + H)^+^, M = C_48_H_54_N_4_O_6_]; λ_abs_(toluene) 358, 382, 517, 739 nm. In this reaction one additional bacteriochlorin was observed in trace quantities upon chromatography (following the title compound), although not isolated in pure form (LD-MS *m/z* = 739.1; λ_abs_ = 382, 549, 759 nm).

*3,13-Dicarbethoxy-8,8,18,18-tetramethyl-5,15-bis(4-methylphenyl)-10,20-diphenylbacteriochlorin* (**B3-P_2_T_2_**). A solution of **15** (10 mg, 23 µmol) in CH_2_Cl_2_ (1.3 mL) was treated with trifluoroacetic anhydride (16 µL, 0.12 mmol) in a glass vial. The reaction flask was sealed with a rubber stopper and heated at 40 °C for 24 h. Upon cooling to room temperature, the reaction mixture was treated with TEA (33 µL, 0.24 mmol). The resulting mixture was concentrated and chromatographed (silica, CH_2_Cl_2_)) to afford a pink solid (1.3 mg, 13%): ^1^H-NMR δ −0.46 (br, 2H), 1.18 (t, *J* = 7.2 Hz, 6H), 1.30 (s, 12H), 2.54 (s, 6H), 3.67 (s, 4H), 3.77 (q, *J* = 7.2 Hz, 4H), 7.30 (d, *J* = 8.4 Hz, 4H), 7.59–7.61 (m, 7H), 7.71–7.73 (m. 7H), 8.03 (d, *J* = 2.1 Hz, 2H); MALDI-MS obsd 847.6; ESI-MS obsd 847.41996, calcd. 847.42178 [(M + H)^+^, M = C_56_H_54_N_4_O_4_]; λ_abs_(toluene) 382, 533, 743 nm.

### 4.4. Plotting Absorption Spectra

A major challenge in the evaluation of the absorption spectra of the bacteriochlorins is how to normalize the spectra for comparison. In general, the absorption spectra of porphyrinic macrocycles (porphyrins, chlorins, bacteriochlorins) are normalized on the basis of their intensities (at either the B or Q band), which can be misleading for many bacteriochlorins due to the splitting/merging of the B_x_ and B_y_ bands (in some cases more than two). To overcome such shortcomings, the integrated area of the Q_y_ bands relative to the total integrated full spectrum (ΣT) based on wavenumbers (cm^−1^) provides a better gauge of any relative hyperchromic/hypochromic change of the Q_y_ bands [36,53]. In displays such as Figure 5, the integrated area is assessed on an energy basis (i.e., wavenumber (cm^−1^)) while the *x*-axis display is in the more conventional wavelength (nm); accordingly, the shorter wavelength bands (the B bands) have stronger impact compared to the longer wavelength bands (the Q bands).

## 5. Conclusions

A new synthetic route to *meso*-arylbacteriochlorin has been developed that relies on the acid-catalyzed self-condensation of a dihydrodipyrrin–carbinol or dihydrodipyrrin–acetate followed by air oxidation. The E-W route affords *meso*-diarylbacteriochlorins whereas the N-S route provides access to meso-tetraarylbacteriochlorins. Comparison of the yield of a *meso*-unsubstituted bacteriochlorin from a tetrahydrodipyrrin–acetal (0%) versus a dihydrodipyrrin–acetal (41%) implies the importance of resonance stabilization, by the distant pyrrole, of the oxocarbenium ion in the dihydrodipyrrin structure. A hypsochromic shift of the Q_y_ band (~20 nm) occurs when *meso*-aryl and pyrrole-β-carbethoxy groups are juxtaposed. Further development of these approaches would benefit from more concise routes to the dihydrodipyrrin–carboxaldehyde, or alternative routes to install the aryl group(s) leading to the dihydrodipyrrin–acetate.

## Figures and Tables

**Table 1:** Absorption and Fluorescence Spectral Properties of *meso*-Arylbacteriochlorins ^a^.

Compound	λB_y_ abs (nm)	λB_x_ abs (nm)	λQ_x_ abs (nm)	λQ_y_ abs (nm)	λQ_y_ ems (nm)	Φ_f_^b^
**B1-T_2_**	356	382	503	726	729	0.12
**B2-T_2_**	357	381	516	739	747	0.15
**B3-P_2_T_2_**	362 ^c^	382	533	743	757	0.15

^a^ In toluene at room temperature; ^b^ Fluorescence quantum yield; ^c^ Shoulder.

**Table 2:** Absorption Spectral Properties of Bacteriochlorins *a*.

Compound	λB_y_ (Δ ^b^) (nm)	λB_x_ (Δ ^b^) (nm)	λQ_x_ (Δ ^b^) (nm)	λQ_y_ (Δ ^b^) (nm)	Q_y_ fwhm (Δ ^b^) (nm)	ΣQ_y_/ΣT ^c^
**iso-B2**	355	384	521	760	20	0.126
**iso-B2-T_2_**	362 (+7)	388 (+4)	535 (+14)	759 (−1)	25 (+5)	0.117
**B2 ^d^**	354	383	521	760	20	0.136
**B2-T_2_**	357 (+3)	381 (−2)	516 (−5)	739 (−21)	22 (+2)	0.106
**B1 ^e^**	346	374	490	721	12	0.107
**B1-T_2_**	356 (+10)	382 (+8)	503 (+13)	726 (+5)	14 (+2)	0.101
**B3**	352	379	523	754	16	0.133
**B3-T_2_**	361 ^f^	383	543 (+20)	759 (+5)	16 (0)	0.125
**B3-P_2_T_2_**	362 ^g^	382	533 (+10)	743 (−11)	27 (+11)	0.100
**B6 ^d^**	340	365	489	713	12	0.091
**TPBC ^h^**	355 (+15)	378 (+13)	522 (+33)	742 (+29)	15 (+3)	0.091

^a^ In toluene at room temperature; ^b^ Shift compared to the corresponding benchmark bacteriochlorin lacking *meso*-aryl substituents; ^c^ Ratio of the integrated intensities of the Q_y_ band (ΣQ_y_) versus the integrated intensity of the full spectrum (ΣT, 300–900 nm) for spectra plotted in wavenumber (cm^−1^); ^d^ [53]; ^e^ [36]; ^f^ 373 nm; ^g^ Shoulder; ^h^ [6].

**Table 3:** Fluorescence Spectral Properties of Bacteriochlorins ^a^.

Compound	λQ_y_ (Δ ^b^) (nm)	fwhm (Δ ^b^) (nm)	Δν (cm^−1^) ^c^	Φ_f_^d^
**iso-B2**	764	20	70	0.11
**iso-B2-T_2_**	766 (+2)	25 (+5)	120	0.15
**B2 ^e^**	764	21	60	0.14
**B2-T_2_**	747 (−17)	27 (+6)	140	0.15
**B1 ^f^**	723	14	40	0.10
**B1-T_2_**	729 (+6)	18 (+4)	60	0.12
**B3**	757	18	50	0.12
**B3-T_2_**	761 (+3)	18 (0)	35	0.16
**B3-P_2_T_2_**	757 (0)	34 (+16)	250	0.15

^a^ In toluene at room temperature; ^b^ Shift compared to the corresponding benchmark bacteriochlorin lacking *meso*-aryl substituents; ^c^ Stokes shift; ^d^ Fluorescence quantum yield; ^e^ [53]; ^f^ [36].

## Supplementary Materials

Supplementary materials are available online. ^1^H-NMR and ^13^C-NMR spectra of new compounds are available online.

## Appendix A

### Appendix A.1. Acid Survey for the Self-Condensation of ***6a*** and ***6a-Ac***

The self-condensation was carried out under a variety of acid-catalysis conditions, typically open to air, with monitoring of the reaction by absorption spectroscopy and LD-MS. The conditions employed were those used previously for dihydrodipyrrin–acetals, including (1) 140 mM BF_3_·O(Et)_2_ in CH_3_CN [18]; or (2) 72 mM TMSOTf/144 mM DTBP in CH_2_Cl_2_ [31]; or the conditions employed for dihydrodipyrrin–carboxaldehydes: neat TFA [36]. Thus, reaction of **6a** in CH_3_CN containing BF_3_·O(Et)_2_ at room temperature gave <1% yield (Table A1, entry 1), but ~4% yield upon reflux for several hours (entry 2).

The self-condensation of **6a-Ac** in the presence of TFA or TMSOTf/DTBP gave di-*p*-tolylbacteriochlorin **B1-T_2_** in at most ~1% yield (entries 3–5) and absorption spectra reminiscent of tetradehydrocorrin-type compounds, which were not further analyzed. The reaction in CH_3_CN containing BF_3_·O(Et)_2_ gave yields that varied depending on the concentration of BF_3_·O(Et)_2_ (entries 6–9), with the highest yield (17%) obtained with 140 mM BF_3_·O(Et)_2_ (entry 7). Reaction at lower concentration of **6a-Ac** (5 mM) and BF_3_·O(Et)_2_ (50 mM) gave only 4.7% yield (entry 10). Segueing from entry 7, the same conditions but at reflux gave a higher yield (22%, entry 11) in shorter reaction time (2 vs. 21 h). Nitroarenes are known to provide oxidative conversion of a porphyrinogen to a porphyrin [56], yet PhNO_2_ did not give any significant improvement in yield (entries 12–14). Attempts to use an atmosphere of O_2_ (instead of air) did not afford increased yields (entry 15) nor did the reaction in TFA with use of DDQ as oxidant (entry 16); indeed, the latter gave evidence by LD-MS for the presence of an oxobacteriochlorin product as well as uncharacterized species. Otherwise, a single bacteriochlorin (**B1-T_2_**) macrocycle was reliably observed by TLC and LD-MS analyses across the reaction survey.

**Table A1:** Survey of Conditions for the Self-condensation of Carbinol **6a** or Acetate **6a-Ac** ^a^.

Entry	Reactant (mM)	Acid (mM)	Solvent	Oxidant	Conditions	Yield (%) ^b^
1	**6a** [18]	BF_3_·O(Et)_2_ [140]	CH_3_CN	air	r.t., 16 h	<1
2	**6a** [18]	BF_3_·O(Et)_2_ [140]	CH_3_CN	air	reflux, 2 h	4.3
3	**6a-Ac** [18]	Neat TFA ^c^	–	air	r.t., 21 h	0.6
4	**6a-Ac** [18]	50% (*v*/*v*) TFA ^c^	CH_2_Cl_2_	air	r.t., 21 h	1.1
5	**6a-Ac** [18]	TMSOTf [72], DTBP [144]	CH_2_Cl_2_	air	r.t., 4 h ^d^	0.9
6	**6a-Ac** [18]	BF_3_·O(Et)_2_ [70]	CH_3_CN	air	r.t., 21 h	13
7	**6a-Ac** [18]	BF_3_·O(Et)_2_ [140]	CH_3_CN	air	r.t., 21 h	17
8	**6a-Ac** [18]	BF_3_·O(Et)_2_ [280]	CH_3_CN	air	r.t., 21 h	12
9	**6a-Ac** [18]	BF_3_·O(Et)_2_ [560]	CH_3_CN	air	r.t., 21 h	4.4
10	**6a-Ac** [5]	BF_3_·O(Et)_2_ [50]	CH_3_CN	air	r.t., 21 h	4.7
11	**6a-Ac** [18]	BF_3_·O(Et)_2_ [140]	CH_3_CN	air	reflux, 2 h	22
12	**6a-Ac** [18]	BF_3_·O(Et)_2_ [140]	CH_3_CN	air ^e^	r.t., 21 h	12
13	**6a-Ac** [18]	BF_3_·O(Et)_2_ [140]	CH_3_CN	air ^e^	reflux, 2 h	21
14	**6a-Ac** [18]	BF_3_·O(Et)_2_ [140]	PhNO_2_	air	reflux, 2 h	7.1
15	**6a-Ac** [18]	BF_3_·O(Et)_2_ [140]	CH_3_CN	O_2_	reflux, 2 h	8.6
16	**6a-Ac** [18]	Neat TFA	–	DDQ	r.t., 1 h	7.0 ^f^

^a^ Each reaction was carried out in a 4 mL vial containing a magnetic stir bar. In each reaction, 1.0 mg of reactant (**6a-Ac**) and 120 μL of solvent was used (entry 10 used 427 μL of solvent); ^b^ Yields were determined spectroscopically by the intensity of the Q_y_ band (~726 nm, assumed ε_Qy_ = 120,000 M^−1^cm^−1^ [18,19]) of crude samples. The crude sample was prepared by removal of a specific amount (5–10 μL) of sample from the reaction mixture and dilution with 3 mL of CH_2_Cl_2_; ^c^ The color of the reaction mixture changed quickly (in 1 h) to a strong greenish blue. The overall shape of the absorption spectrum was closer to that of tetradehydrocorrin-like compounds; ^d^ The reaction was monitored for 21 h. The intensity of the Q_y_ band was weak, broad and of uncertain magnitude; ^e^ Also contains PhNO_2_ (810 mM); ^f^ The quantity of DDQ employed was 3 equiv. The product was likely an oxobacteriochlorin.

### Appendix A.2. Acid Survey for the Self-Condensation of ***15-Ac***

The self-condensation of **15-Ac** was examined under a variety of conditions (Table A2). The use of BF_3_·O(Et)_2_ gave no bacteriochlorin (entries 1–4), and moreover, the crude samples gave data suggestive of tetradehydrocorrin-like macrocycles (upon examination by LD-MS and absorption spectroscopy). Bacteriochlorin formation was not observed under neat TFA, TMSOTf/DTBP or InCl_3_ (entries 5–8), even using 1,2-dichloroethane (DCE) or toluene as solvent at 80 °C. The self-condensation of dihydrodipyrrin–acetate **15-Ac** in the presence of Bi(OTf)_3_ gave bacteriochlorin in 0.8% yield (entry 9) and 1.2% yield with FeCl_3_/AgOTf (entry 10). Bacteriochlorin formation was observed in up to 2% yield with *p*-TsOH·H_2_O as the acid catalyst (entries 11 and 12).

**Table A2:** Survey of Conditions for the Self-condensation of Dihydrodipyrrin–acetate **15-Ac** ^a^.

Entry	Acid (mM)	Solvent	Oxidant	Conditions	Yield (%) *^b^*
1	BF_3_·O(Et)_2_ [140]	CH_3_CN	air	r.t., 24 h	– ^c^
2	BF_3_·O(Et)_2_ [140]	CH_3_CN	air	80 °C, 2 h	TDC ^d^
3	BF_3_·O(Et)_2_ [560]	CH_3_CN	air	80 °C, 10 min	TDC ^d^
4	Neat BF_3_·O(Et)_2_	–	air	r.t., 19 h	TDC ^d^
5	Neat TFA	–	air	r.t., 19 h	– ^e^
6	TMSOTf [72], DTBP [144]	CH_2_Cl_2_	air	r.t., 19 h	– ^e^
7	TMSOTf [72], DTBP [144]	DCE	air	80 °C, 19 h	– ^c^
8	InCl_3_ [140]	Toluene	air	80 °C, 2 h	– ^c^
9	Bi(OTf)_3_ [36]	CH_2_Cl_2_	air	r.t., 16 h	0.8
10	FeCl_3_ [9], AgOTf [18]	DCE	air	r.t., 16 h	1.2
11	*p*-TsOH·H_2_O [90]	AcOH	air	80 °C, 2 h	1.9
12	*p*-TsOH·H_2_O [360]	DCE	air	50 °C, 1 h	1.2

^a^ Each reaction was carried out in a 4 mL vial containing a magnetic stir bar. In each reaction, 2.0 mg of reactant (**15-Ac**, 18 mM) and 0.23 mL of solvent was used; ^b^ Yields were determined spectroscopically by the intensity of the Q_y_ band (~741 nm, ε_Qy_ = 120,000 M^−1^cm^−1^ [18,19]) of crude samples. The crude sample was prepared by removal of a specific amount (5 μL) of sample from the reaction mixture and dilution with 3 mL of CH_2_Cl_2_; ^c^ No bacteriochlorin was formed; ^d^ The absorption spectrum was suggestive of tetradehydrocorrin (TDC)-like compounds; *^e^* TLC analysis shows the starting material **15-Ac** and no bacteriochlorin.

### Appendix A.3. Acid Survey for the Self-Condensation of ***15***

The self-condensation of dihydrodipyrrin–carbinol **15** was examined under a variety of conditions (Table A3). The formation of bacteriochlorin was not observed under TFA (neat) at room temperature or 50 °C for 24 h (entry 1).

**Table A3:** Survey of Conditions for the Self-condensation of Dihydrodipyrrin–carbinol 15 ^a^.

Entry	Reactant (mM)	Acid (mM)	Solvent	Oxidant	Conditions	Yield (%) ^b^
1	**15** [18]	Neat TFA	–	air	50 °C, 24 h	-- ^c^
2	**15** [18]	TFAA [45]	CH_2_Cl_2_	air	r.t., 16 h	1.8
3	**15** [18]	TFAA [90]	CH_2_Cl_2_	air	r.t., 24 h	4.0
4	**15** [18]	TFAA [180]	CH_2_Cl_2_	air	r.t., 24 h	4.2
5	**15** [18]	TFAA [45]	CH_2_Cl_2_	air	40 °C, 24 h	4.5
6	**15** [18]	TFAA [90]	CH_2_Cl_2_	air	40 °C, 24 h	10 *^d^*
7	**15** [18]	TFAA [180]	CH_2_Cl_2_	air	40 °C, 24 h	5.8
8	**15** [50]	TFAA [90]	CH_2_Cl_2_	air	40 °C, 20 h	4.8
9	**15** [18]	TFAA [90]	CHCl_3_	air	40 °C, 24 h	2.4
10	**15** [2]	TFAA [90]	CH_2_Cl_2_	air	40 °C, 24 h	10
11	**15** [2]	TFAA [180]	CH_2_Cl_2_	air	40 °C, 24 h	17 ^e^
12	**15** [18]	Tf_2_O [90]	CH_2_Cl_2_	air	r.t., 16 h	-- ^c^
13	**15** [18]	Tf_2_O [90]	CH_2_Cl_2_	air	40 °C, 16 h	-- ^c^
14	**15** [18]	Tf_2_O [90], PPh_3_O [180]	CH_2_Cl_2_	air	40 °C, 16 h	5.2

^a^ Each reaction was carried out in a 4 mL vial containing a magnetic stir bar. In each reaction, 2.0 mg of reactant (15, 4.5 μmol) was used; ^b^ Yields were determined spectroscopically by the intensity of the Q_y_ band (~741 nm, ε_Qy_ = 120,000 M^−1^cm^−1^ [18,19]) of crude samples. The crude sample was prepared by removal of a specific amount (5 µL from 18 mM, 2.5 µL from 50 mM and 10 µL from 2 mM reactions) of sample from the reaction mixture and dilution with 3 mL of CH_2_Cl_2_; ^c^ No bacteriochlorin formation; ^d^ Reaction at 23 µmol gave 13% yield; ^e^ Reaction at 23 µmol gave 11% yield.

Treatment of the dihydrodipyrrin–carbinol **15** (18 mM) with trifluoroacetic anhydride (TFAA) (45 mM) at room temperature for 24 h gave the meso-tetraarylbacteriochlorin in 1.8% yield (entry 2), and with 90 or 180 mM TFAA, the yield was ~4% (entries 3 and 4). The reaction at 40 °C gave better yields compared to room temperature, with TFAA at 45 mM (4.5%), 90 mM (10%), or 180 mM (5.8%) (entries 5–7). Maintaining the TFAA concentration fixed (90 mM) but varying the concentration of **15** (entries 8–10) gave 10% yield at 2 mM (entry 10); a further increase in TFAA concentration to 180 mM gave 17% yield (entry 11). Although a 17% yield was observed by using 180 mM of TFAA and 2 mM of **15** (4.5 μmol), increasing the scale of the reaction to 23 μmol gave only 11% yield (entry 11 footnote). The reaction of **15** with triflic anhydride (entries 12 and 13) or in combination with triphenylphosphine oxide (entry 14) failed to improve the yield.
