# Supplementary material for: Synthesis and Spectral Properties of meso-Arylbacteriochlorins, Including Insights into Essential Motifs of their Hydrodipyrrin Precursors
Source: Molecules. 2017 Apr 14;22(4):634. doi: 10.3390/molecules22040634 (PMC6154299; doi:10.3390/molecules22040634)
Supplement: Supplementary file 1 [file molecules-22-00634-s001.pdf]

Supporting Information for:

Synthesis and Spectral Properties of Meso-Arylbacteriochlorins Including Insights into Essential Motifs of Hydrodipyrrin Precursors

Muthyala Nagarjuna Reddy, Shaofei Zhang, Han-Je Kim, Olga Mass,
Masahiko Taniguchi and Jonathan S. Lindsey

Table of Contents

Section	Topic	Page
I.	Spectral data	S2

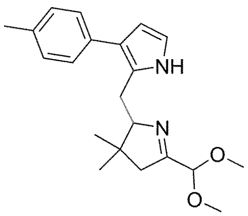

4

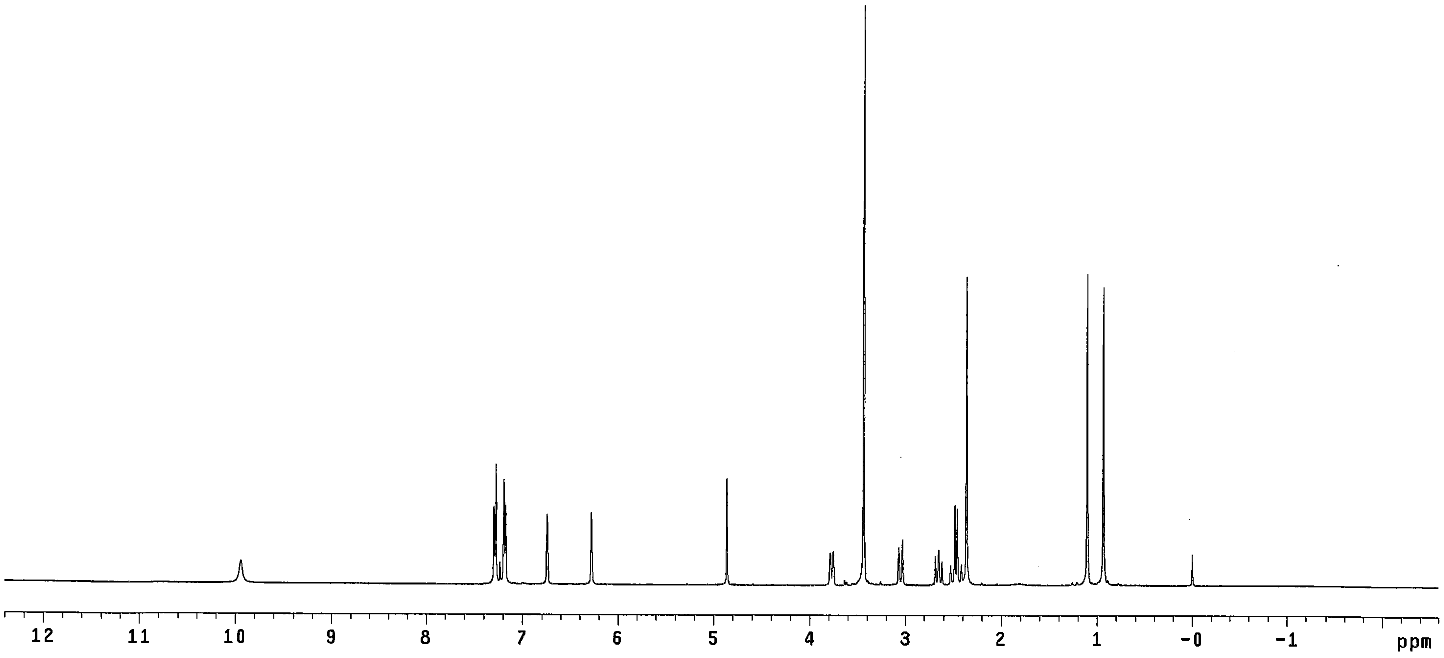

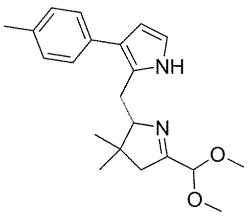

4

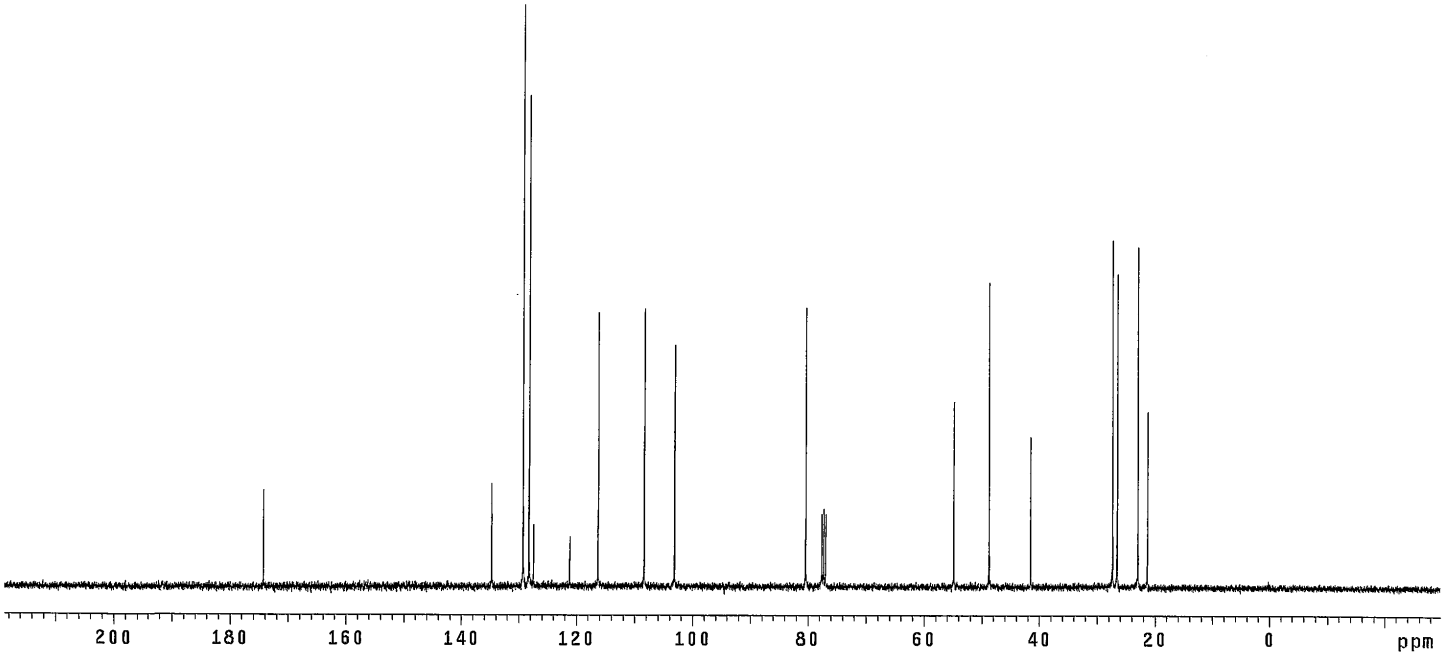

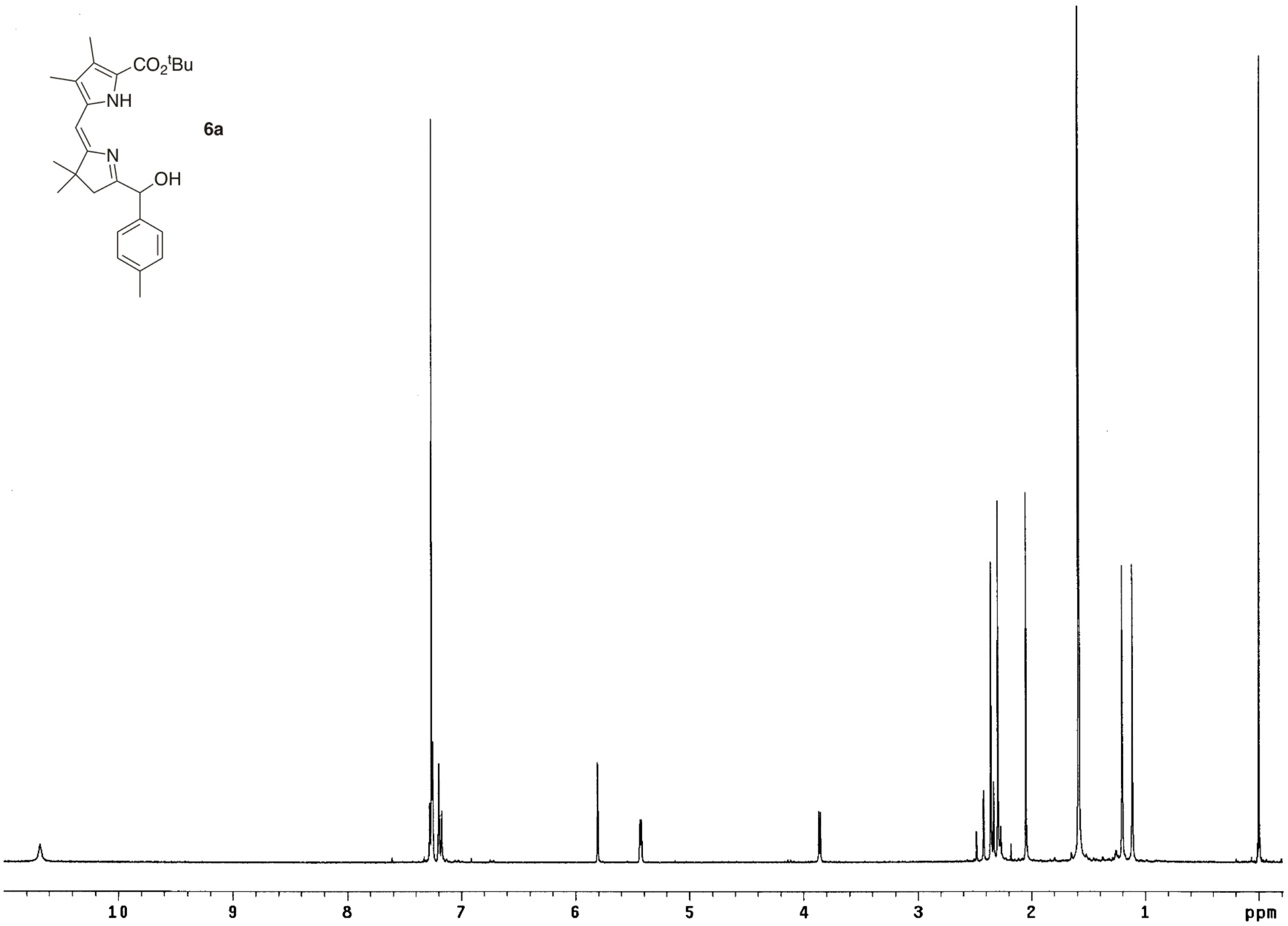

CO2tBu
NH
N
OH
6a
10
9
8
7
6
5
4
3
2
1
ppm

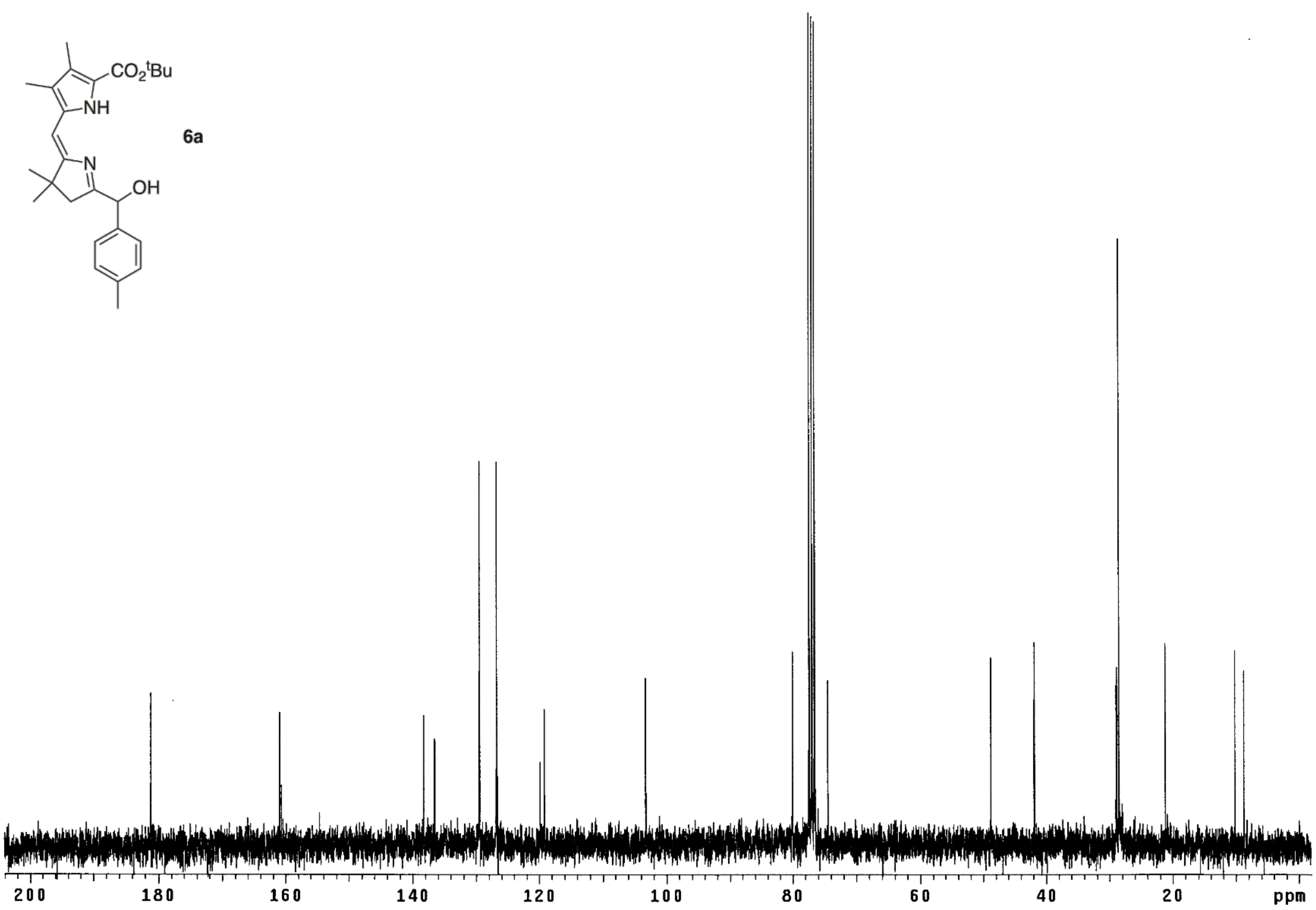
CO2tBu
NH
N
OH
6a
200
180
160
140
120
100
80
60
40
20
ppm

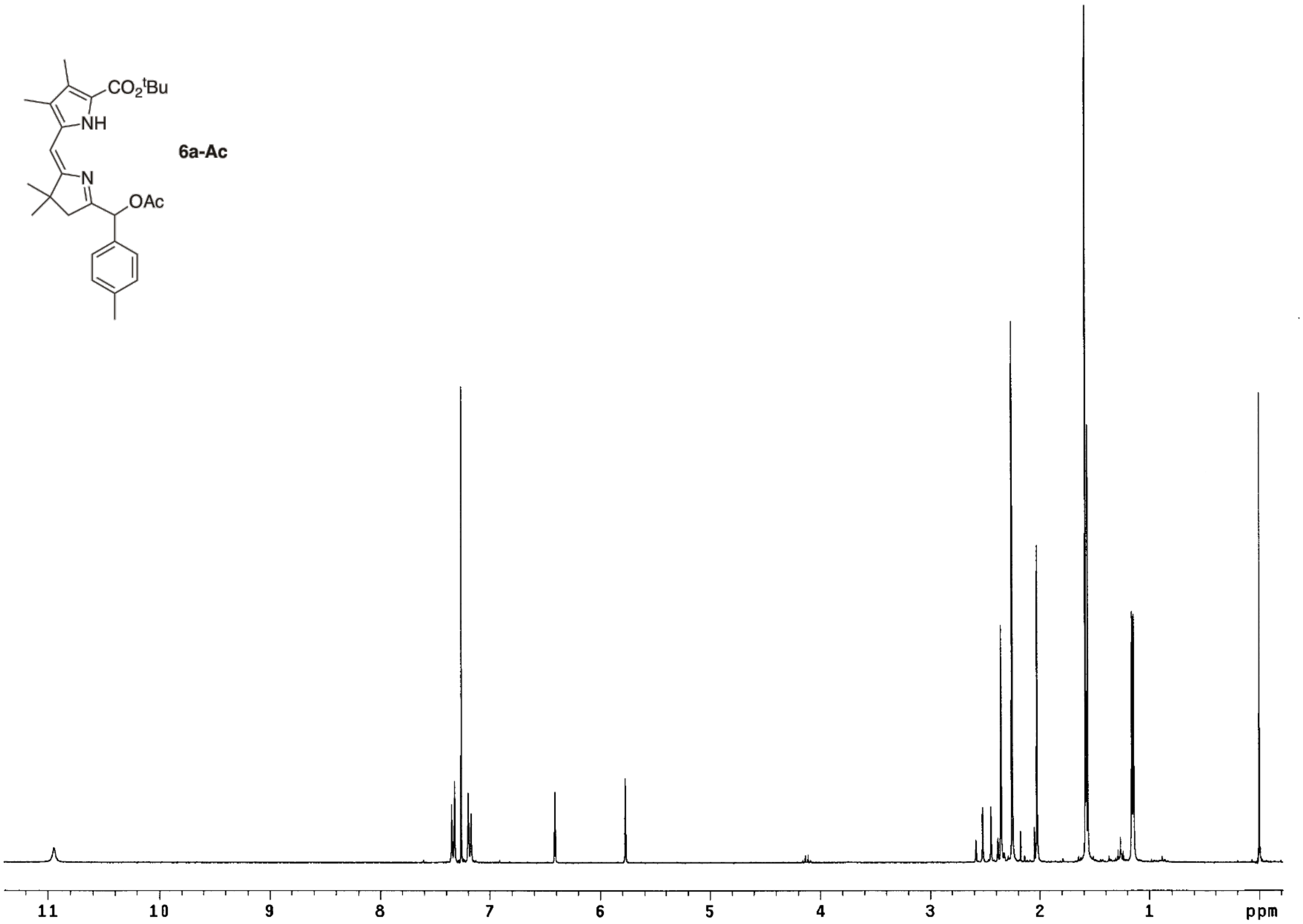

CO2tBu
NH
6a-Ac
N
OAc
11
10
9
8
7
6
5
4
3
2
1
ppm

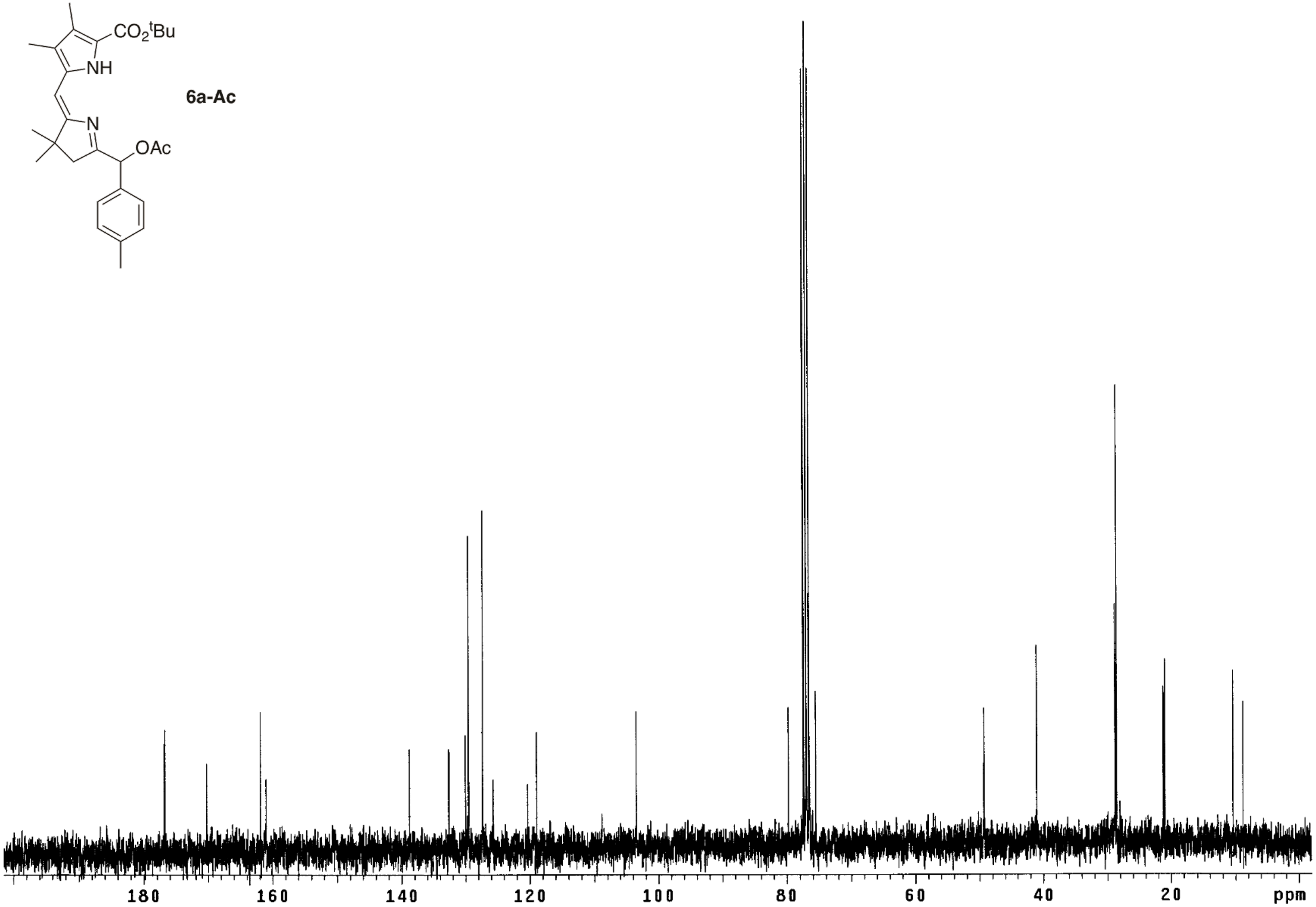

CO2tBu
NH
N
OAc
6a-Ac
180
160
140
120
100
80
60
40
20
ppm

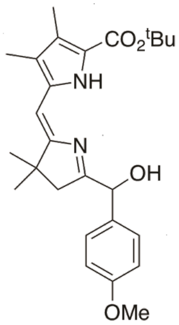

6b

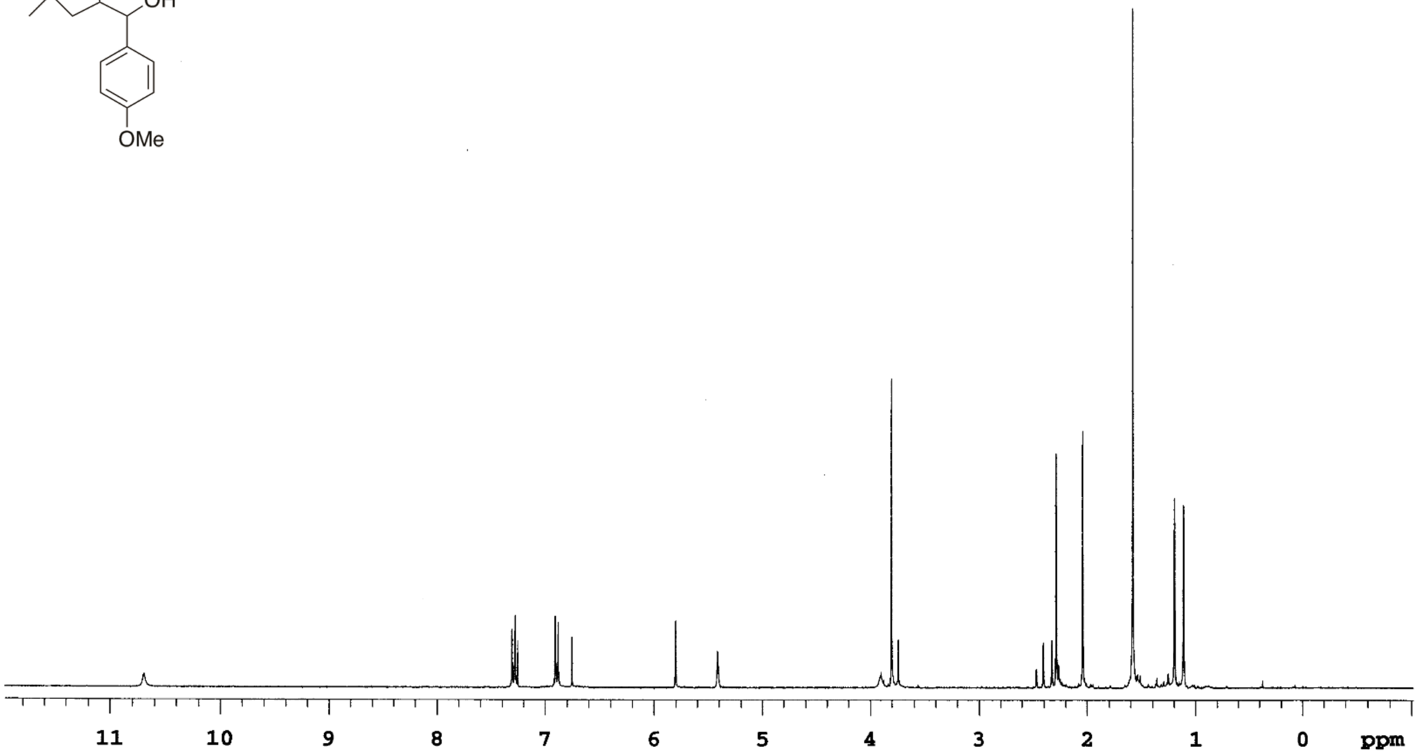

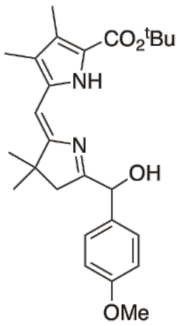

6b

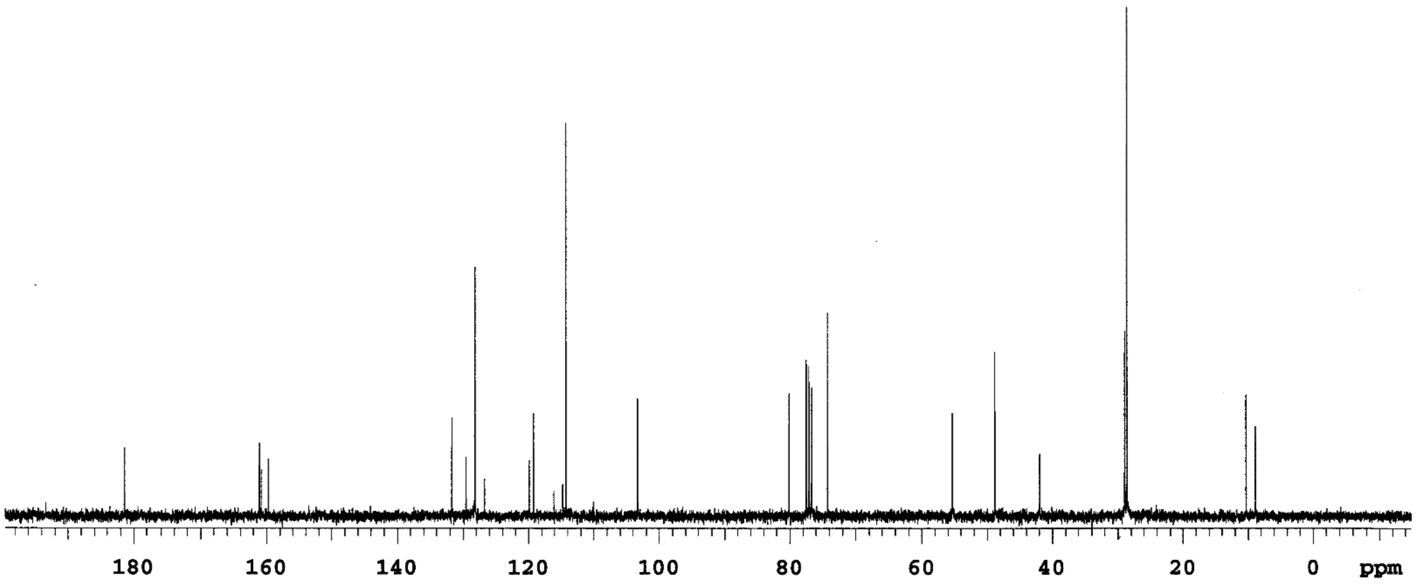

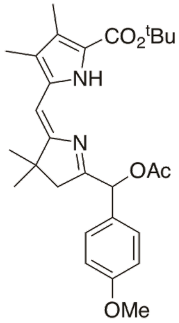

6b-Ac

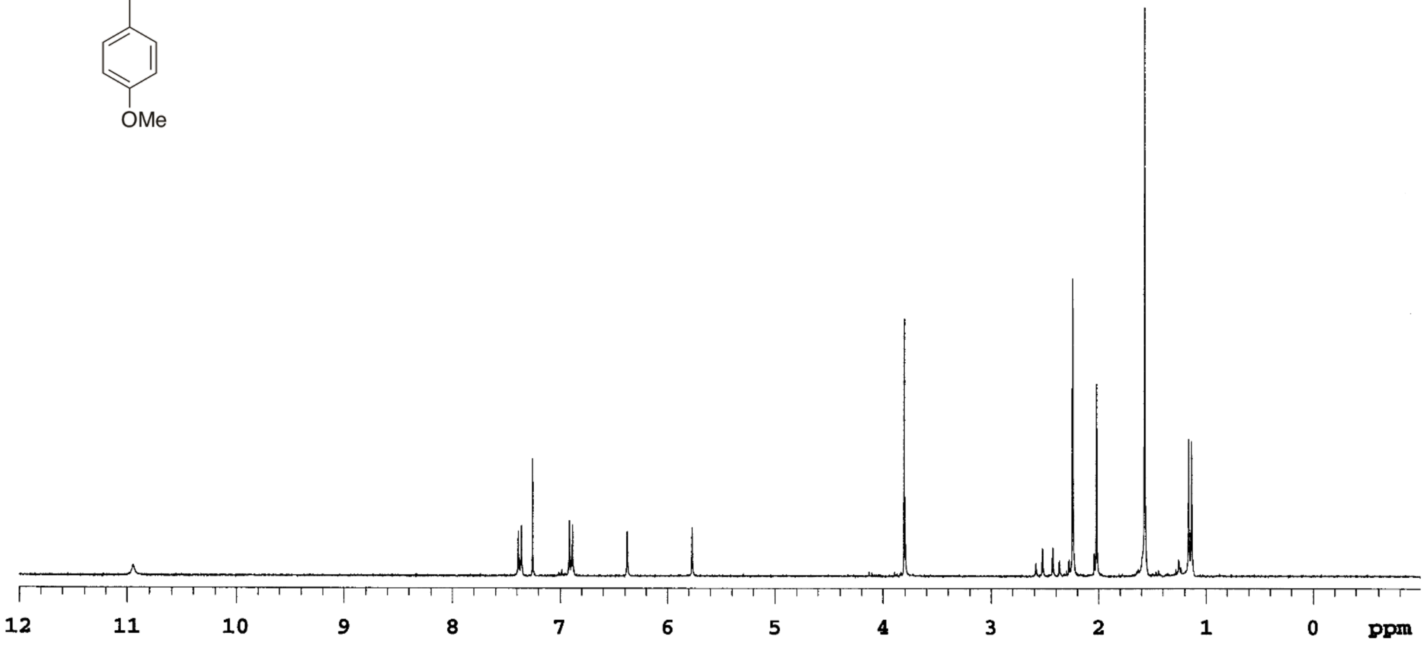

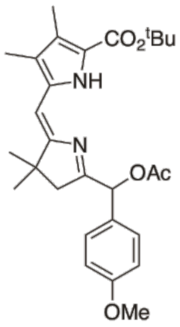

6b-Ac

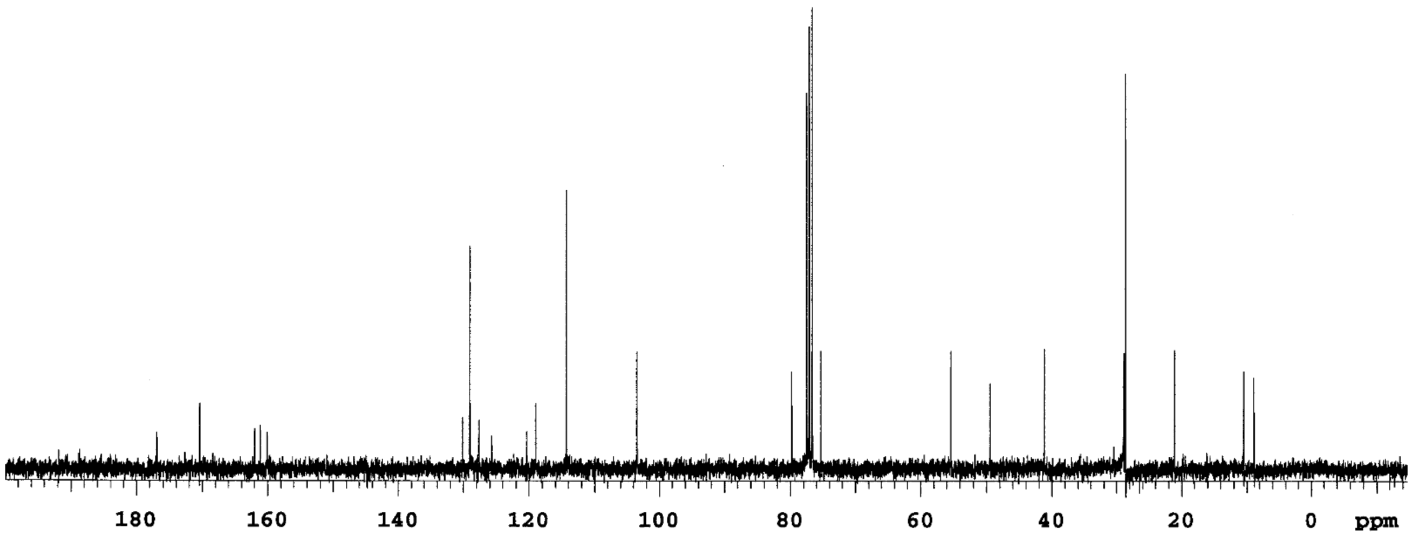

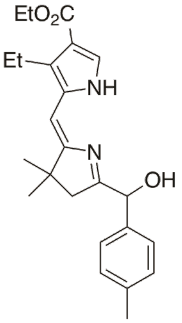

8a

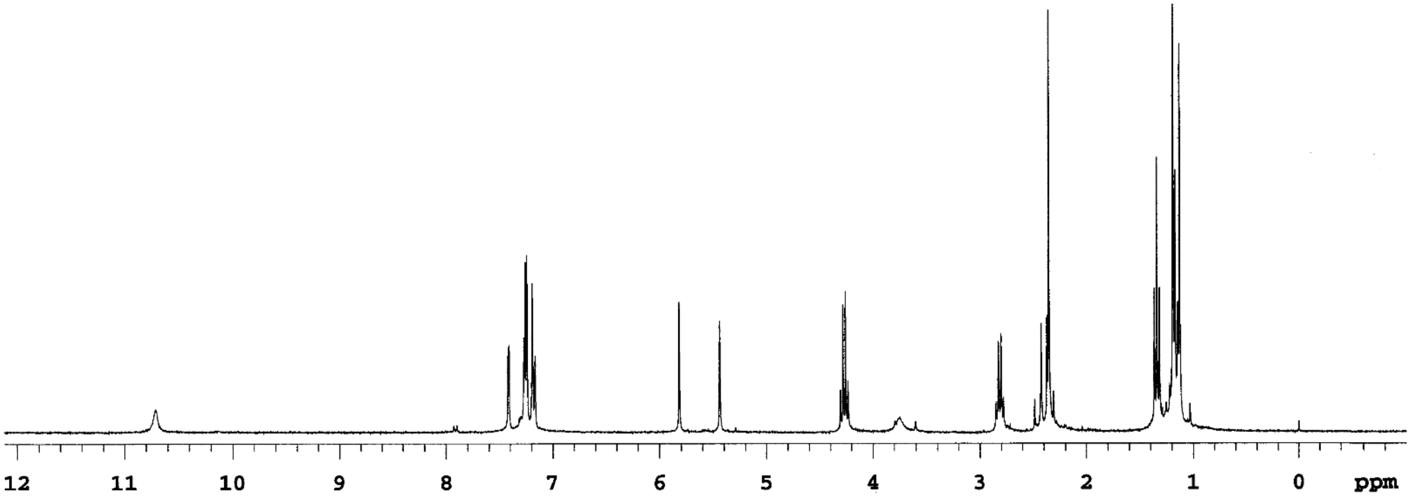

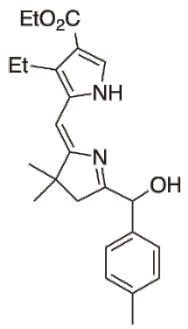

8a

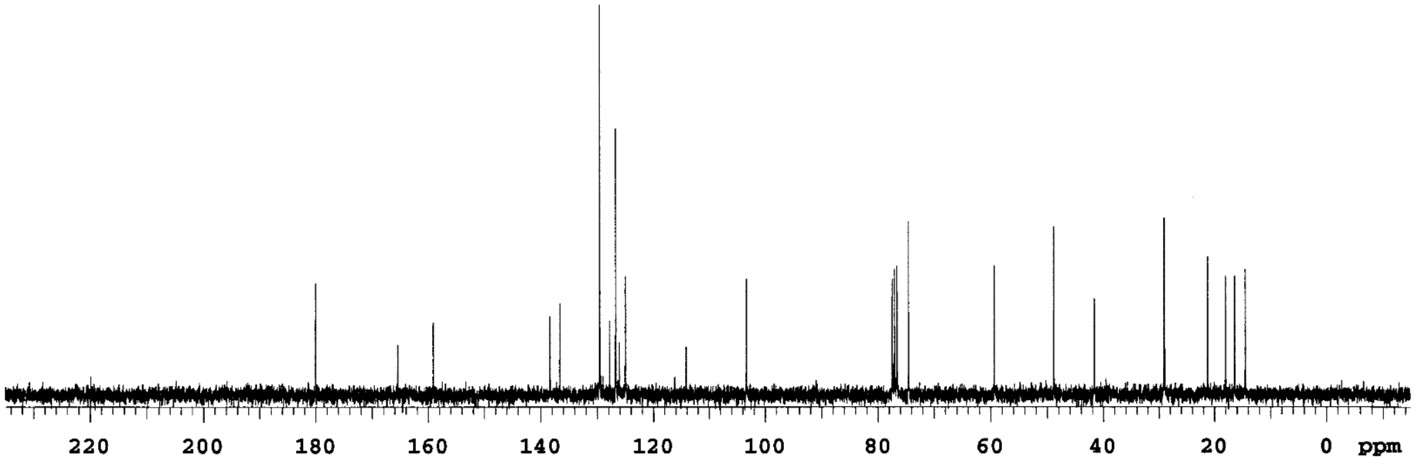

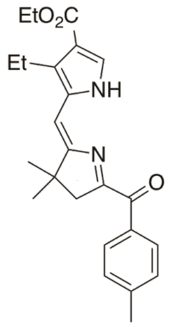

8a-Ox

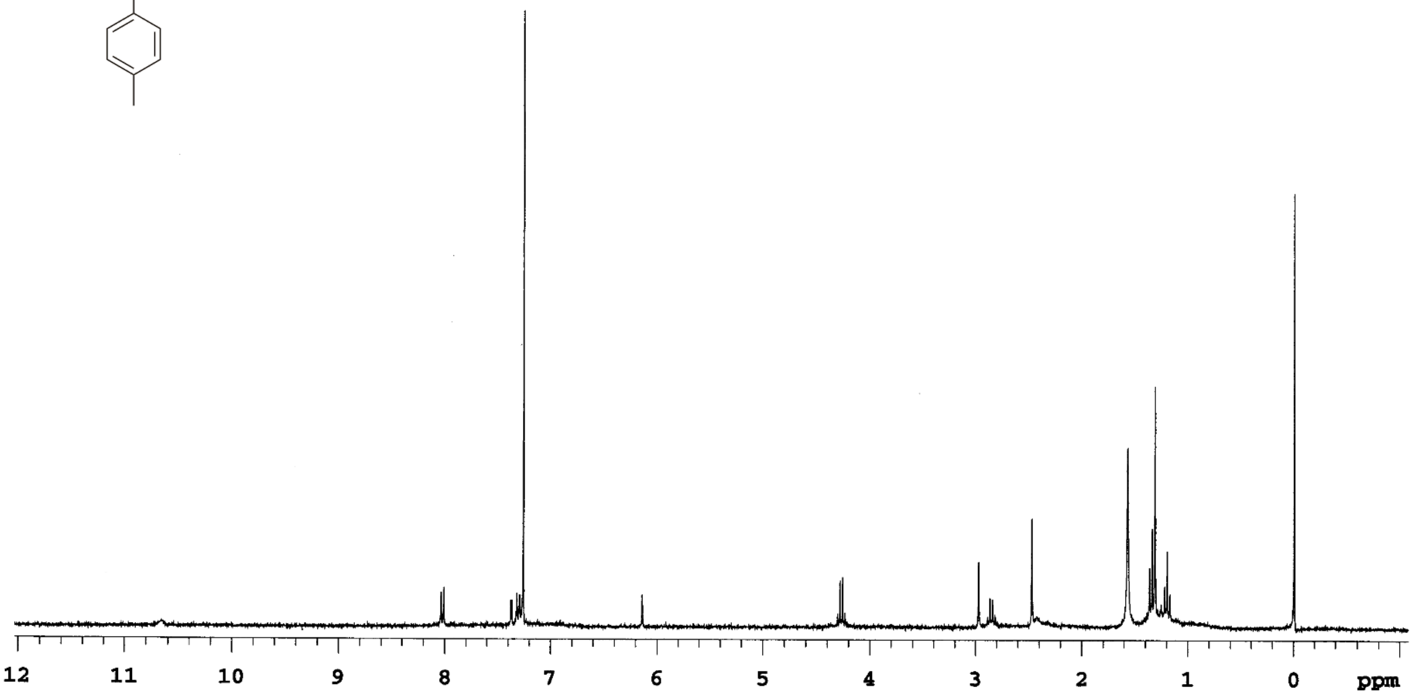

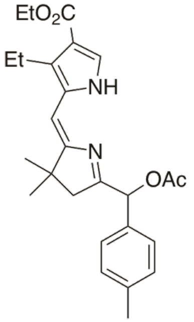

8a-Ac

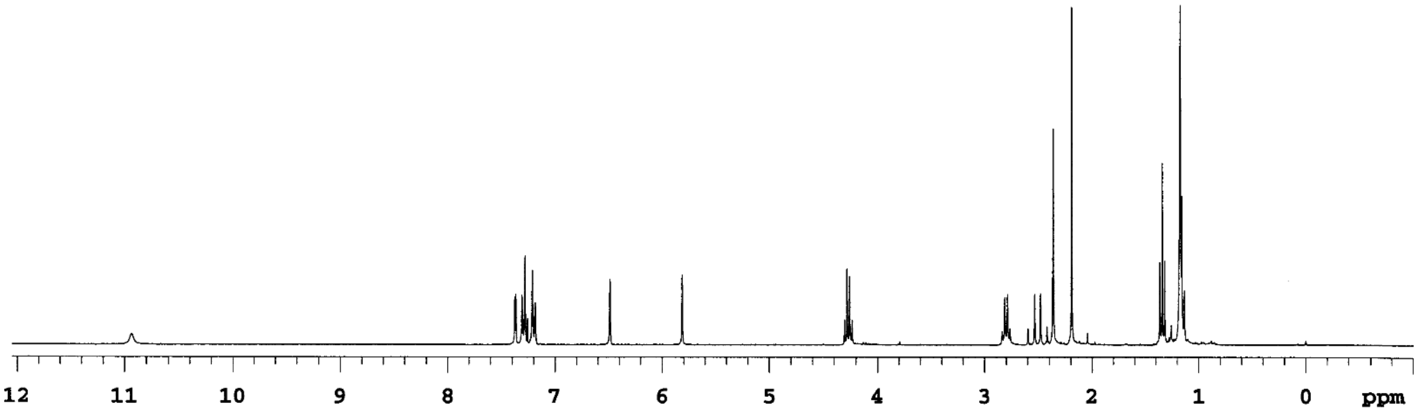

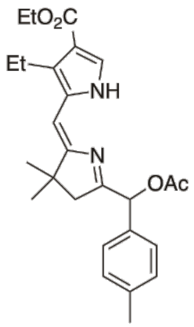

8a-Ac

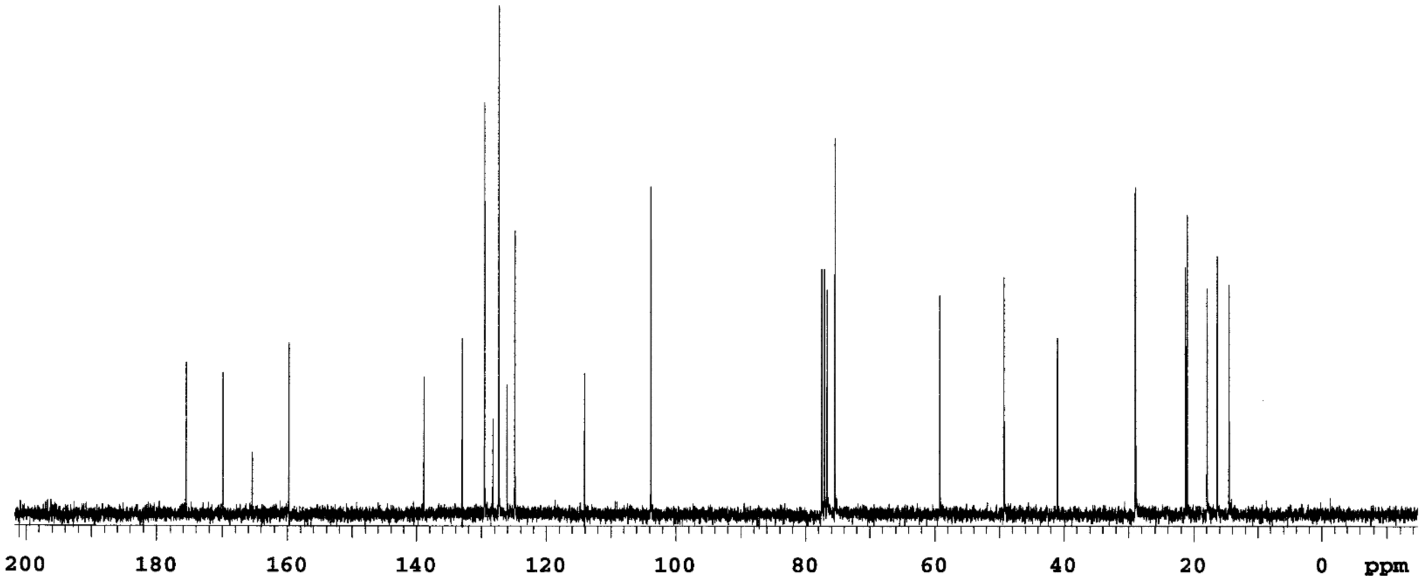

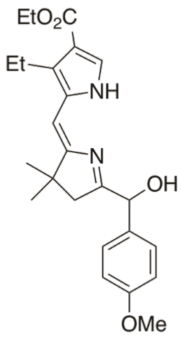

8b

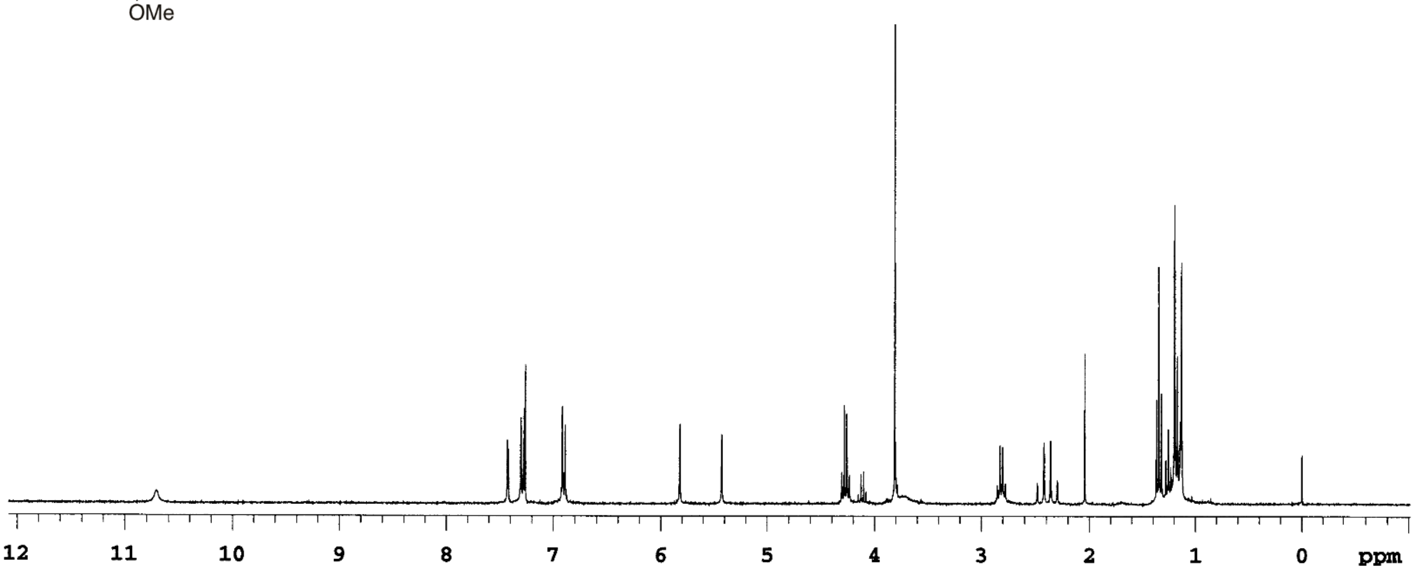

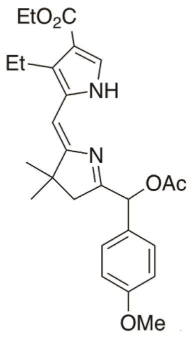

8b-Ac

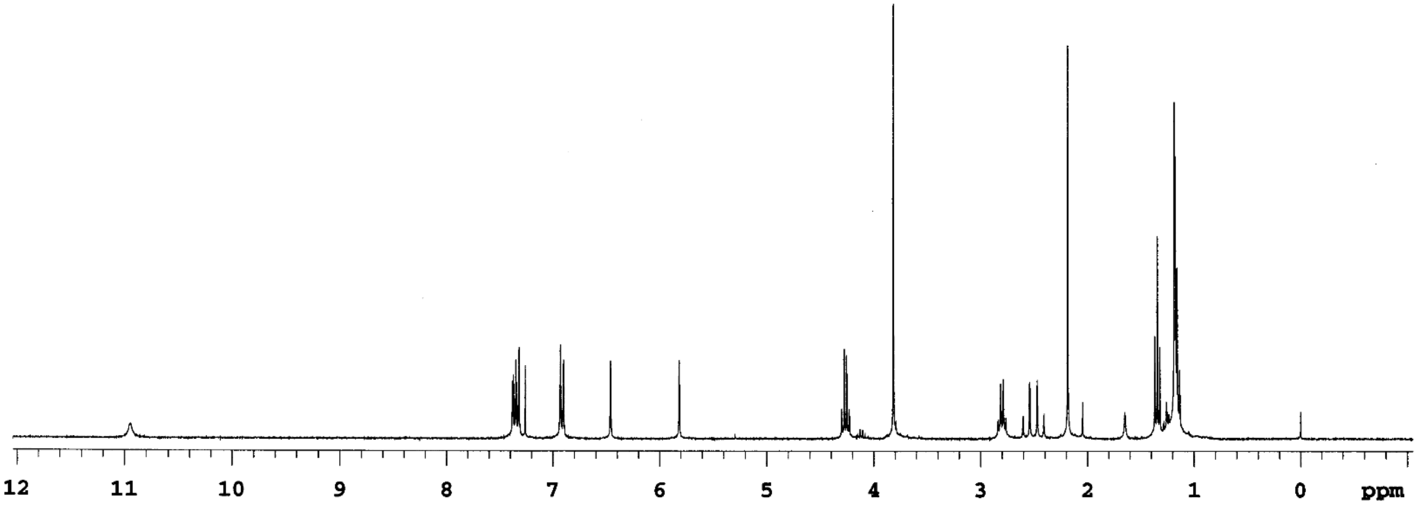

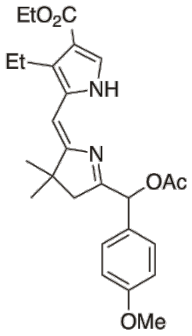

8b-Ac

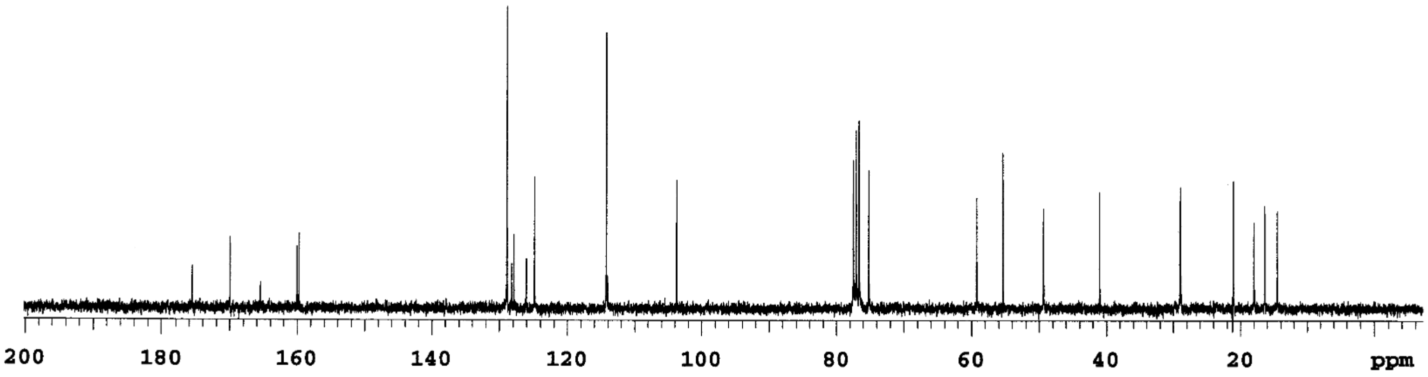

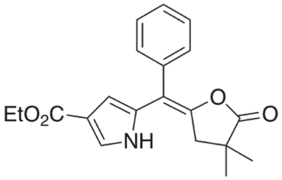

11

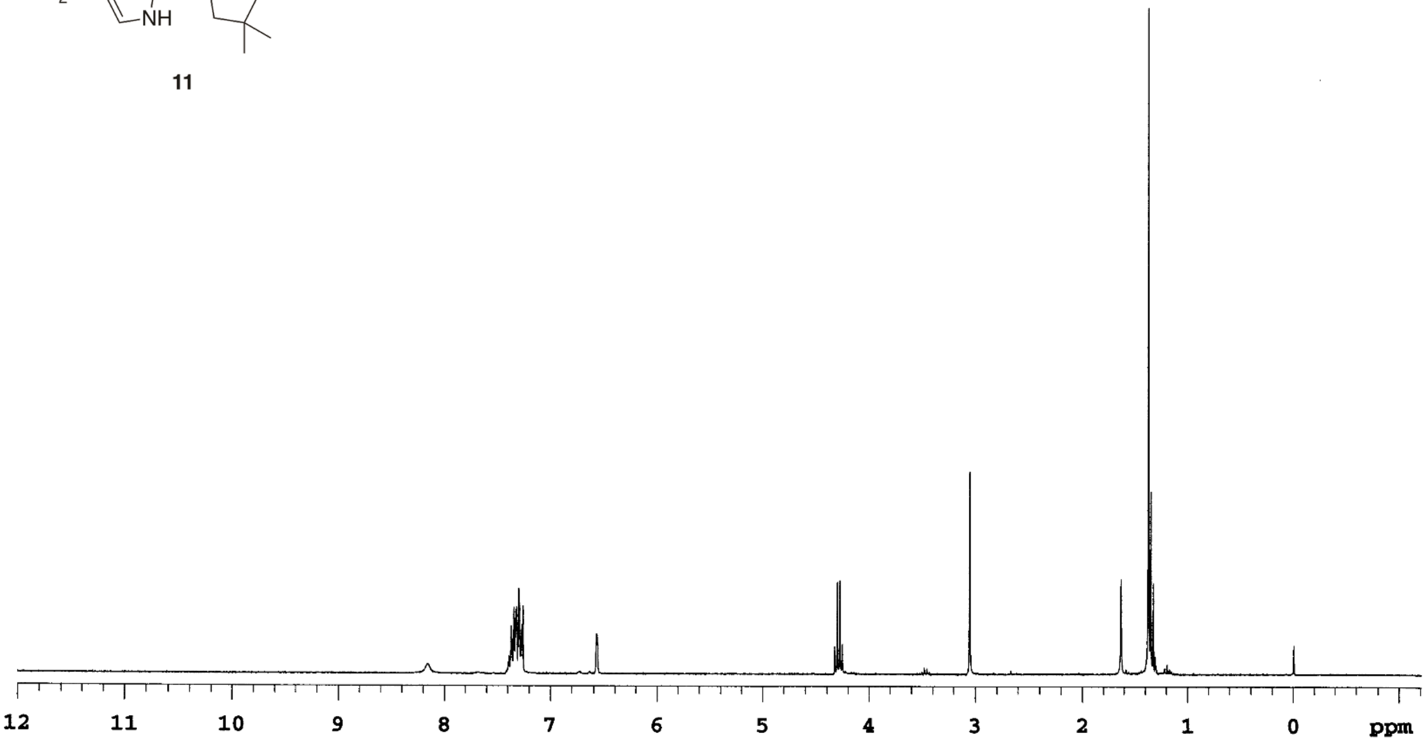

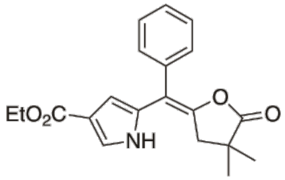

11

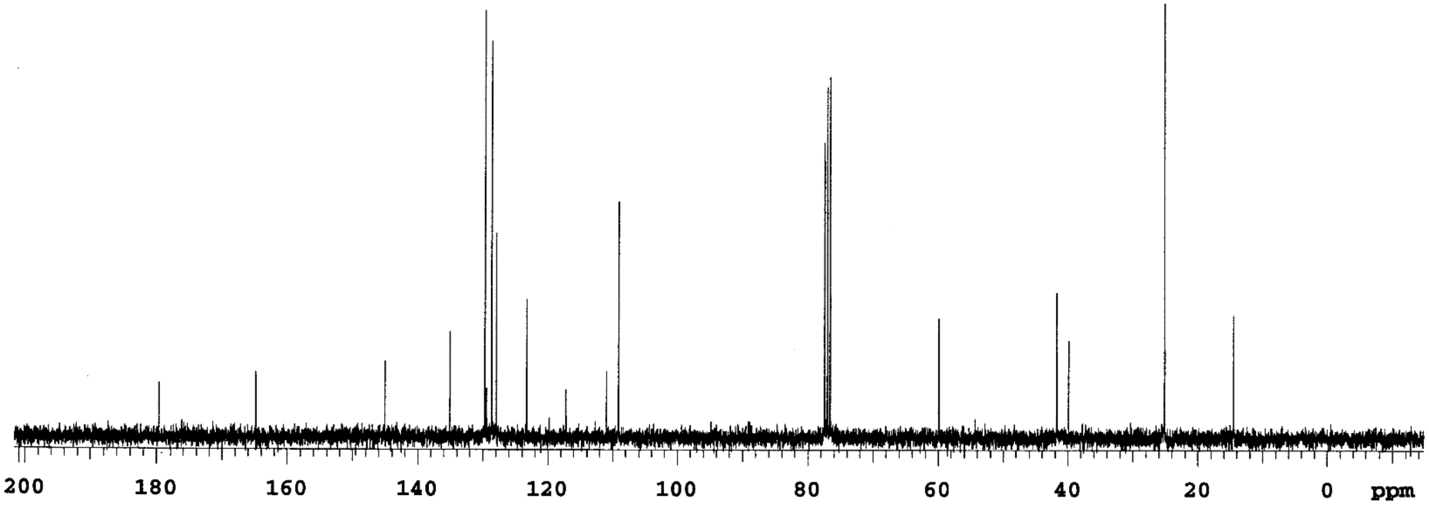

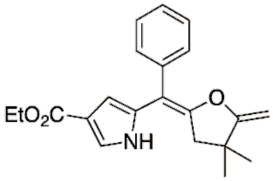

12

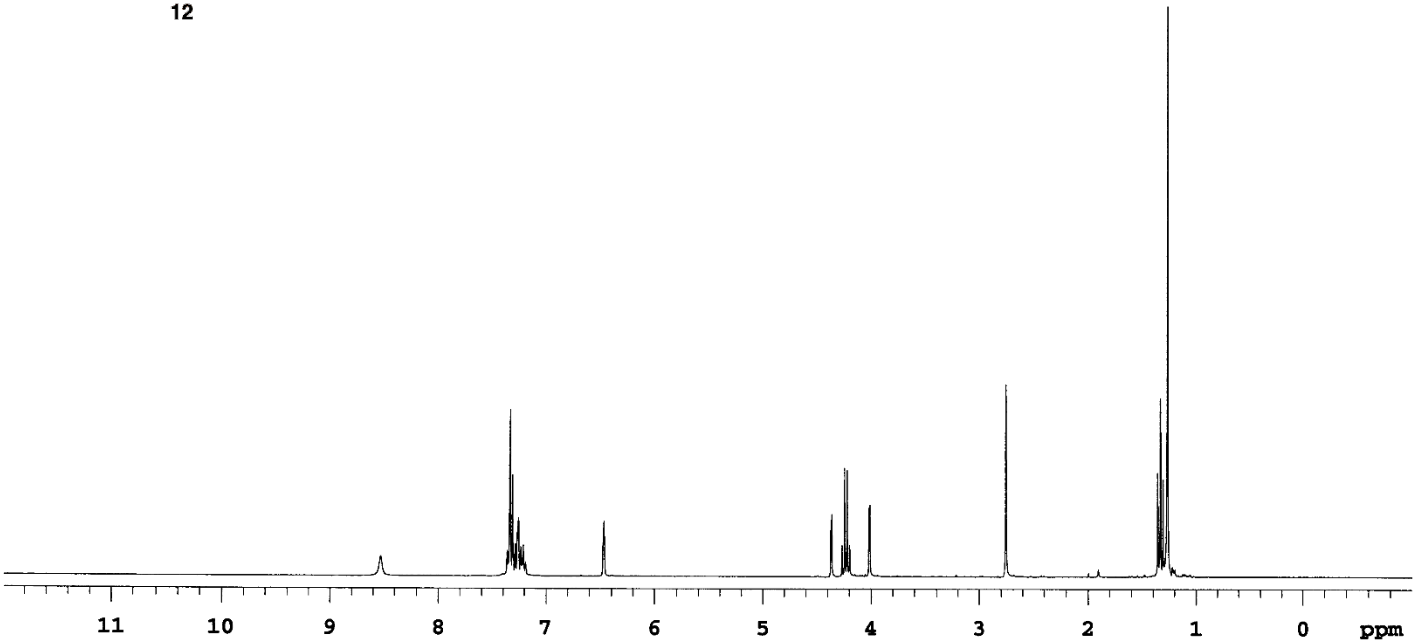

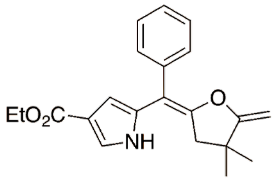

12

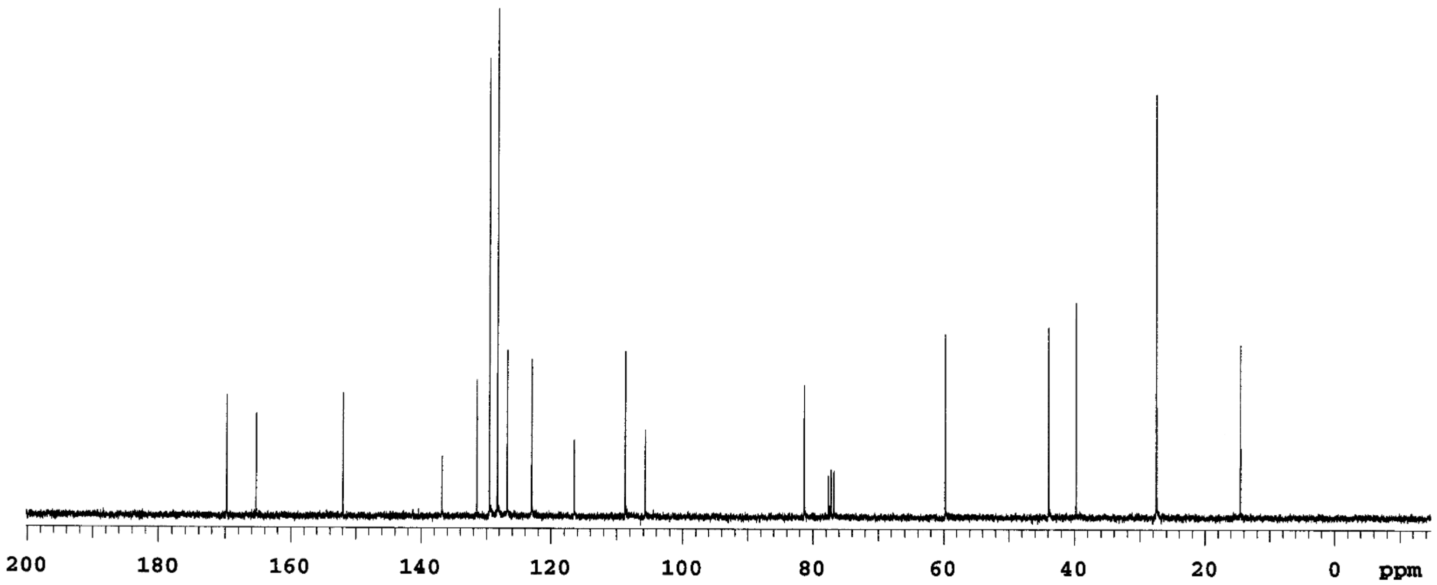

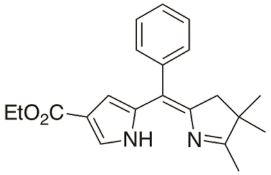

13

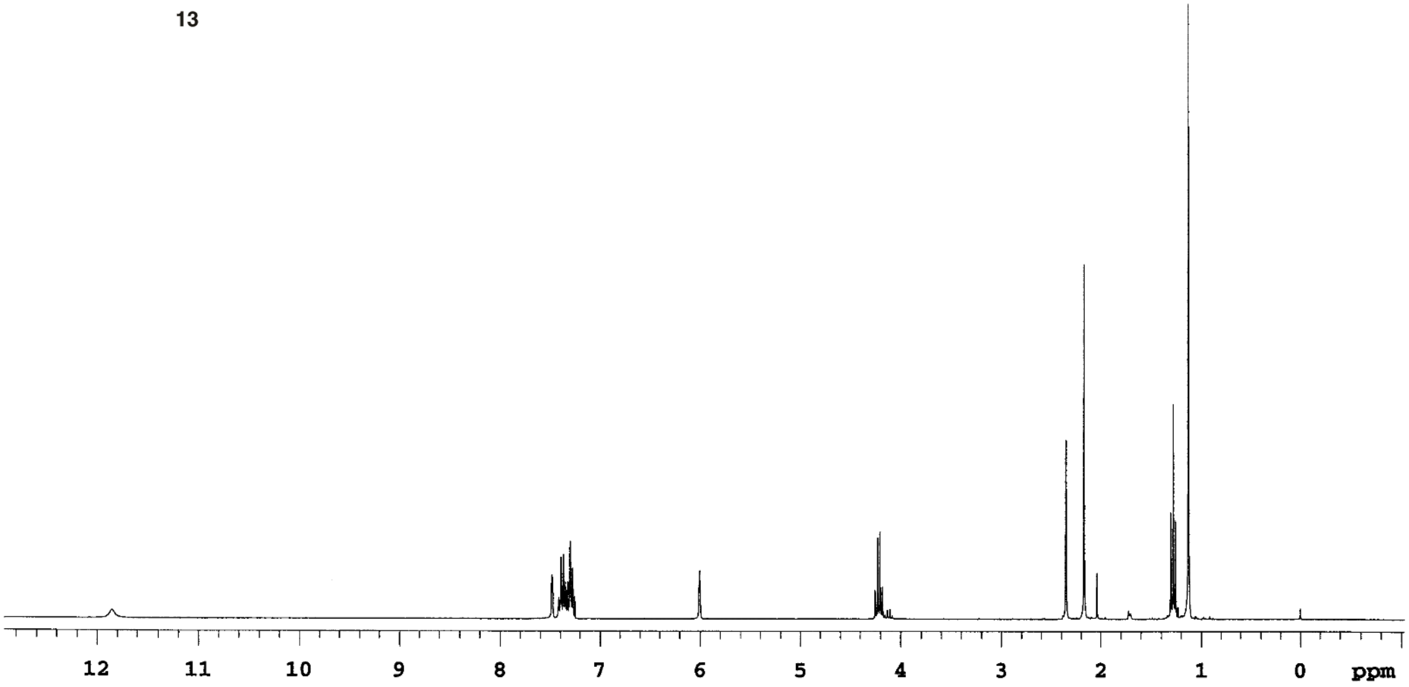

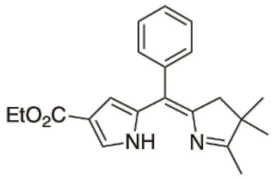

13

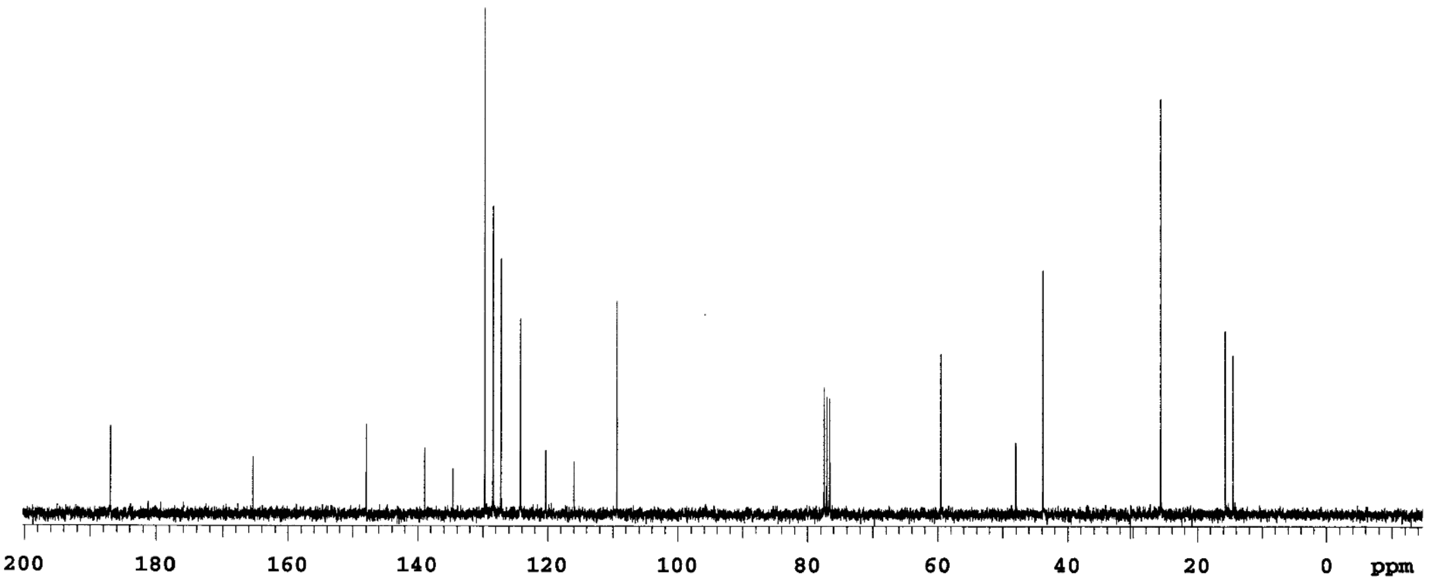

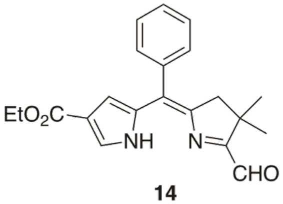

14

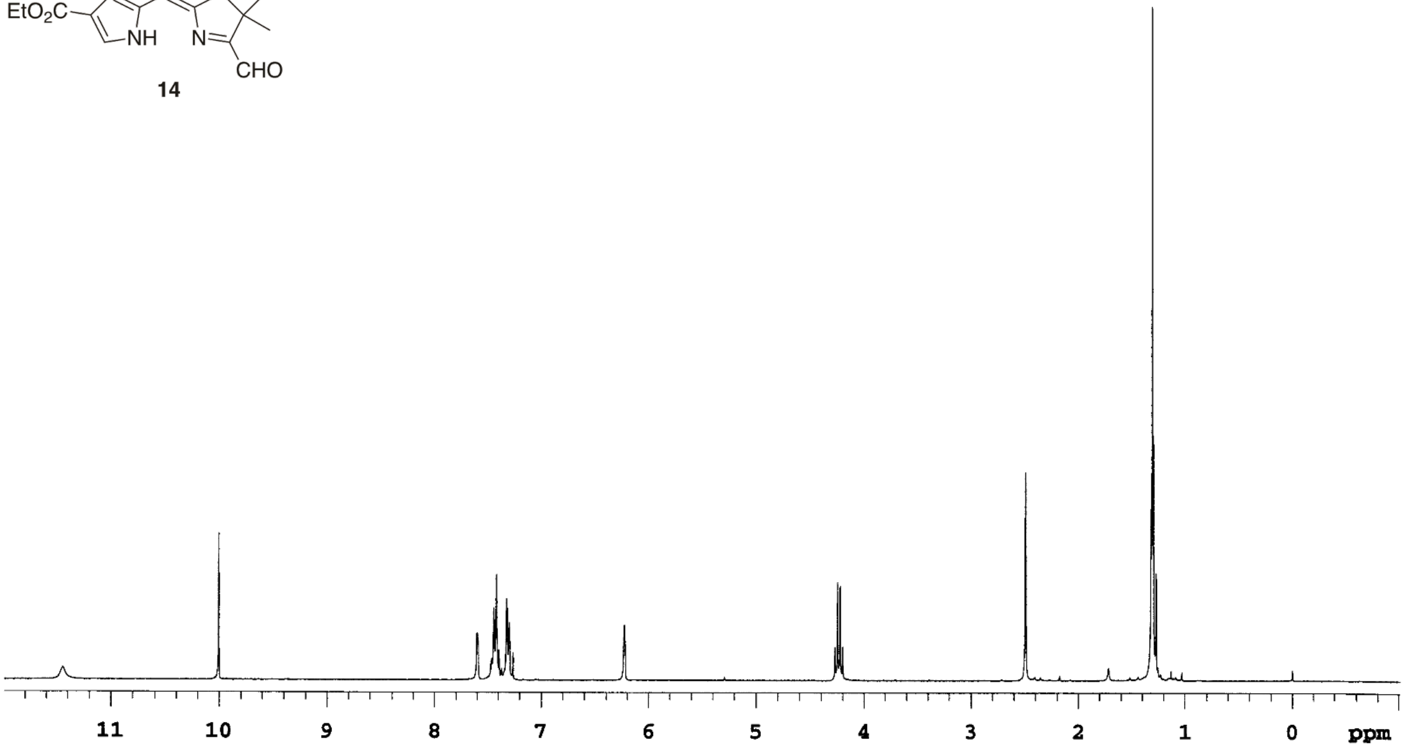

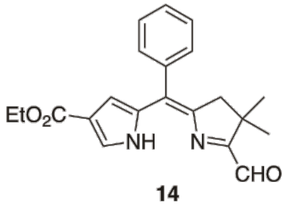

14

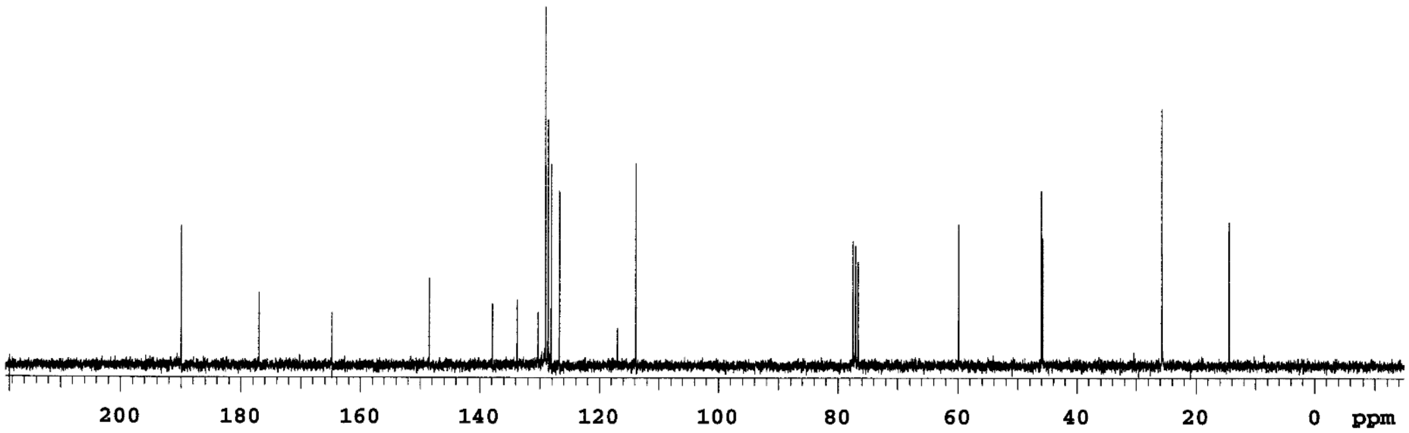

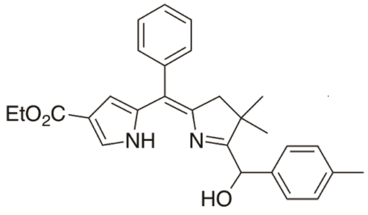

15

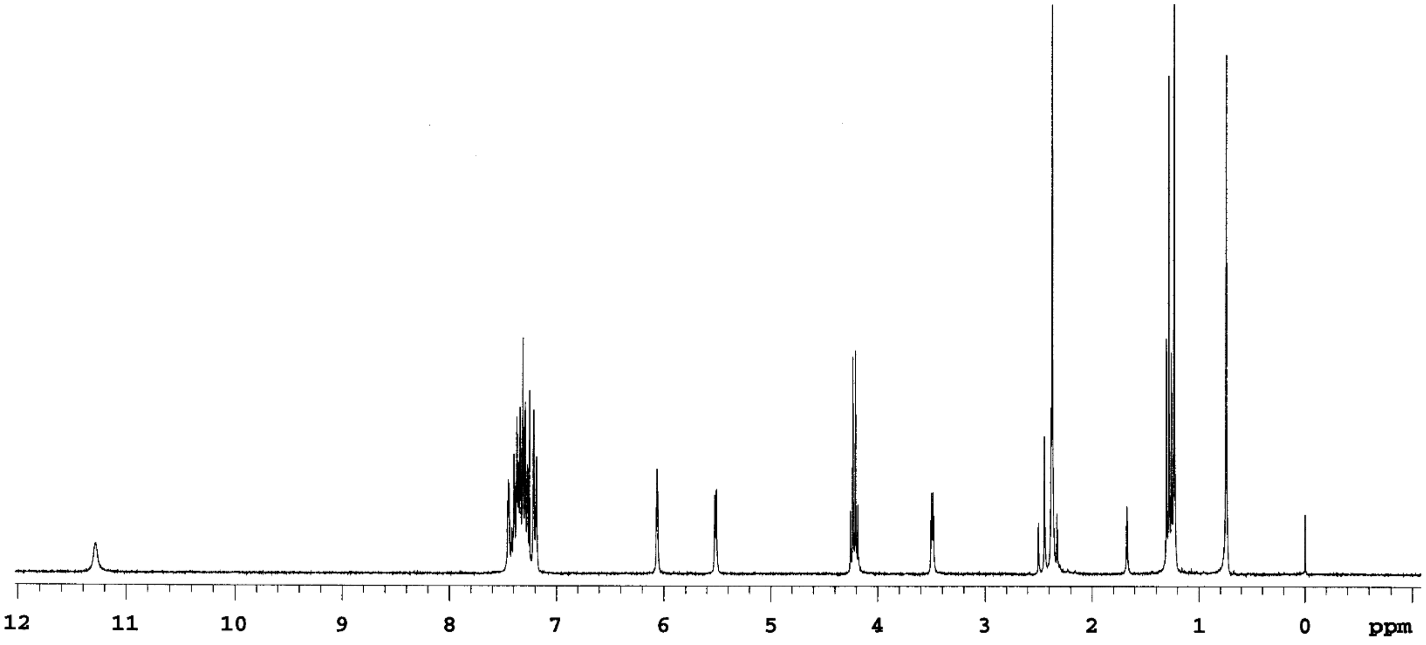

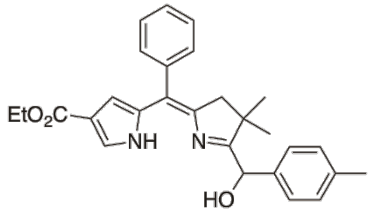

15

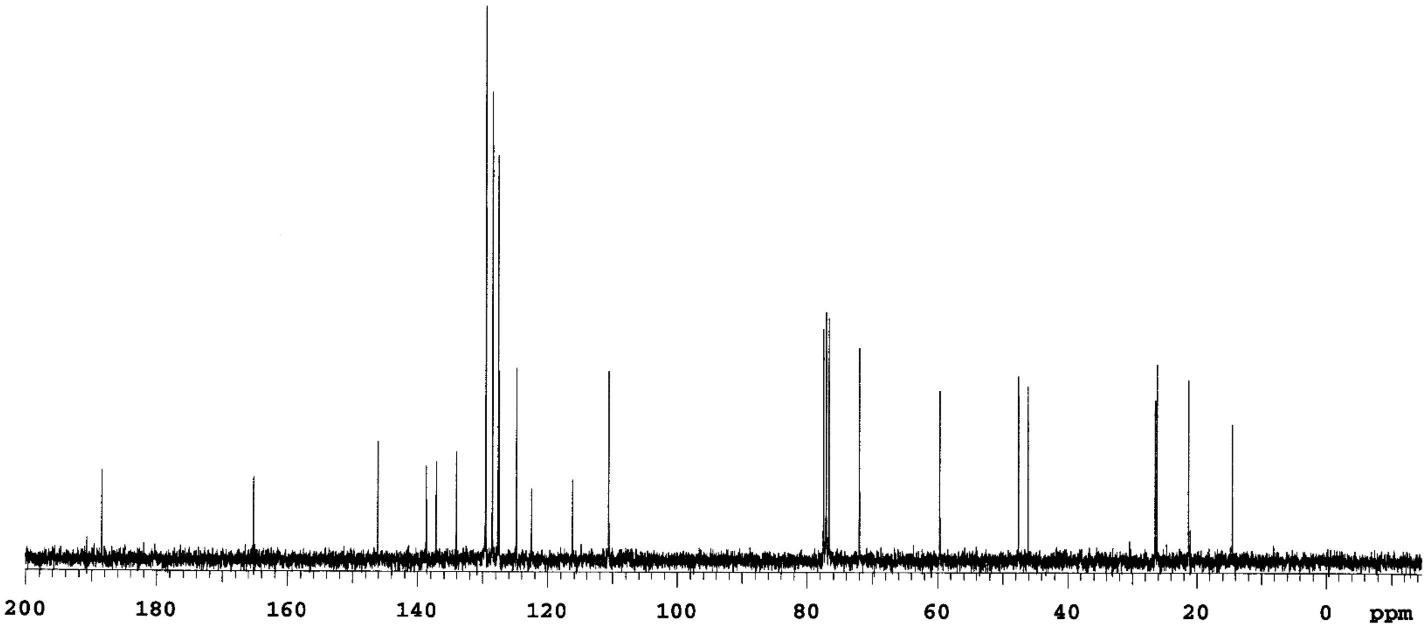

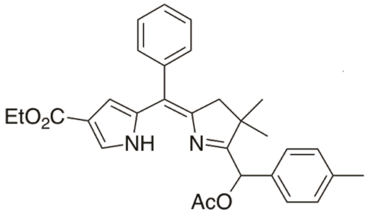

15-Ac

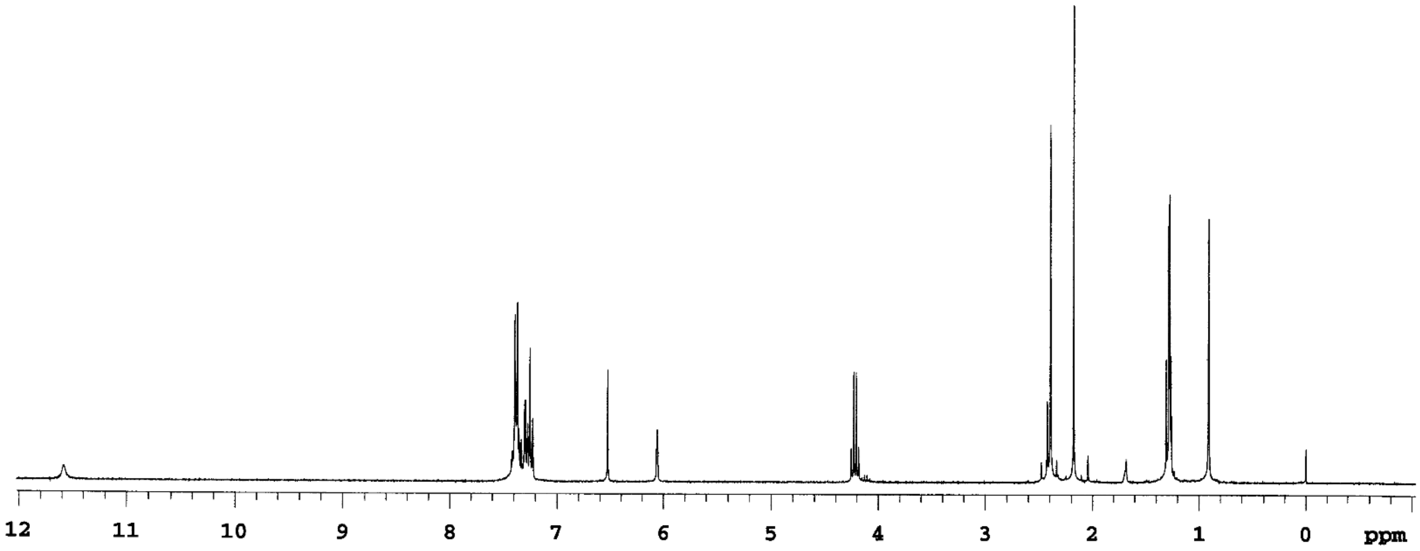

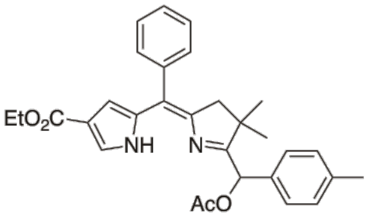

15-Ac

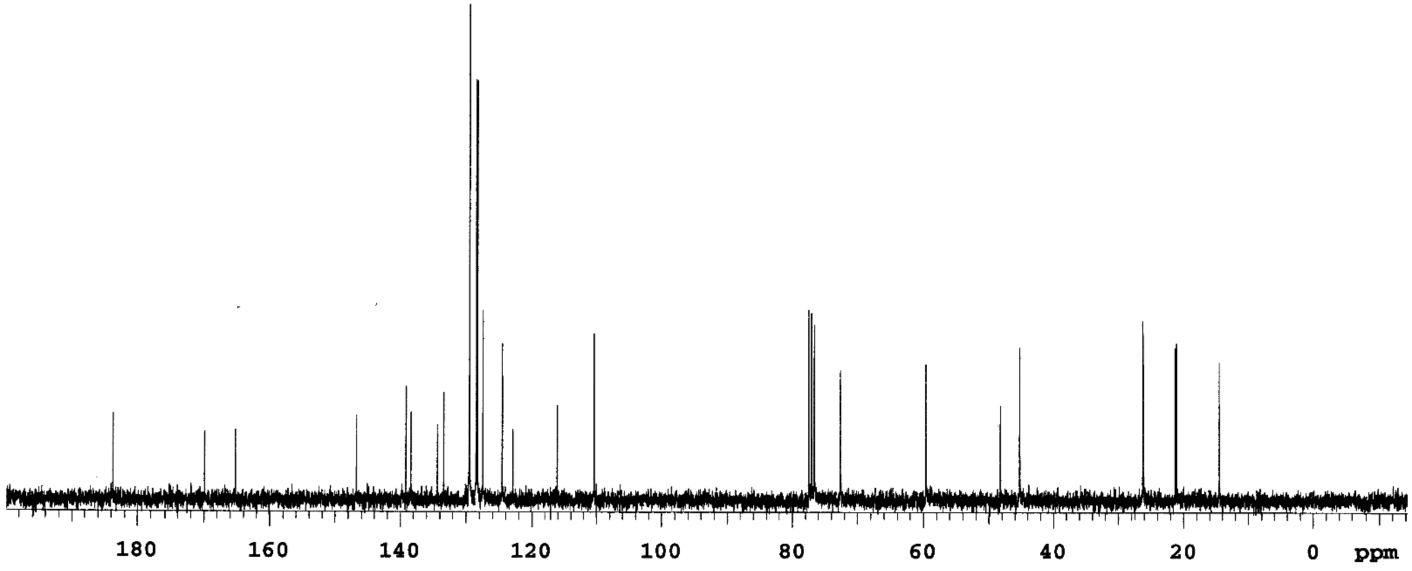

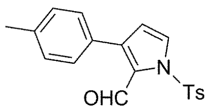

17

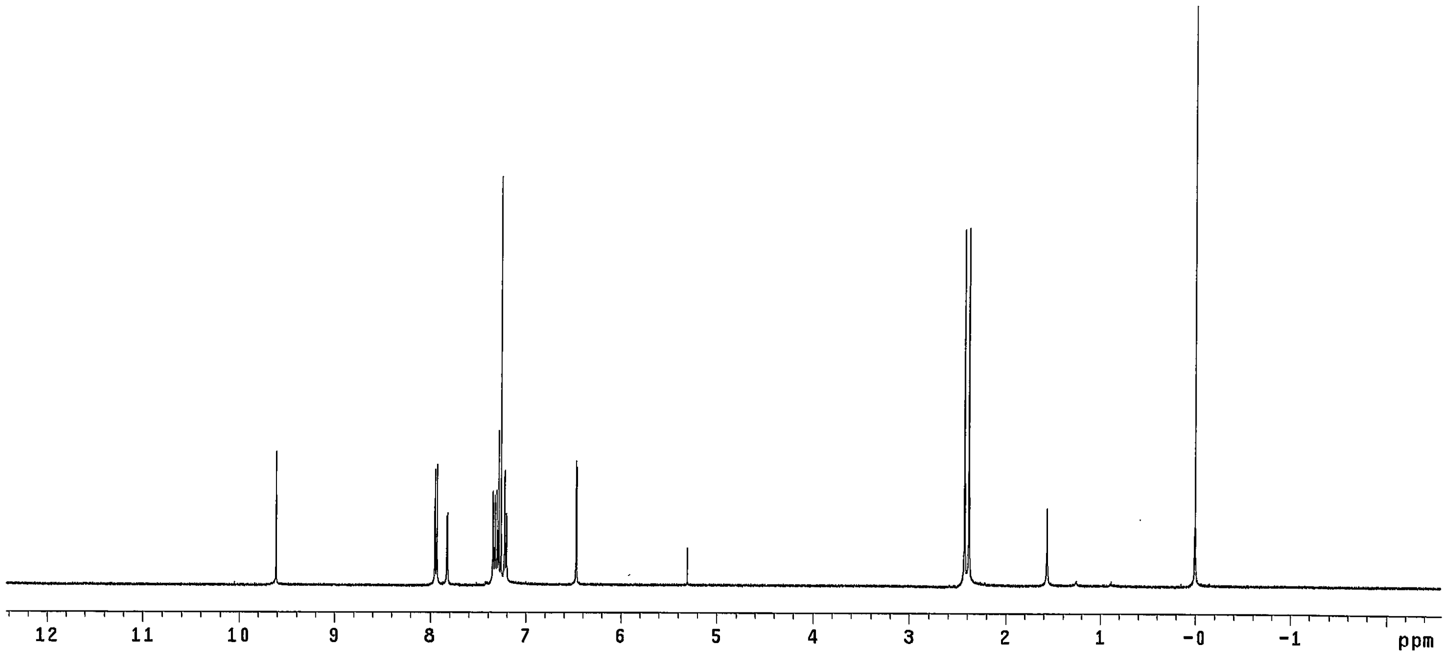

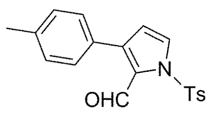

17

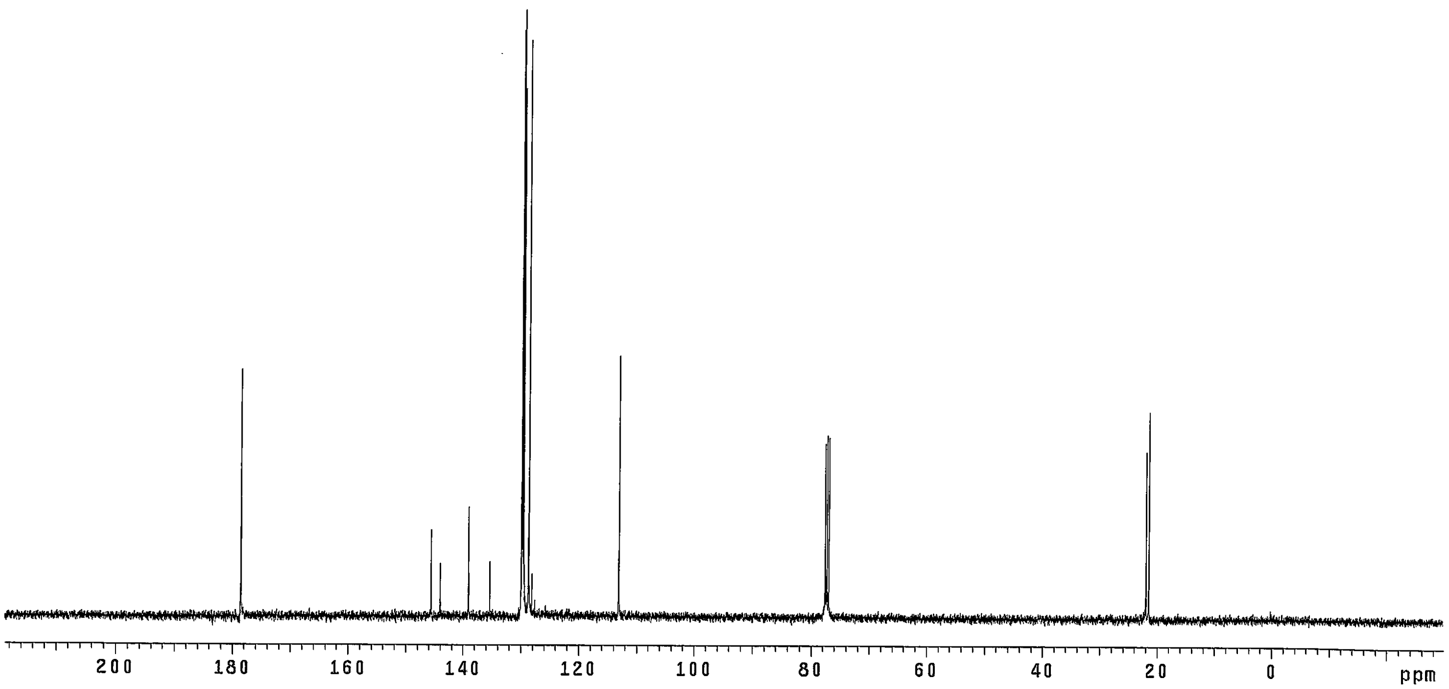

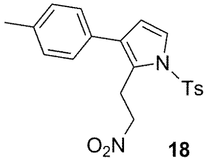

18

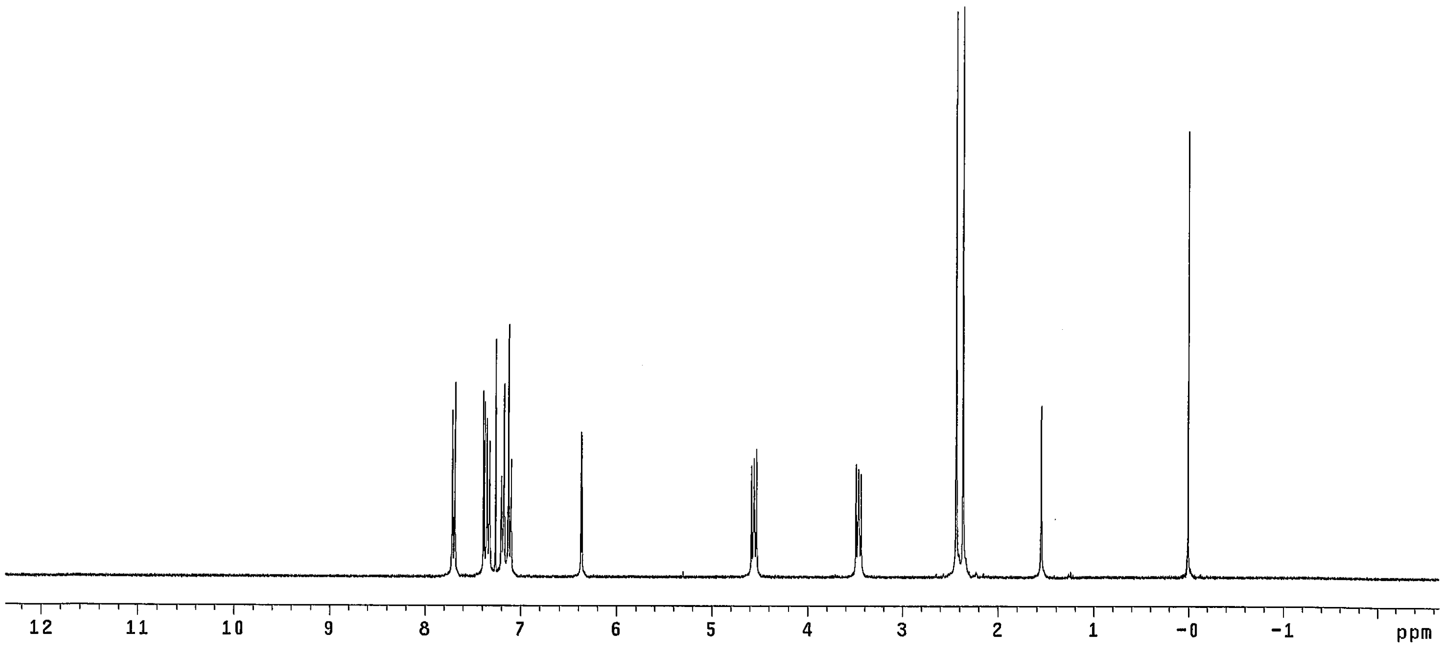

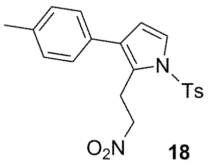

18

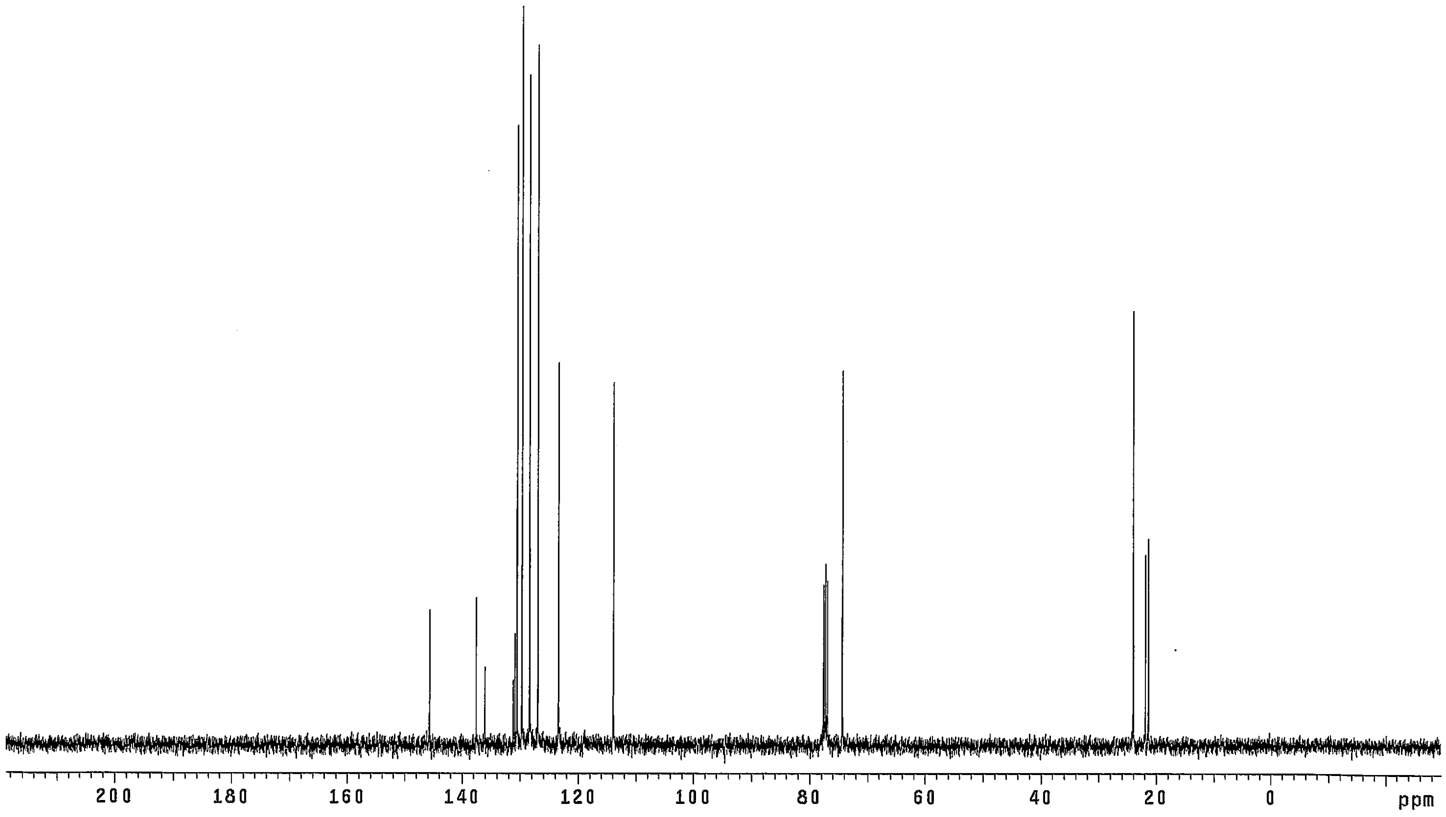

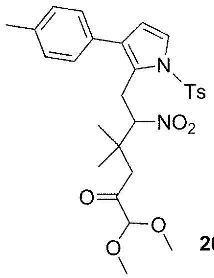

20

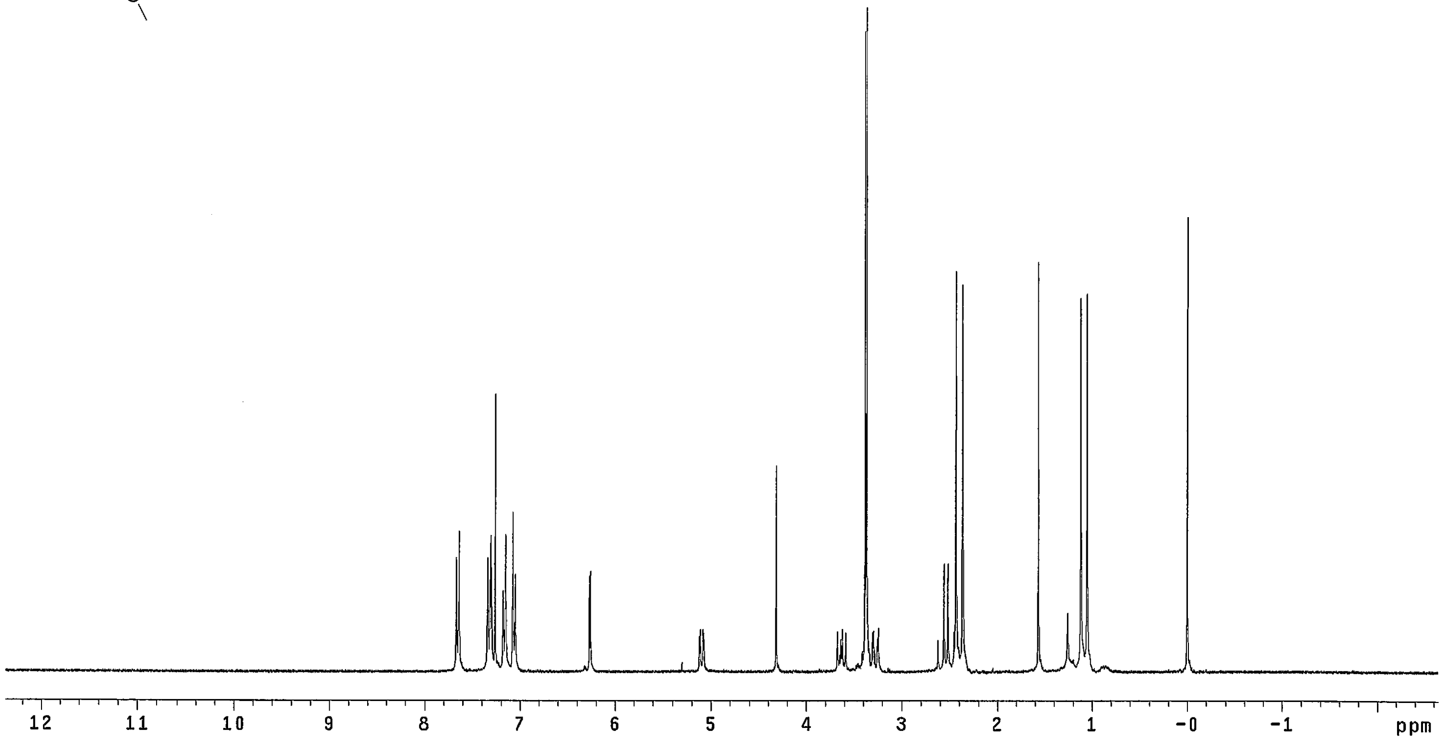

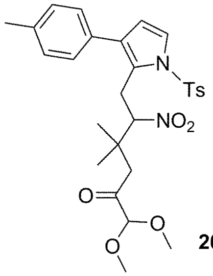

20

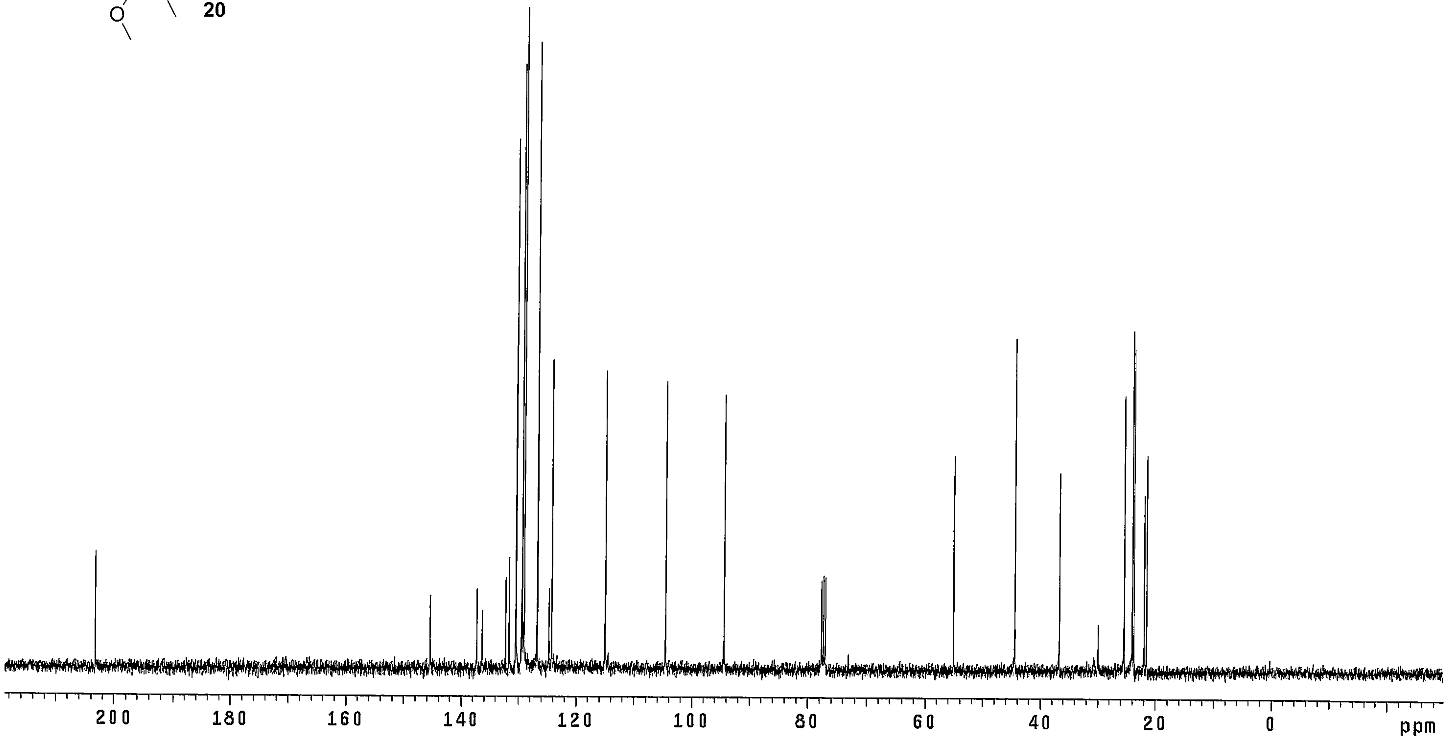

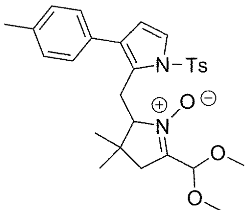

21

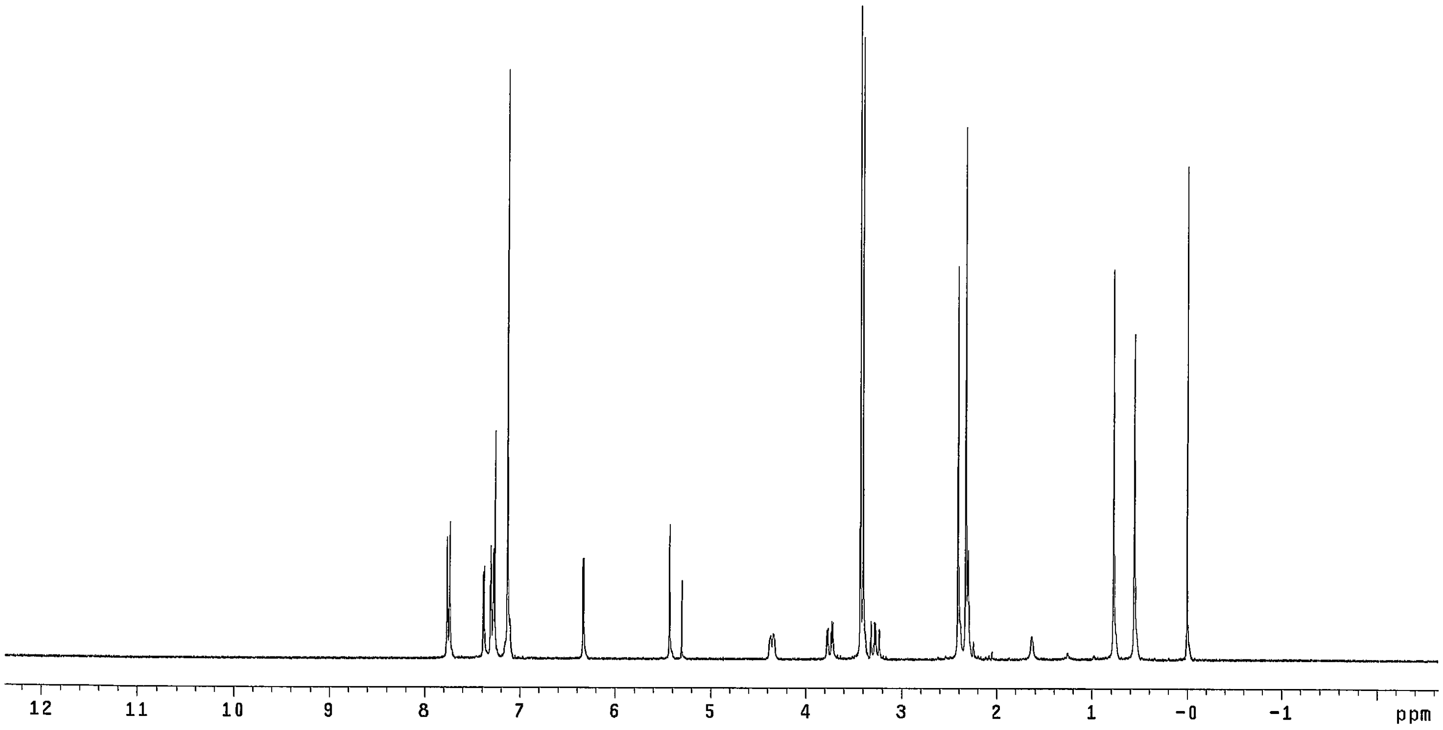

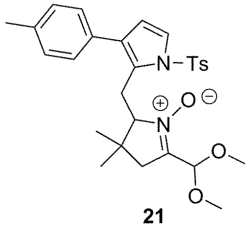

21

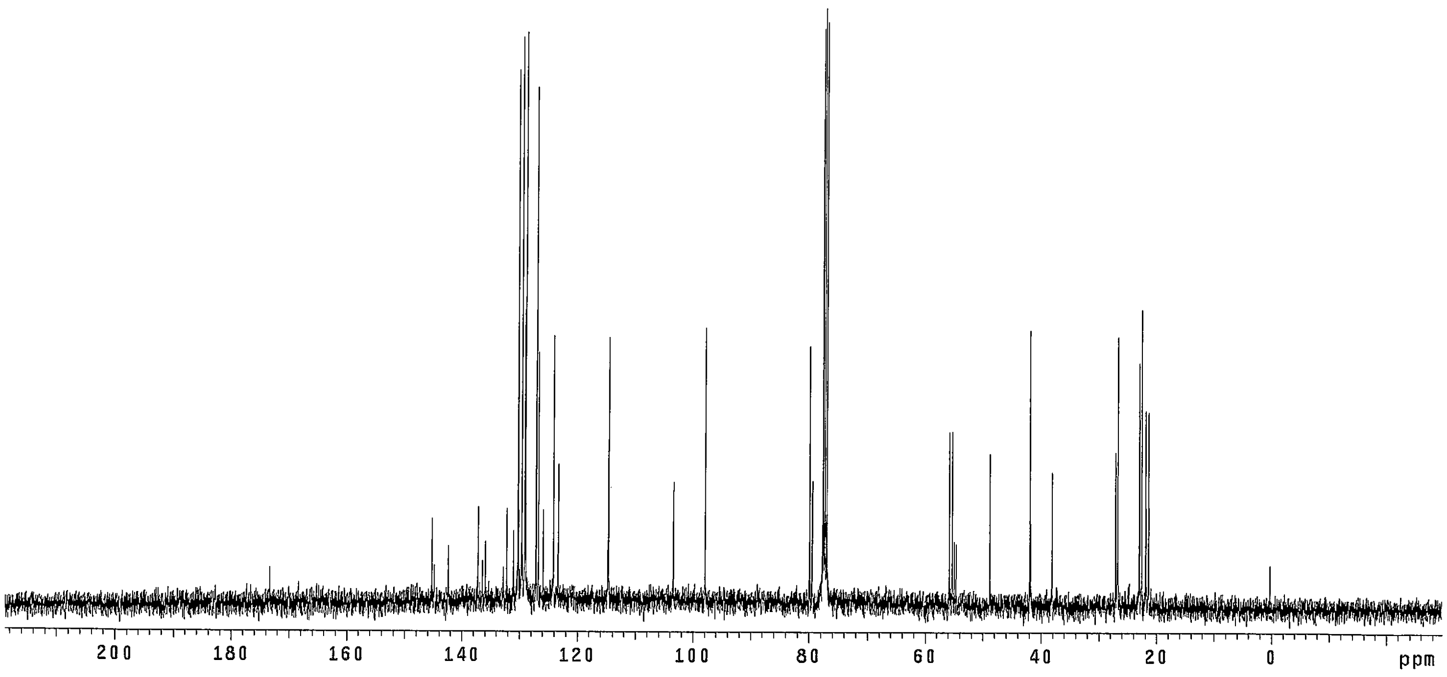

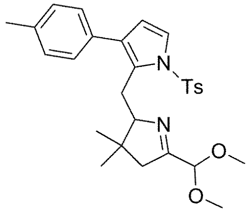

22

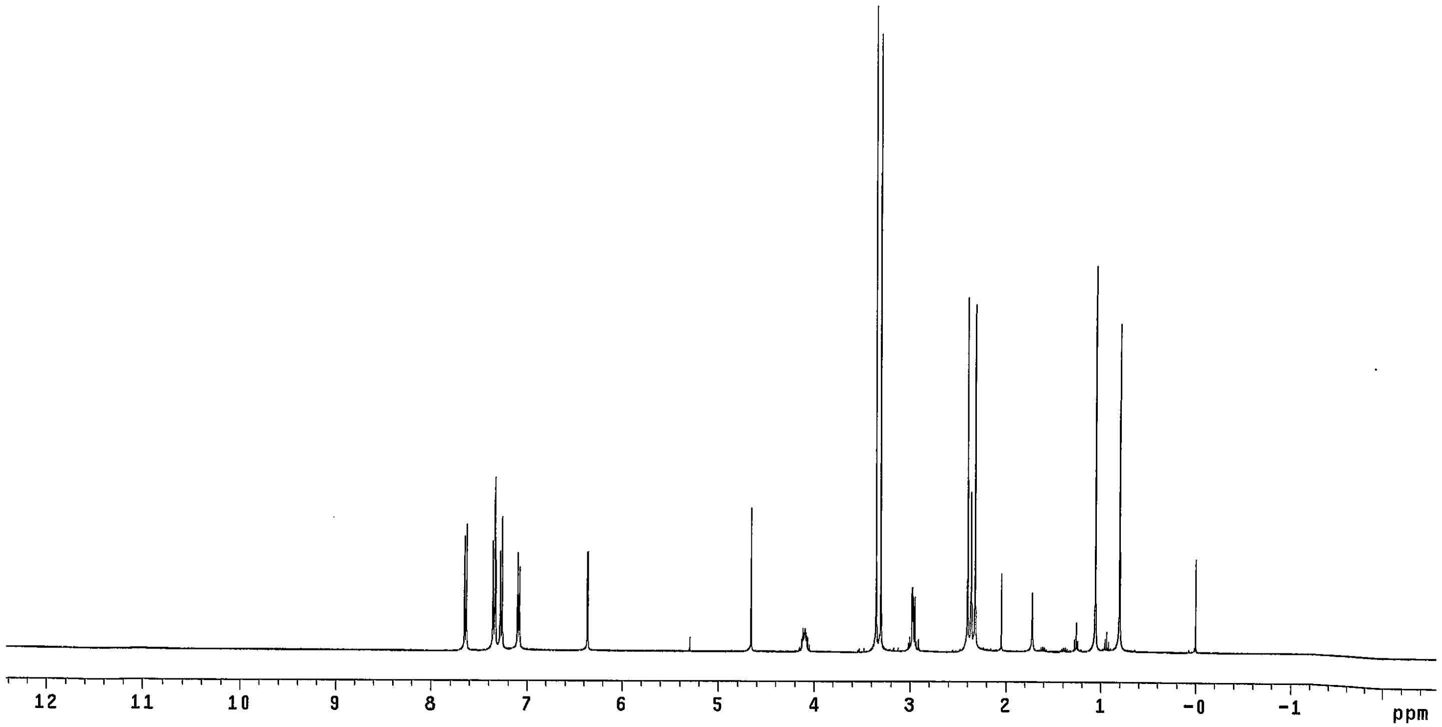

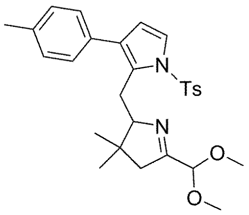

22

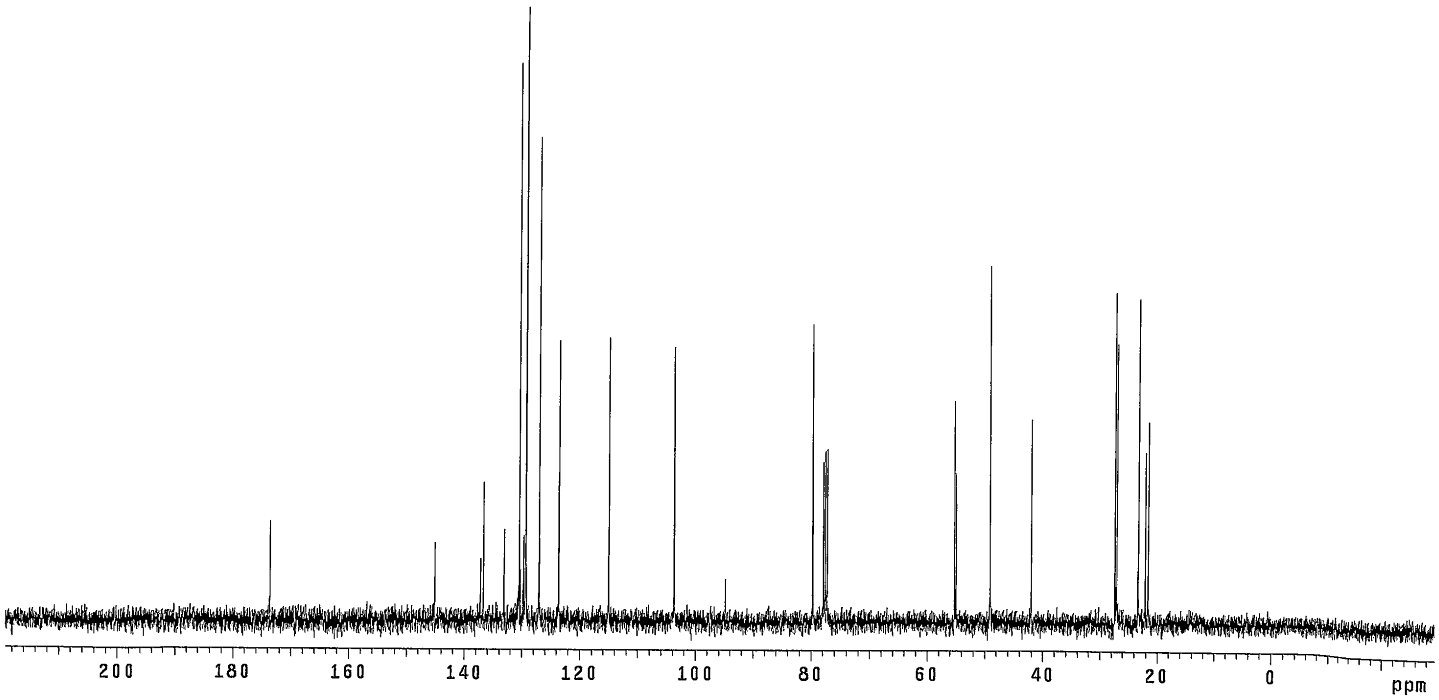

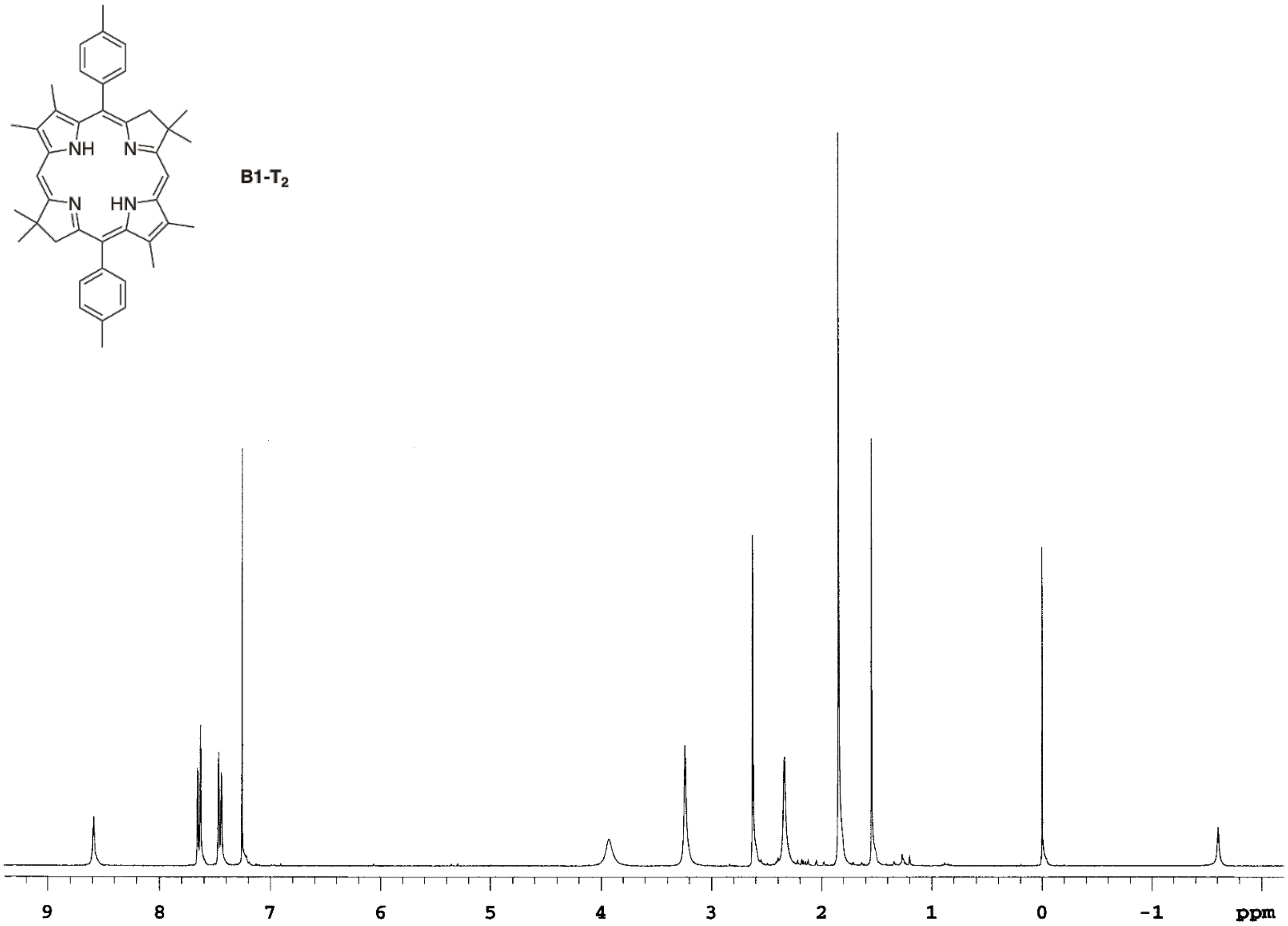

NH
N
N
HN
B1-T$_2$
9
8
7
6
5
4
3
2
1
0
-1
ppm

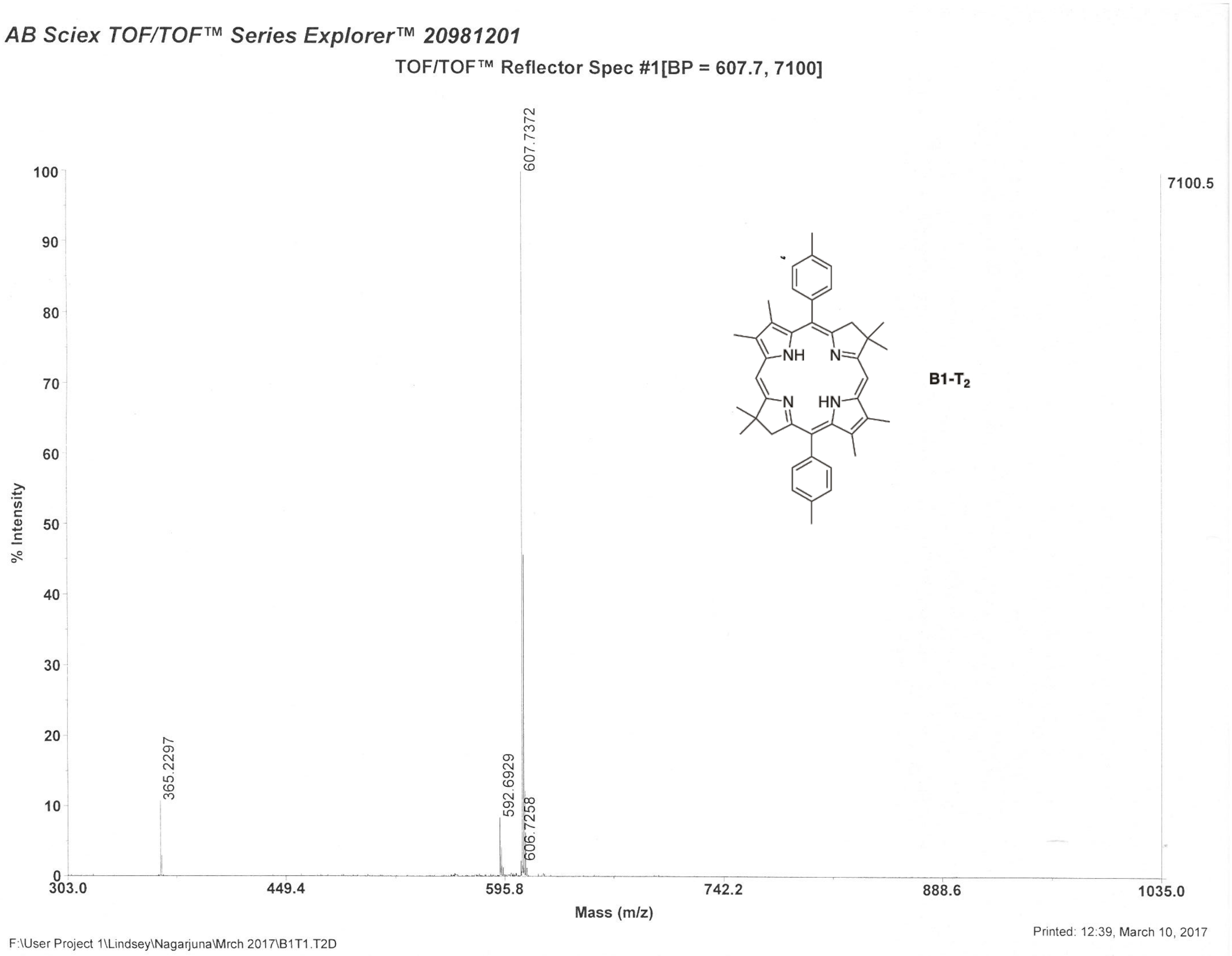
AB Sciex TOF/TOF™ Series Explorer™ 20981201
TOF/TOF™ Reflector Spec #1[BP = 607.7, 7100]
607.7372
100
90
80
70
60
50
40
30
20
10
0
7100.5
% Intensity
NH
N
N
HN
B1-T2
365.2297
592.6929
606.7258
303.0
449.4
595.8
742.2
888.6
1035.0
Mass (m/z)
Printed: 12:39, March 10, 2017
F:\User Project 1\Lindsey\Nagarjuna\Mrch 2017\B1T1.T2D

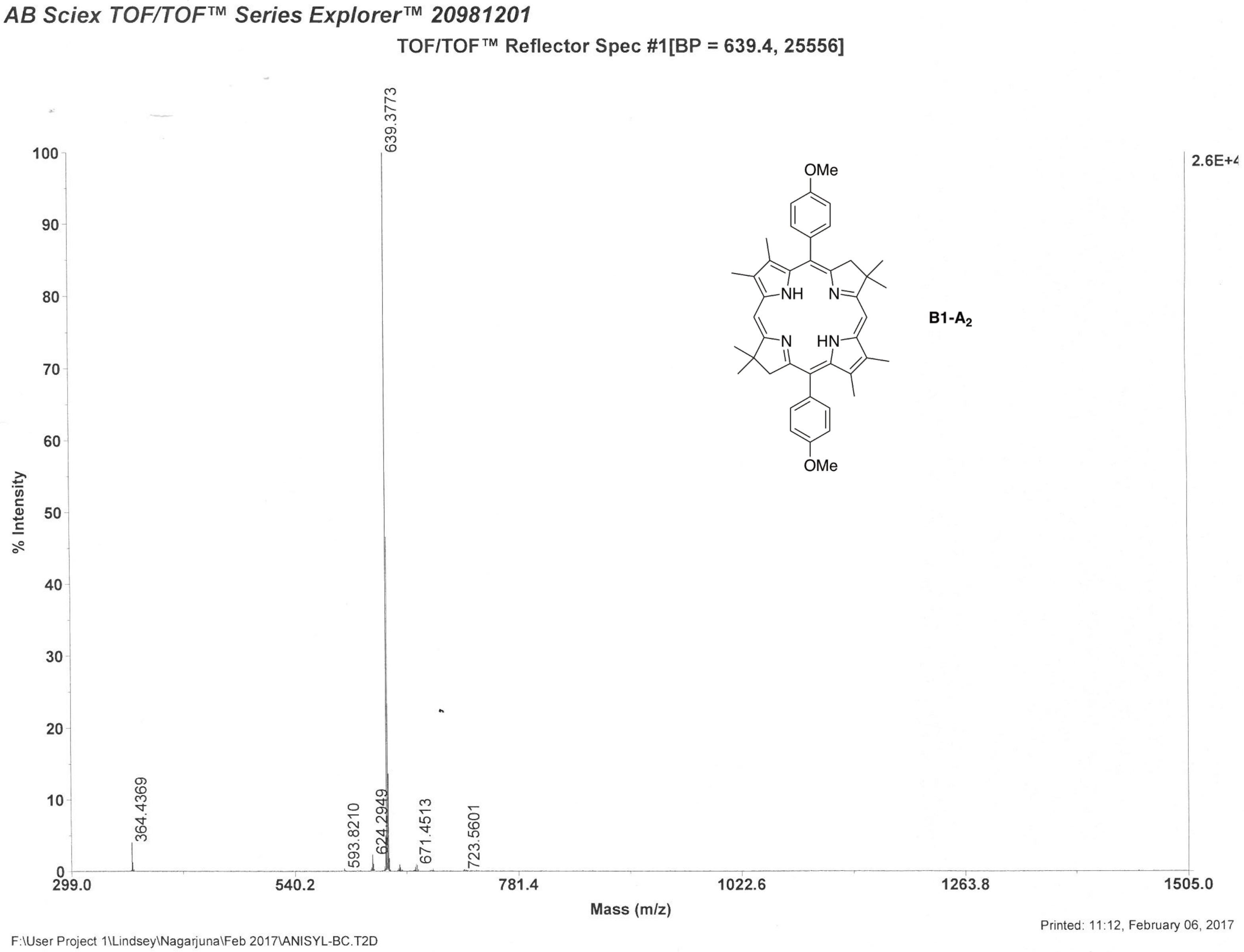
AB Sciex TOF/TOF™ Series Explorer™ 20981201
TOF/TOF™ Reflector Spec #1[BP = 639.4, 25556]
639.3773
2.6E+4
OMe
NH
N
N
HN
OMe
B1-A2
% Intensity
100
90
80
70
60
50
40
30
20
10
0
364.4369
593.8210
624.2949
671.4513
723.5601
299.0
540.2
781.4
1022.6
1263.8
1505.0
Mass (m/z)
Printed: 11:12, February 06, 2017
F:\User Project 1\Lindsey\Nagarjuna\Feb 2017\ANISYL-BC.T2D

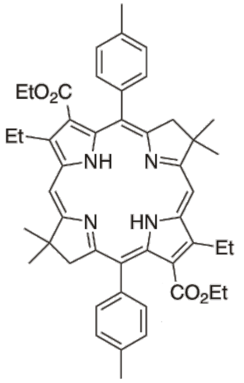

B2-T_2

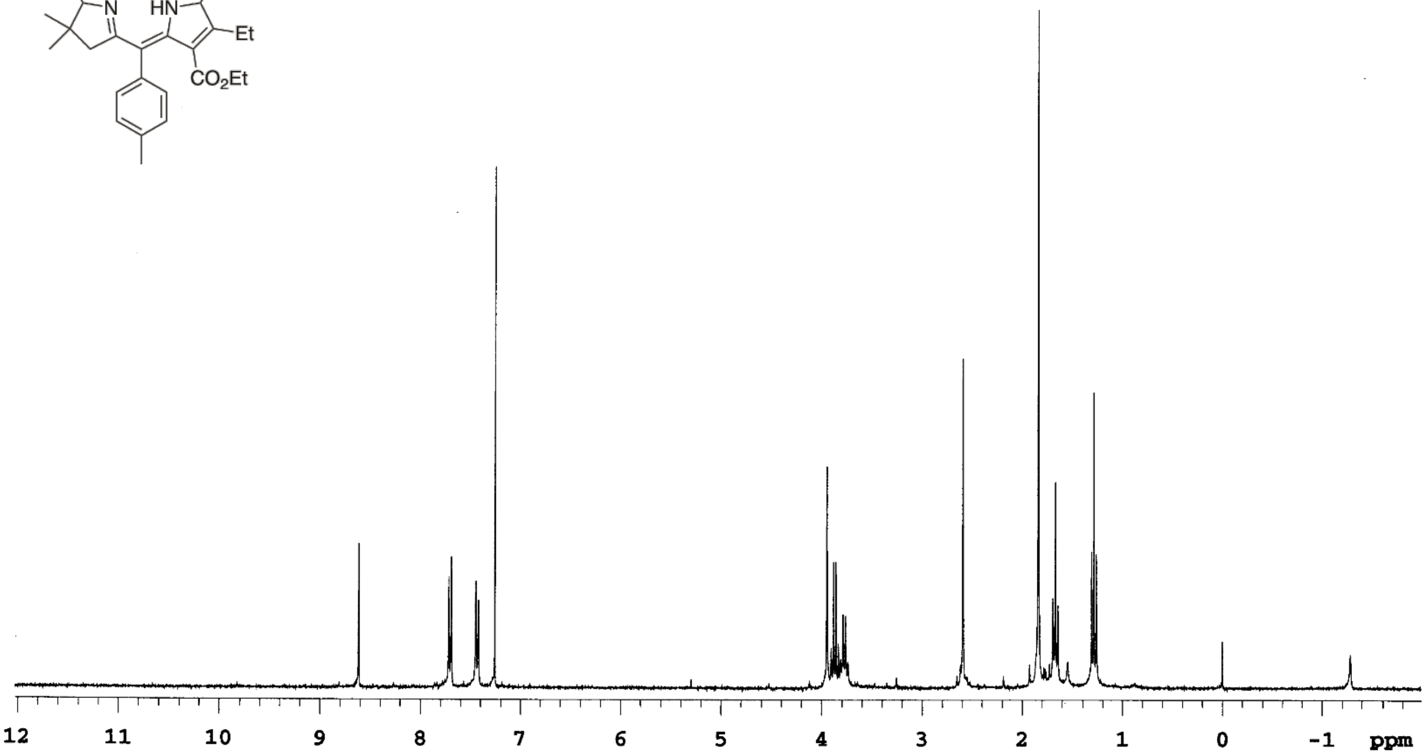

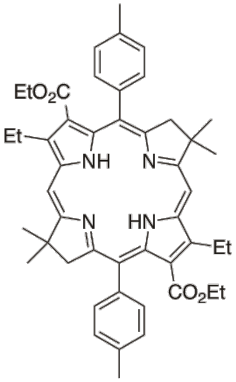
EtO2C
Et
NH
N
N
HN
Et
CO2Et

B2-T$_2$

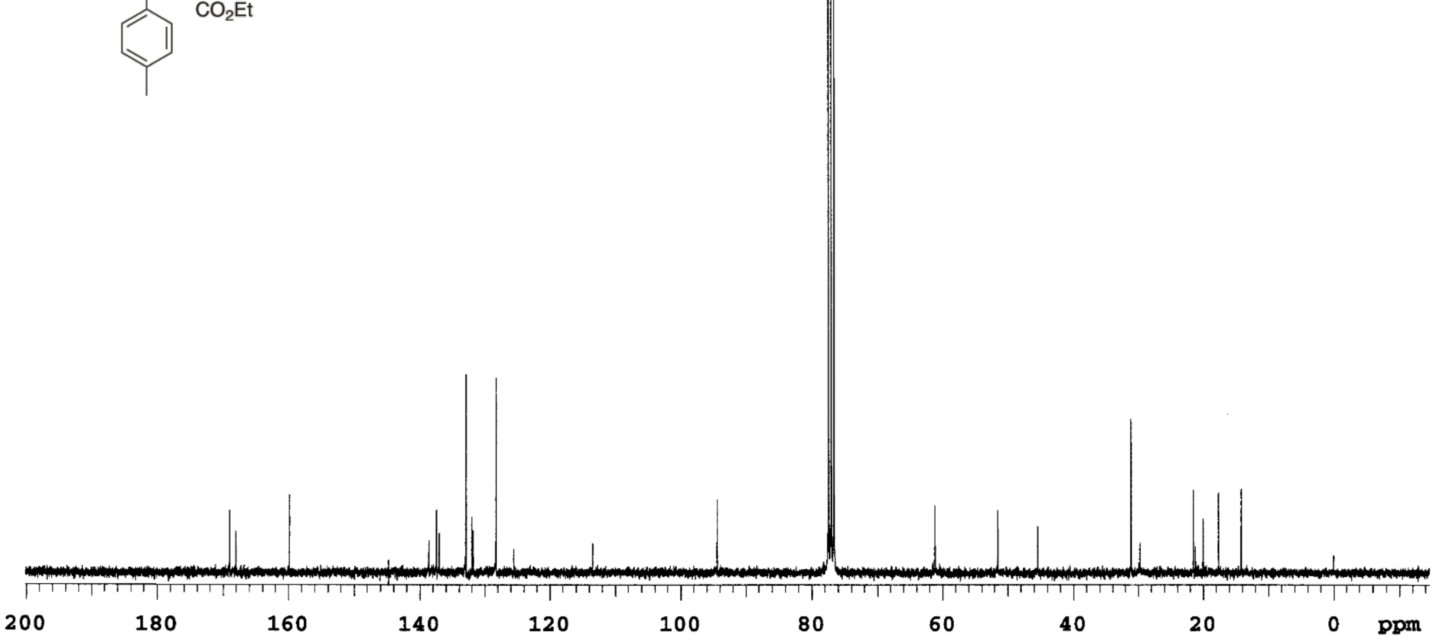
200
180
160
140
120
100
80
60
40
20
0
ppm

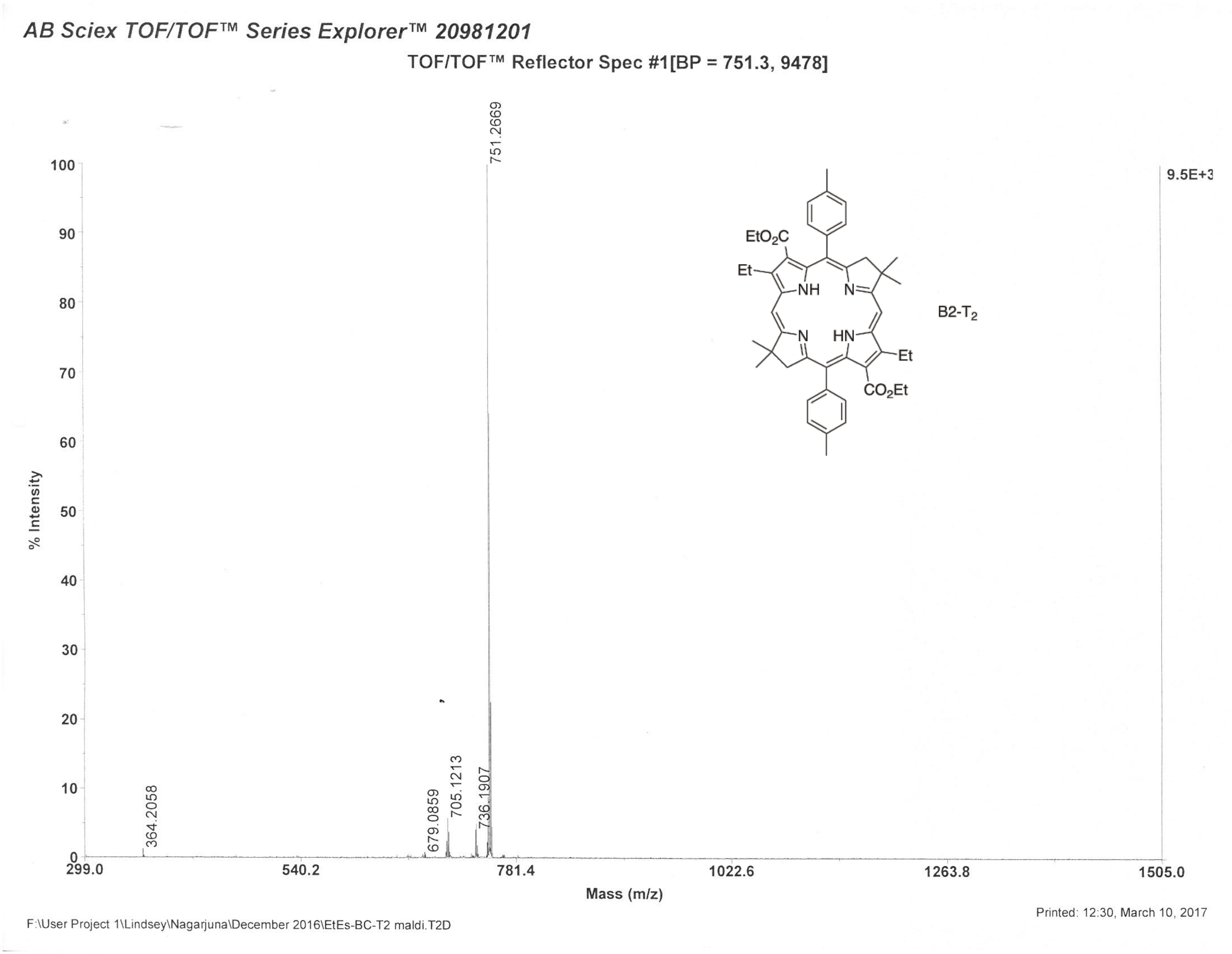
AB Sciex TOF/TOF™ Series Explorer™ 20981201
TOF/TOF™ Reflector Spec #1[BP = 751.3, 9478]
751.2669
9.5E+3
EtO2C
Et
NH
N
N
HN
Et
CO2Et
B2-T2
% Intensity
100
90
80
70
60
50
40
30
20
10
0
364.2058
679.0859
705.1213
736.1907
299.0
540.2
781.4
1022.6
1263.8
1505.0
Mass (m/z)
F:\User Project 1\Lindsey\Nagarjuna\December 2016\EtEs-BC-T2 maldi.T2D
Printed: 12:30, March 10, 2017

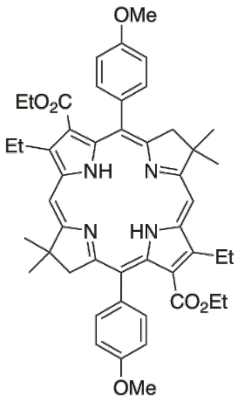

B2-A2

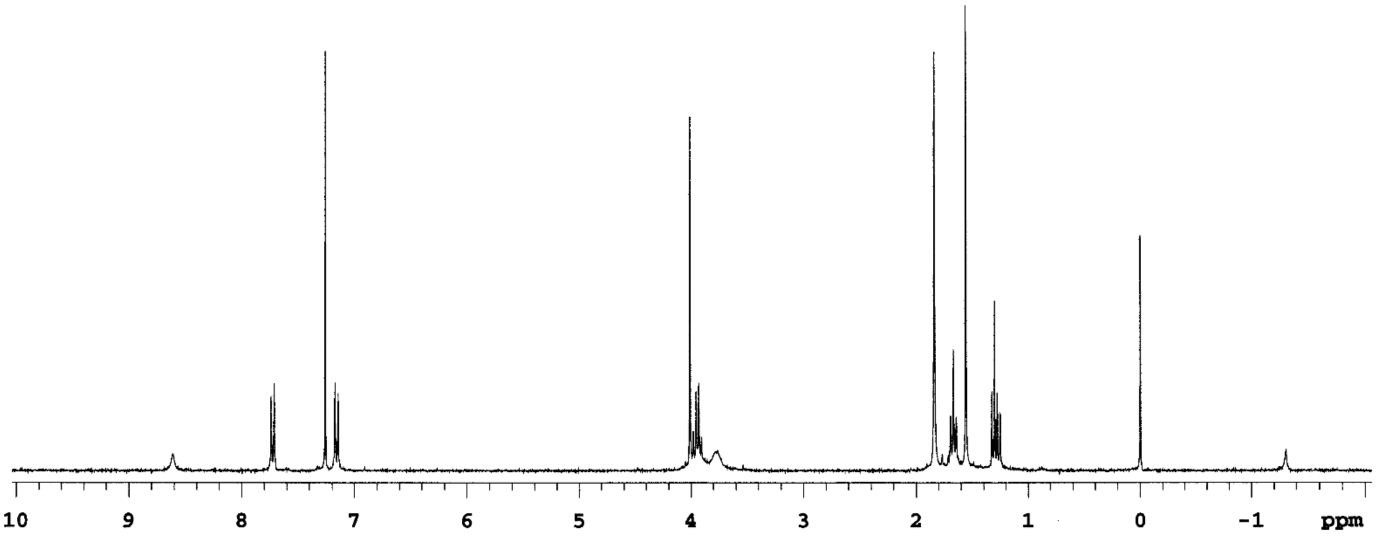

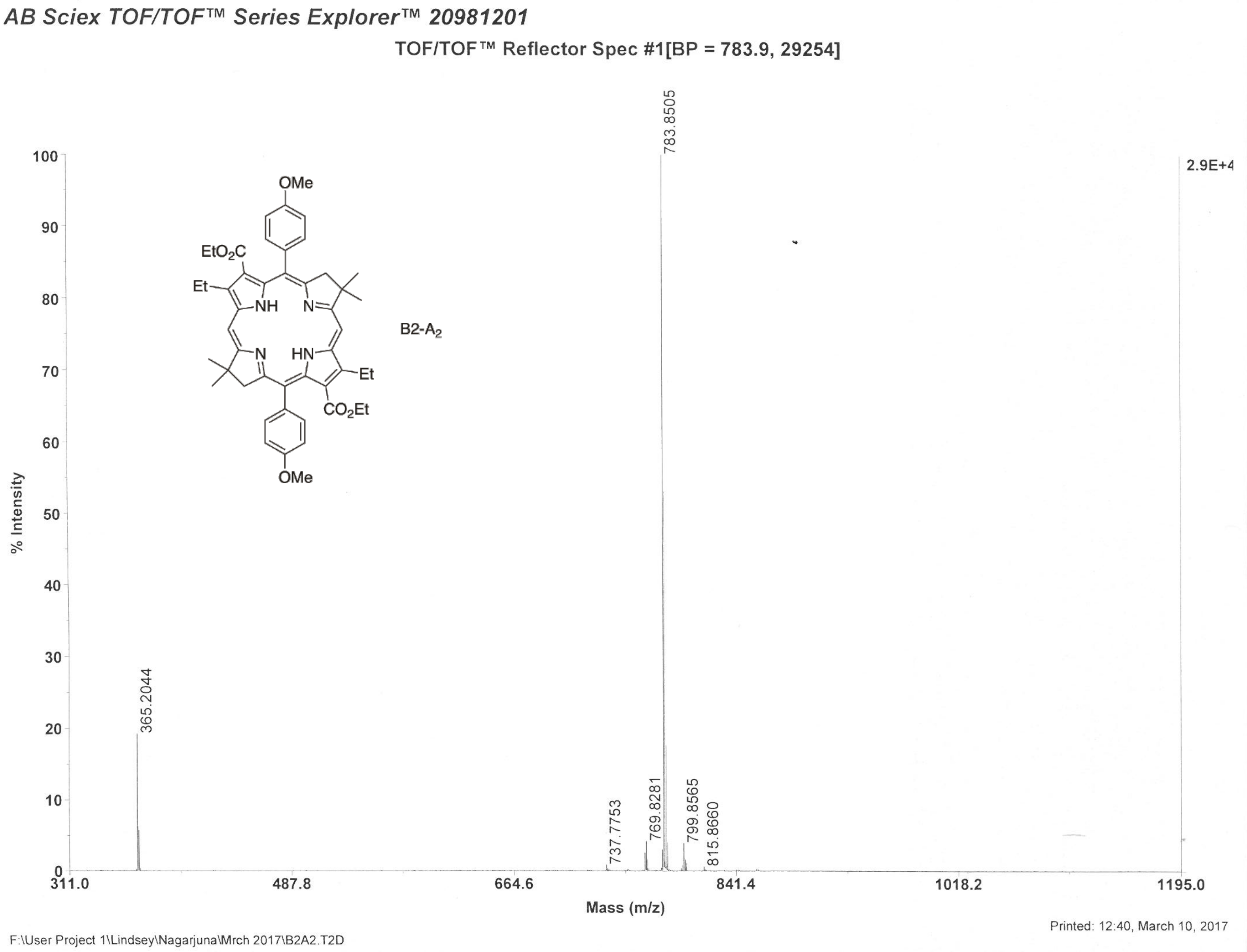
AB Sciex TOF/TOF™ Series Explorer™ 20981201
TOF/TOF™ Reflector Spec #1[BP = 783.9, 29254]
783.8505
2.9E+4
365.2044
737.7753
769.8281
799.8565
815.8660
% Intensity
Mass (m/z)
311.0
487.8
664.6
841.4
1018.2
1195.0
OMe
EtO2C
Et
NH
N
HN
CO2Et
B2-A2
Printed: 12:40, March 10, 2017
F:\User Project 1\Lindsey\Nagarjuna\Mrch 2017\B2A2.T2D

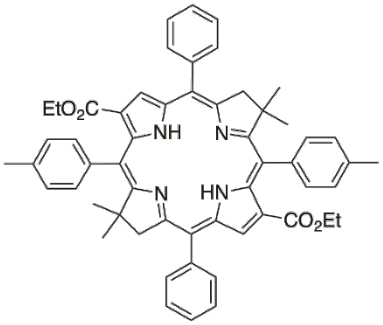

B3-P_2T_2

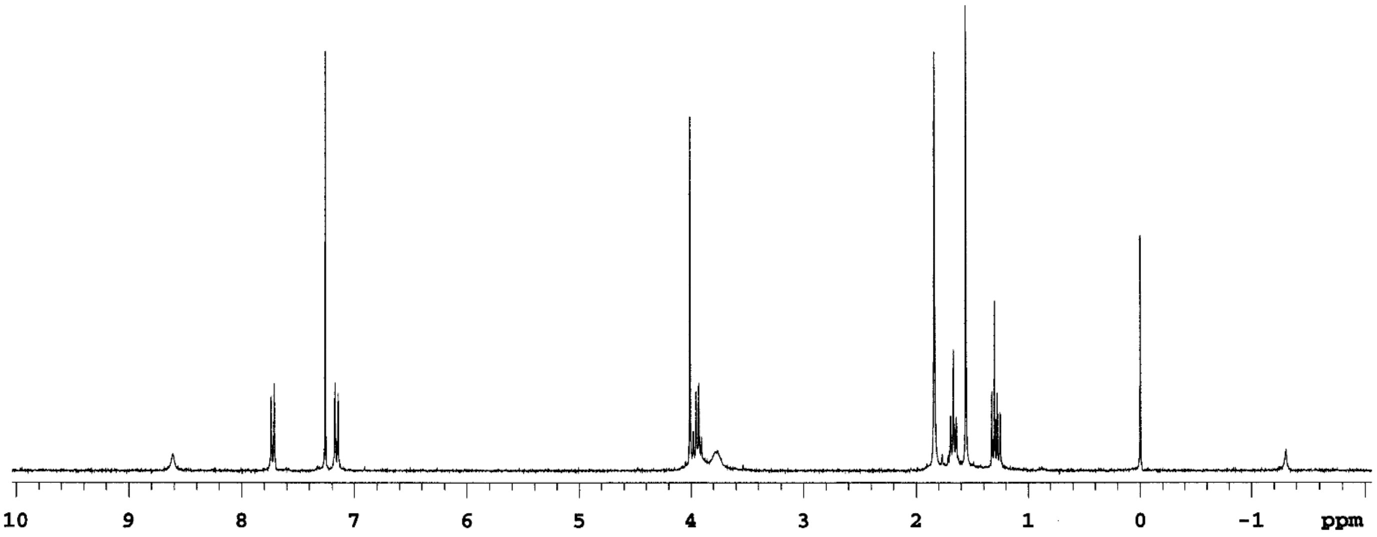

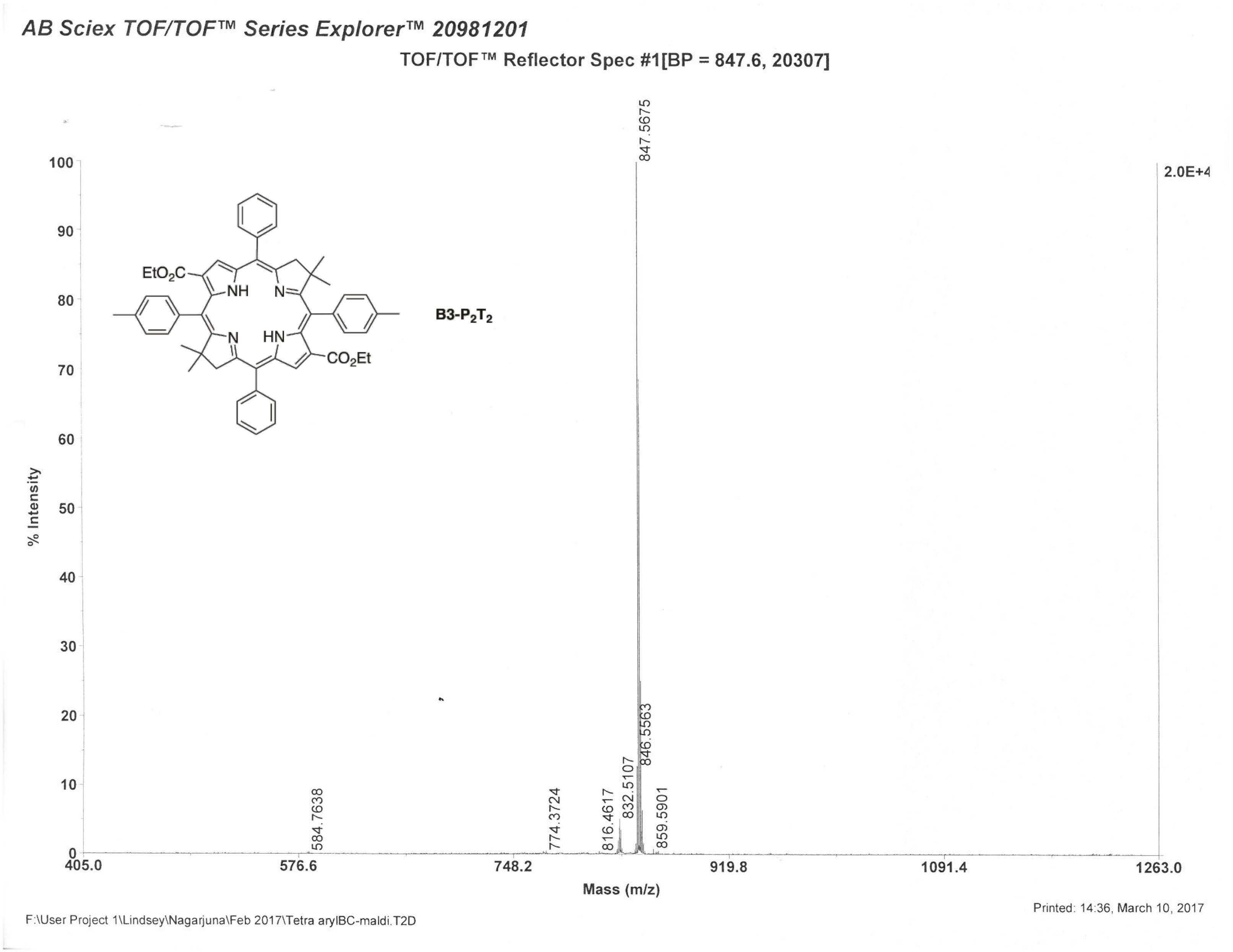

AB Sciex TOF/TOF™ Series Explorer™ 20981201
TOF/TOF™ Reflector Spec #1[BP = 847.6, 20307]
847.5675
2.0E+4
EtO2C
NH
N
HN
CO2Et
B3-P2T2
% Intensity
846.5563
832.5107
816.4617
859.5901
774.3724
584.7638
405.0
576.6
748.2
919.8
1091.4
1263.0
Mass (m/z)
Printed: 14:36, March 10, 2017
F:\User Project 1\Lindsey\Nagarjuna\Feb 2017\Tetra arylBC-maldi.T2D
